# RCSB protein data Bank: Next‐generation advanced search for exploration of experimental structures and computed structure models

**DOI:** 10.1002/pro.70731

**Published:** 2026-07-28

**Authors:** Yana Rose, Ronald Brown, Maria Voigt, Charmi Bhikadiya, Sebastian Bittrich, Jose M. Duarte, Joan Segura, Rachel Kramer Green, Stephen K. Burley

**Affiliations:** ^1^ Research Collaboratory for Structural Bioinformatics Protein Data Bank, San Diego Supercomputer Center; University of California La Jolla California USA; ^2^ Research Collaboratory for Structural Bioinformatics Protein Data Bank and the Institute for Quantitative Biomedicine Rutgers, The State University of New Jersey Piscataway New Jersey USA; ^3^ Rutgers Cancer Institute Rutgers, The State University of New Jersey New Brunswick New Jersey USA; ^4^ Department of Chemistry and Chemical Biology Rutgers, The State University of New Jersey Piscataway New Jersey USA; ^5^ Rutgers Artificial Intelligence and Data Science (RAD) Collaboratory, Rutgers, The State University of New Jersey Piscataway New Jersey USA; ^6^ Present address: PharmAI Discovery GmbH Dresden Germany

**Keywords:** chemical structure search, macromolecular structure, Protein Data Bank (PDB), structural bioinformatics, structure similarity search

## Abstract

The Protein Data Bank (PDB), established in 1971, is the primary global, open‐access archive for experimentally determined 3D macromolecular structures (proteins, RNA, DNA). The research‐focused RCSB.org web‐portal provides access to these data alongside more than one million machine‐learning‐predicted structure models, greatly expanding the available structural landscape. Rapid growth of both experimental and computational structures has increased the need for powerful yet accessible search tools that serve a broad and diverse scientific community. Herein, we describe a redesigned RCSB Protein Data Bank RCSB.org Advanced Search capability that supports intuitive discovery of 3D structures through a unified interface. This interface integrates annotation‐, sequence‐, and 3D structure‐based searches, embeds an interactive 3D viewer, and incorporates curated biological knowledge, such as catalytic site definitions from Mechanism and Catalytic Site Atlas and ligand‐guided structural motifs, for constructing geometry‐driven queries. A new Chemical Search tool allows definition of chemical queries via an integrated drawing tool or standard identifiers, seamlessly combining them with annotation filters. By allowing query definition directly within spatial and chemical contexts, these search interfaces reduce the need for detailed knowledge of residue numbering, chain identifiers, or external cheminformatics software. This capability enables efficient exploration of structures, chemical diversity, and structure–function relationships across all life domains. The redesigned interfaces can be accessed directly at rcsb.org/search/advanced for Advanced Search and rcsb.org/search/chemical for Chemical Search.

## 1 INTRODUCTION

The Protein Data Bank (PDB) (wwPDB Consortium, 2019), established in 1971 (Crystallography: Protein Data Bank 1971), is the single, global archive for experimentally determined three‐dimensional (3D) structures of biological macromolecules, jointly managed by the worldwide Protein Data Bank (wwPDB) organization (Berman et al., 2003). In the United States, PDB operations are carried out by the Research Collaboratory for Structural Bioinformatics Protein Data Bank (RCSB PDB) (Berman, 2000; Burley et al., 2024). RCSB PDB serves many millions of users worldwide, primarily through its public web portal at RCSB.org, which provides a comprehensive suite of online tools for searching, accessing, visualizing, and analyzing structural data.

All PDB data are freely and openly accessible under the most permissive Creative Commons CC0 1.0 Universal license. The archive currently contains >250,000 experimentally determined structures of proteins and nucleic acids. These structures span an extraordinary range of sizes and complexities, from small, single‐chain globular proteins to large macromolecular assemblies and integrative structures that cannot be resolved by a single experimental method alone (Vallat et al., 2025). Functionally, the archive encompasses enzymes, signaling proteins, transcription factors, ribosomes, viral protein assemblies, and many other classes of biomolecules. Associated chemical diversity is equally broad, including metal ions, cofactors, metabolites, peptide inhibitors, drug‐like molecules, and US FDA‐approved drugs. In addition to experimental structures, RCSB.org provides access to more than one million machine‐learning‐predicted computed structure models (CSMs) from the AlphaFold Protein Structure Database (Fleming et al., 2025) and the ModelArchive (Tauriello et al., 2025), substantially expanding the structural coverage of protein sequences available to the research community (Burley et al., 2022). Beyond atomic coordinates, RCSB.org integrates annotations on a weekly basis from approximately 50 trusted external biological data resources (e.g., UniProt [Bateman et al., 2022], SCOPe [Fox et al., 2014], and CATH [Sillitoe et al., 2021]). Together, these value‐added annotations place structural data in functional, evolutionary, and biological context, enabling users to relate 3D structures to sequence features, molecular function, and classification, making RCSB.org a living data resource.

The RCSB PDB user community spans experimental structural biologists, computational scientists, educators, and researchers in a wide variety of fields such as molecular and cellular biology, drug discovery, and biotechnology/bioengineering. Consequently, the scientific search questions posed to RCSB.org can vary widely. For example, a biologist may search for structures based on sequence similarity or biological function, whereas a medicinal chemist may begin from a small molecule or its binding site. The complexity of the data, the diversity of PDB data consumers (RCSB.org users), and myriad scientific questions highlight the need for flexible and interoperable search capabilities, with methods optimized for different query types that can be combined seamlessly. Equally important, these capabilities must be delivered through intuitive and accessible user interfaces that support efficient discovery and analysis by researchers with varying levels of structural biology expertise. Herein, we describe redesigned RCSB.org Advanced and Chemical Search interfaces that address these needs and facilitate research and discovery across fundamental biology, biomedicine, the energy sciences, and biotechnology and bioengineering.

### 1.1 Advanced search capabilities

**FIGURE 1:**
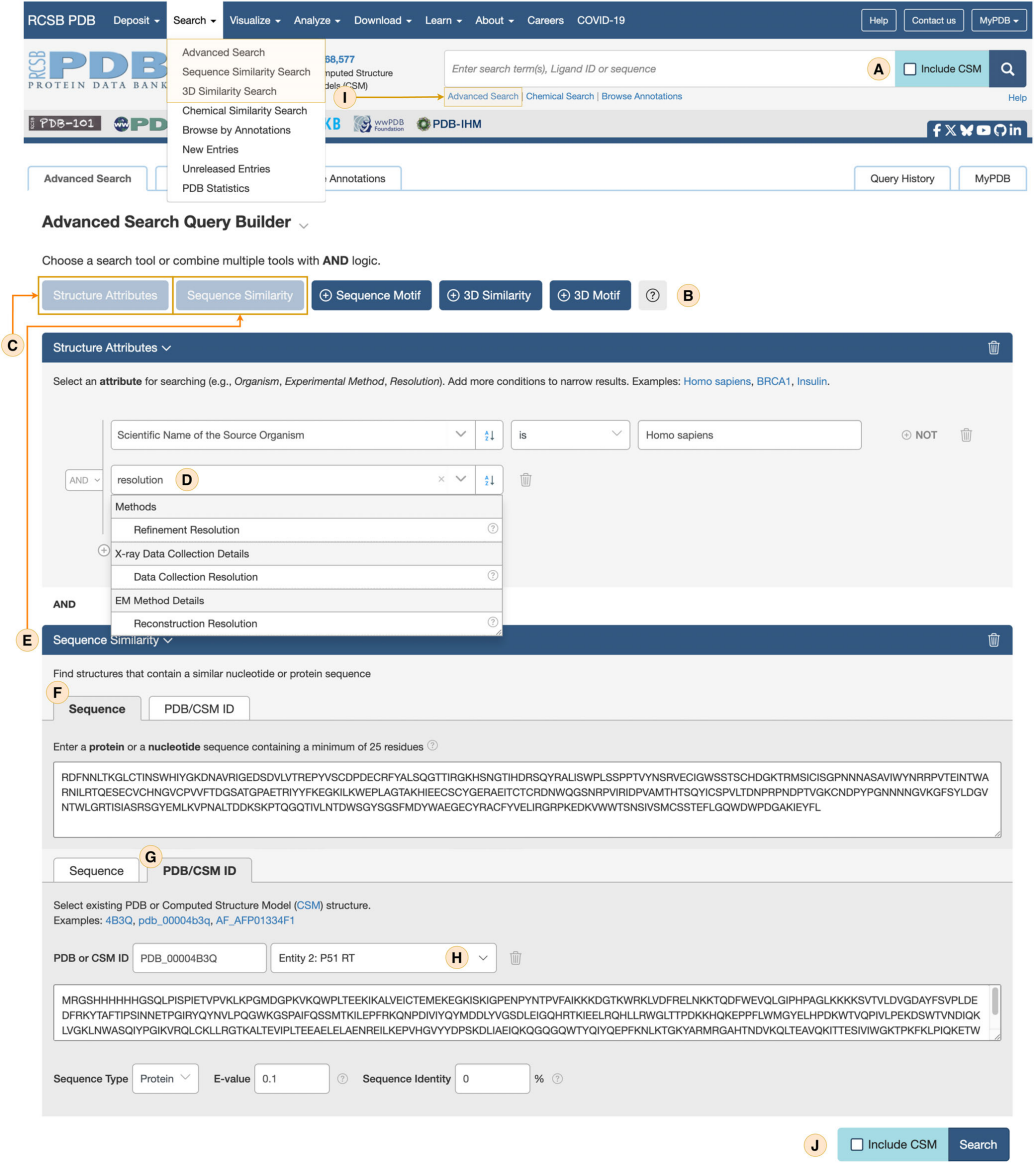
Advanced Search on RCSB.org. (a) Basic Search supports keyword‐driven, full‐text searches. (b) Search tools bar allows selection and combination of multiple search modes, each purpose‐built to query a specific type of data (e.g., structured metadata, sequences, or 3D coordinates). (c) Structure Attributes search queries on available 3D macromolecular structure metadata. (d) Attribute selection menu lists hundreds of metadata items and supports filtering. (e) Sequence Similarity search for global homology‐based matching. (f) Interface for directly pasting a polymer sequence. (g) Interface for selecting a polymer sequence from an existing PDB or CSM entry. (h) Dropdown menu showing available sequences for the selected entry. (i) Access points to open the Advanced Search interface from the global site navigation. (j) The “Include CSM” control allows users to include available CSMs alongside experimentally determined structures in the search results.

Advanced Search, available at rcsb.org/search/advanced, enables users to build precise queries for retrieving macromolecular 3D structures. Unlike the keyword‐based Basic Search (Figure 1a), which performs broad full‐text matching, Advanced Search supports metadata‐driven filtering, explicit Boolean logic, and integration of diverse bioinformatic search tools. Each search tool is purpose‐built to query a specific aspect of structural data—such as metadata, polymeric sequences, 3D geometry, or functional motifs. These capabilities are essential for exploring structural, functional, and evolutionary relationships between and among 3D biostructures. The search tools bar (Figure 1b) provides direct access and can combine multiple search modes within a single query.

Structure Attributes search (Figure 1c) is the foundation for metadata‐driven discovery of the 3D biostructures. It enables queries on structured metadata defined in the PDBx/mmCIF (Westbrook et al., 2022) dictionary and its companion extensions, IHMCIF (Vallat et al., 2024) and ModelCIF (Vallat et al., 2023). These metadata include deposition details, such as structure authorship, experimental method, data collection and processing, biological sample description, and release date, as well as value‐added annotations integrated from external biological resources (e.g., Gene Ontology terms and Enzyme Classification). The attribute list contains hundreds of searchable fields and can be interactively filtered to simplify navigation and quickly locate relevant attributes (Figure 1d).

Sequence Similarity search (Figure 1e) supports identification of related proteins or nucleic acids based on sequence similarity matching methods. This search mode uses MMseqs2 software (Steinegger & Söding, 2017) to perform global homology‐based searches across polymer sequences. Users may define a query by directly pasting a protein, RNA, or DNA sequence into the input field (Figure 1f), which is particularly useful for newly determined, uncharacterized, or hypothetical sequences for which structural or functional annotations are limited. Alternatively, users can use a polymer sequence from a known PDB structure or CSM by providing a structure identifier (Figure 1g). All associated polymers are then presented in a selectable list using descriptive names (Figure 1h), eliminating the need to manually locate or copy chain sequences.

Sequence Motif search enables detection of sequence features independent of overall sequence similarity. Users can define motifs using simple residue queries, PROSITE‐style patterns, or regular expressions for more complex patterns. For example, a simple XPPXP query can identify SH3 domain–binding motifs (Kay et al., 2000); a PROSITE‐style pattern N‐{P}‐[ST]‐{P} can locate potential N‐linked glycosylation sites; and a regular expression C.{2,4}C.{12}H.{3,5}H can capture zinc finger C2H2 motifs with variable spacing between residues.

3D Similarity search supports shape‐based matching at the polymer chain or biological assembly level, enabling identification of related macromolecules even in the absence of detectable sequence similarity. This capability is particularly valuable for uncovering structural relationships arising from convergent evolution, functional constraints, or extensive sequence divergence, which are often missed by sequence‐based approaches. The method employs a structure similarity strategy that transforms 3D coordinates into fixed‐length vector representations using protein language models in combination with a deep neural network architecture (Segura et al., 2026), enabling efficient large‐scale comparisons. Importantly, similarity detection is accurate across length scales: domain, chain, or assembly levels. In benchmarking, this embedding‐based approach achieves sensitivity comparable to Foldseek (van Kempen et al. 2024) across domain, full‐chain, and assembly searches, though it does not reach the sensitivity of computationally intensive alignment‐based methods such as US‐align (Zhang et al., 2022) and DALI (Holm and Sander 1993). In turn, it offers far greater scalability, returning results across millions of structures in near real time and remaining tractable at the scale of hundreds of millions of predicted models. Users should be aware of one known limitation at the assembly level: because per‐residue embeddings are computed independently for each chain and then concatenated, assemblies built from identical or highly similar subunits but arranged in different quaternary configurations can result in very similar embeddings, occasionally producing false positives wherein the constituent chains match closely but the overall assembly architecture differs.

3D Motif search enables identification of functionally relevant residue arrangements, in which biological activity is determined by the precise spatial configuration of a small number of residues, rather than by the overall fold. Many biological questions center on such localized structural features that define catalytic activity, ligand recognition, or cofactor binding, and have motivated both classic and recent computational approaches for residue‐level 3D motif discovery (Kim et al. 2025 Jul 6). Examples include calcium‐binding EF‐hand motifs, where conserved acidic residues coordinate a bound metal ion, which induces conformational changes across a large family of calcium‐sensing proteins (Gifford et al., 2007); ATP‐binding P‐loop motifs that position phosphate groups for catalysis in diverse NTP‐binding enzymes (Saraste et al., 1990); and heme‐binding pockets in cytochromes and other hemoproteins, where specific histidine or cysteine side chains anchor the cofactor in a well‐defined binding environment (Li et al., 2011). By comparing 3D positions of user‐defined residues, 3D motif search detects conserved functional sites across proteins with divergent sequences or overall structures (Bittrich et al., 2020).

Advanced Search can be accessed from the global RCSB.org navigation (Figure 1i) or via contextual query examples throughout the website (e.g., search links on Structure Summary Pages). When opened without predefined input, the interface presents blank forms for interactive query building. URL‐encoded query links automatically pre‐populate the interface with specified search criteria. An explicit “Include CSM” control (Figure 1j) allows users to include available CSMs alongside experimentally determined structures in the search results. User‐defined queries are translated into structured API requests and executed against the RCSB PDB Search API service (search.rcsb.org) (Bittrich et al., 2023), which supports programmatic search and retrieval of matching entries.

### 1.2 Visualization‐assisted construction of 3D structural queries

**FIGURE 2:**
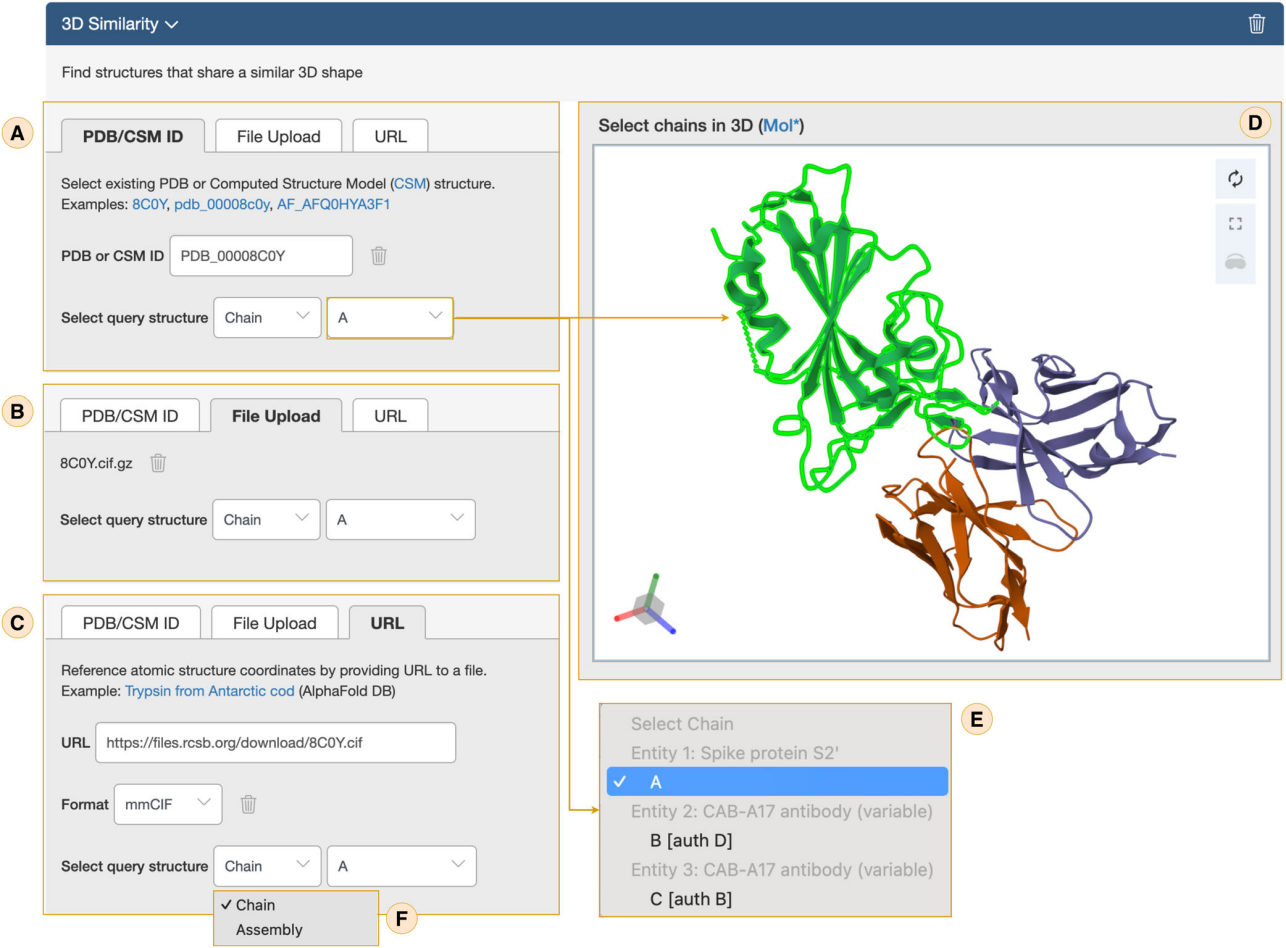
Structure referencing and selection for 3D Similarity search, illustrated using the spike protein S2 (chain A, highlighted in green) from the severe acute respiratory syndrome coronavirus 2 (SARS‐CoV‐2) Omicron variant structure (PDB ID: pdb_00008c0y (Sheward et al., 2024)). (a) Direct lookup of reference structures using PDB or CSM structure identifiers. (b) Upload of user‐supplied coordinate files from a local system. (c) Resolution of reference structures from a persistent external link. (d) Embedded Mol* viewer displaying the referenced structure and supporting interactive selections. (e) Dropdown menu for selecting polymer chains within the query form. (f) Control for switching the query unit from polymer chain to biological assembly.

A major enhancement of the redesigned Advanced Search interface is its integration with the Mol* molecular viewer (Sehnal et al., 2021), which allows users to build 3D structural queries through an interactive, visualization‐guided workflow rather than relying solely on textual descriptors. Both the 3D Similarity and 3D Motif search interfaces support multiple modes for specifying reference structures, including direct lookup of PDB or CSM entries using structure identifiers (e.g., pdb_00008c0y; Figure 2a) and submission of user‐supplied coordinate files via local upload (Figure 2b) or persistent external URLs (Figure 2c). These options allow users to compare unpublished or externally generated structures against the entire contents of the PDB archive without requiring prior deposition of atomic coordinates.

Referenced structures are automatically loaded and rendered in an embedded Mol* viewer (Figure 2d), enabling interactive selections. Selections made within the 3D viewer are synchronized with the query builder, where the corresponding structural identifiers are displayed and incorporated into the underlying search request. In 3D Similarity search, selections are performed at the polymer chain level by default (Figure 2e), with the option to switch the query unit to biological assemblies (Figure 2f). In contrast, 3D Motif search supports residue‐level selection within polymeric chains, reflecting its focus on defining spatial arrangements of specific residues that constitute functional sites.

By allowing queries to be defined directly through visual interaction with molecular structures, users are not required to work with formal chain identifiers or residue numbering schemes. Consequently, the new search interface lowers the barrier to constructing 3D searches and makes structure‐based querying accessible to users regardless of their level of structural biology expertise.

### 1.3 3D motif templates for catalytic residues

A key new capability in the redesigned interface is the ability to execute 3D motif queries using templates sourced from the Mechanism and Catalytic Site Atlas (M‐CSA) (Ribeiro et al., 2017). M‐CSA is a valuable structural resource that organizes experimentally validated catalytic sites into annotated enzyme families. Each M‐CSA entry represents a functional family of enzymes that share a common catalytic mechanism and lists the key residues defining a conserved catalytic activity supported by literature‐backed functional evidence and expert annotation. Integration within Advanced Search supports one‐click construction of 3D motif queries based on family‐level catalytic residues.

**FIGURE 3:**
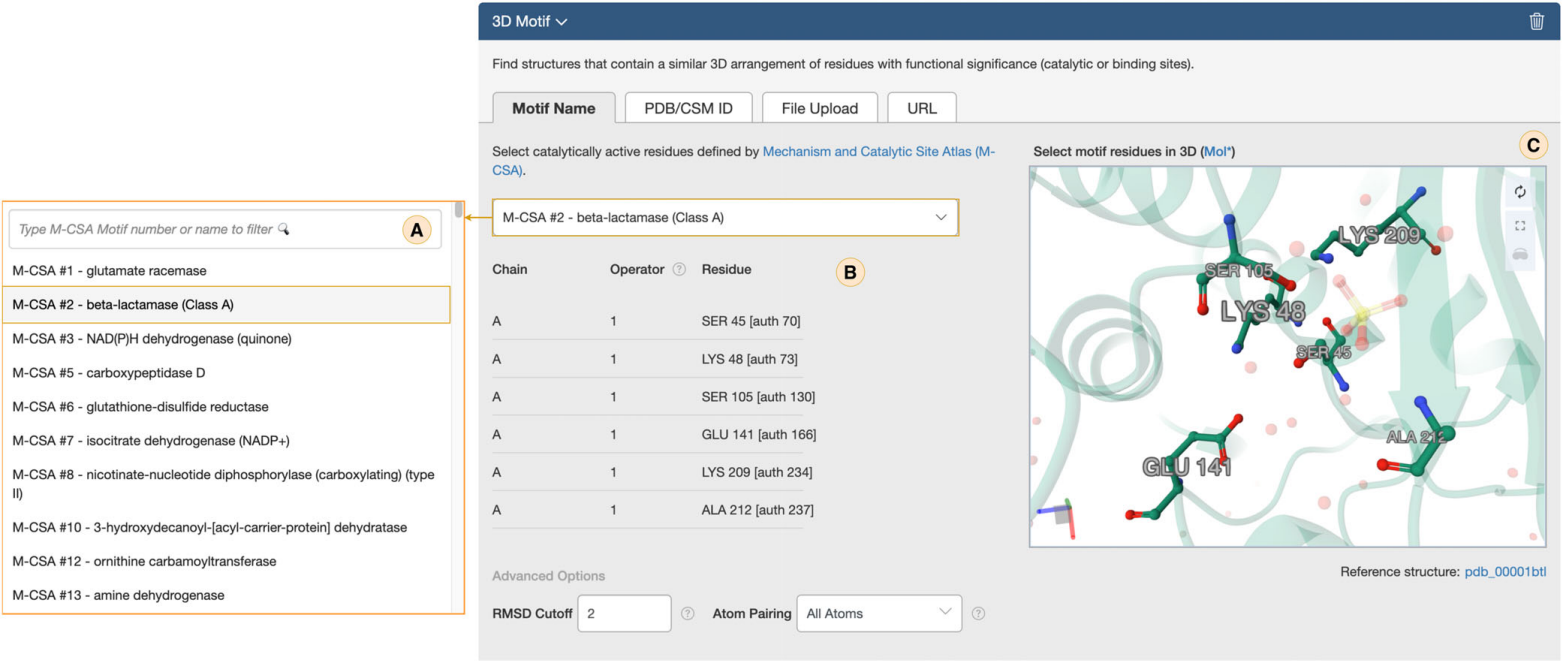
M‐CSA–guided catalytic motif selection and visualization using beta‐lactamase (Class A) as an example (M‐CSA ID: 2). (a) Search‐and‐select control for choosing curated catalytic site templates by M‐CSA entry name or by M‐CSA identifier. Note that motif textual lookup is supported by typing in the available text box. (b) M‐CSA template catalytic residues populated into the 3D motif query form. (c) Integrated Mol* viewer rendering the resolved catalytic motif for visual inspection, with catalytic residues highlighted to provide spatial context within the parent structure.

Motif selection is performed through a search‐and‐select control (Figure 3a), which lists available templates by M‐CSA entry name—typically the protein or enzyme family name—and by stable M‐CSA identifier (e.g., M‐CSA #2‐beta‐lactamase (Class A)). Upon selection, the interface resolves the chosen template into a list of catalytic residues that are automatically populated into the query form (Figure 3b) and rendered in the integrated Mol* viewer for visual inspection (Figure 3c). Within the viewer, motif residues are displayed using a dedicated representation in which side chains are shown as ball‐and‐stick and individually labeled to facilitate interpretation of residue identity and spatial arrangement. To enhance visual clarity while preserving structural context, the remainder of the macromolecular structure is rendered semi‐transparent. This visualization strategy emphasizes the geometry and composition of the functional motif while retaining its placement within the full 3D structure.

By supporting motif lookup at the family level using familiar biological names, the interface eliminates the need for users to manually identify specific residues or infer catalytic geometry from individual PDB entries. This approach directly incorporates expert‐curated knowledge into the 3D motif query construction workflow.

### 1.4 Ligand‐based 3D motif selection

**FIGURE 4:**
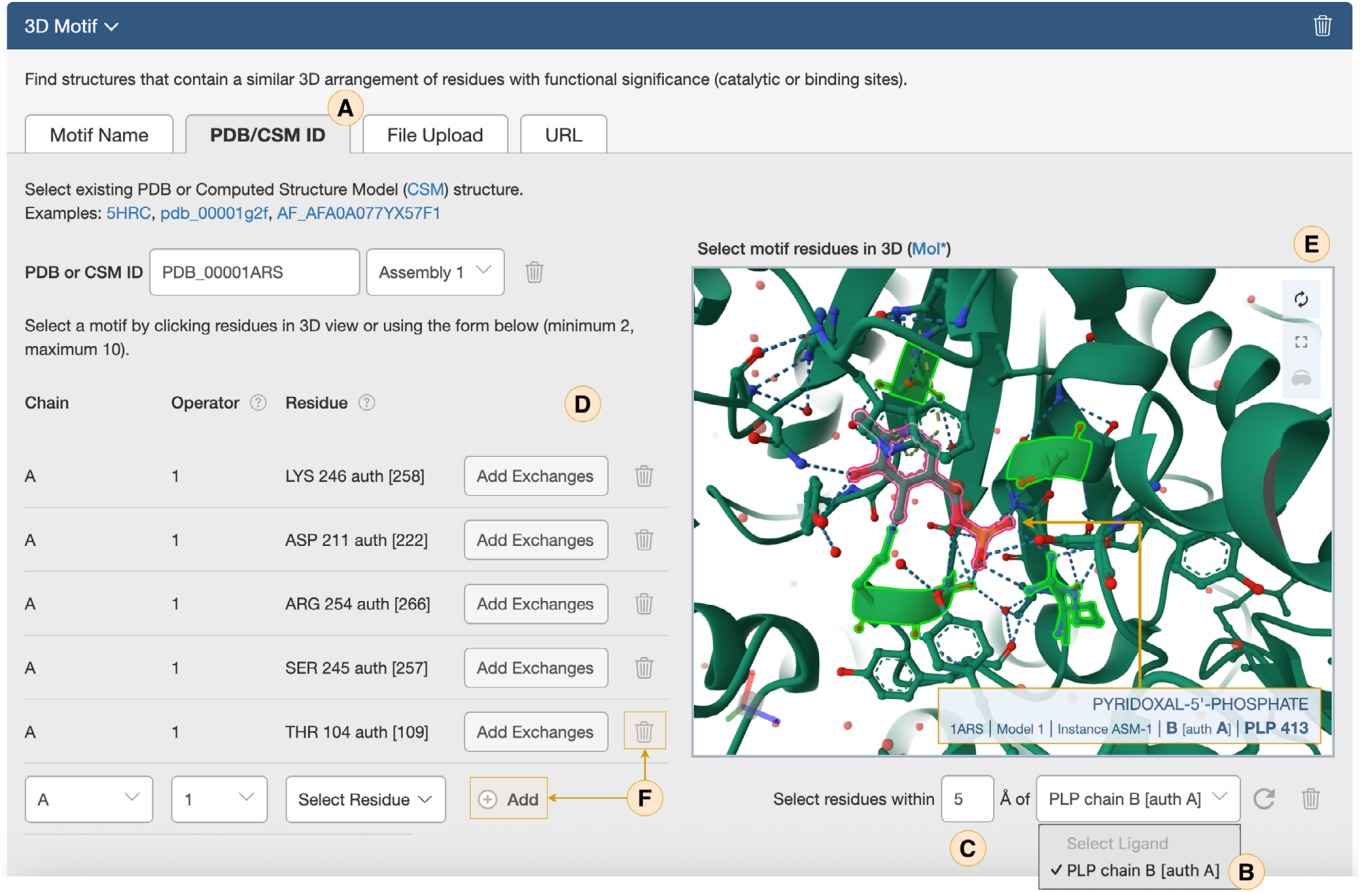
Ligand‐guided 3D motif selection for PLP‐dependent aminotransferase (PDB ID: pdb_00001ars (Okamoto et al., 1994)). (a) Structure selection mode, where a PDB entry is chosen as the source of experimentally observed macromolecule–ligand interactions. (b) Ligand selection dropdown listing all ligand instances bound to the selected structure. (c) Spatial radius control used to define the distance threshold for identifying residues interacting with the selected ligand. (d) Query form displaying the selected set of residues. (e) Integrated Mol* 3D viewer visualizing the selected ligand and its surrounding residues, with interacting residues shown in a ball‐and‐stick representation; residues included in the 3D motif query are highlighted in light green. (f) Interactive editing controls can refine the motif definition by adding or removing residues.

The redesigned Advanced Search UI further supports ligand–based residue selection, enabling 3D motif queries derived directly from experimentally observed macromolecule–ligand interactions in PDB structures (Figure 4a). Within the interface, ligand instances bound to a selected structure are presented in a dedicated dropdown menu (Figure 4b, bottom right). Users may select a ligand and define a spatial radius that determines the proximity threshold for identifying interacting residues (Figure 4c). Residue selection is based on atom‐level proximity: if any atom of a polymeric residue falls within the specified radius of any ligand atom, the entire residue is included in the selection. Up to 10 residues closest to the ligand are automatically included in the query.

The freedom to adjust the radius is critical. Different classes of molecular interactions operate over distinct characteristic length scales. For example, covalent bonds and coordination interactions typically occur at shorter distances, whereas hydrogen bonding, electrostatic interactions, and hydrophobic contacts may involve residues positioned several Ångström units (Å) away from the ligand. Radius customization, therefore, enables users to tailor residue selection to the underlying biochemical interaction type and functional context. This feature enables users to define 3D motif queries grounded in ligand interaction context without requiring manual specification of residue identifiers.

Selected residues are listed in the query form (Figure 4d) and simultaneously visualized in the integrated Mol* viewer (Figure 4e). Users can further refine the selection by adding or removing residues in the 3D view or by editing the form directly (Figure 4f). In addition to automatic selection, clicking on a ligand displays its local interaction environment in the Mol* viewer, highlighting nearby residues within a 5 Å radius in a ball‐and‐stick representation and showing both covalent and non‐covalent interactions. Anchoring residue selection in experimentally observed biochemical interactions supports single‐click construction of 3D motif queries targeting functional binding sites, thereby improving the biological relevance of these searches.

N.B.: The maximum number of residues in a selection is 10. This limit reflects a tradeoff between search sensitivity and computational cost. Each additional residue introduces another geometric constraint that candidate matches must satisfy; because these constraints compound multiplicatively, even small per‐residue deviations can substantially reduce the probability of recovering otherwise valid true‐positive matches as the motif size increases, while also increasing the computational complexity of the search.

### 1.5 Search results exploration

The redesigned search results interface supports analysis of matches at multiple levels of biomolecular organization. As structural biology questions may be addressed using different structural units, including deposited models, polymer sequences, biological assemblies, and bound ligands, the interface provides complementary result views tailored to these distinct perspectives. Search results are presented through four views: Structures, Macromolecules, Assemblies, and Ligands.

**FIGURE 5:**
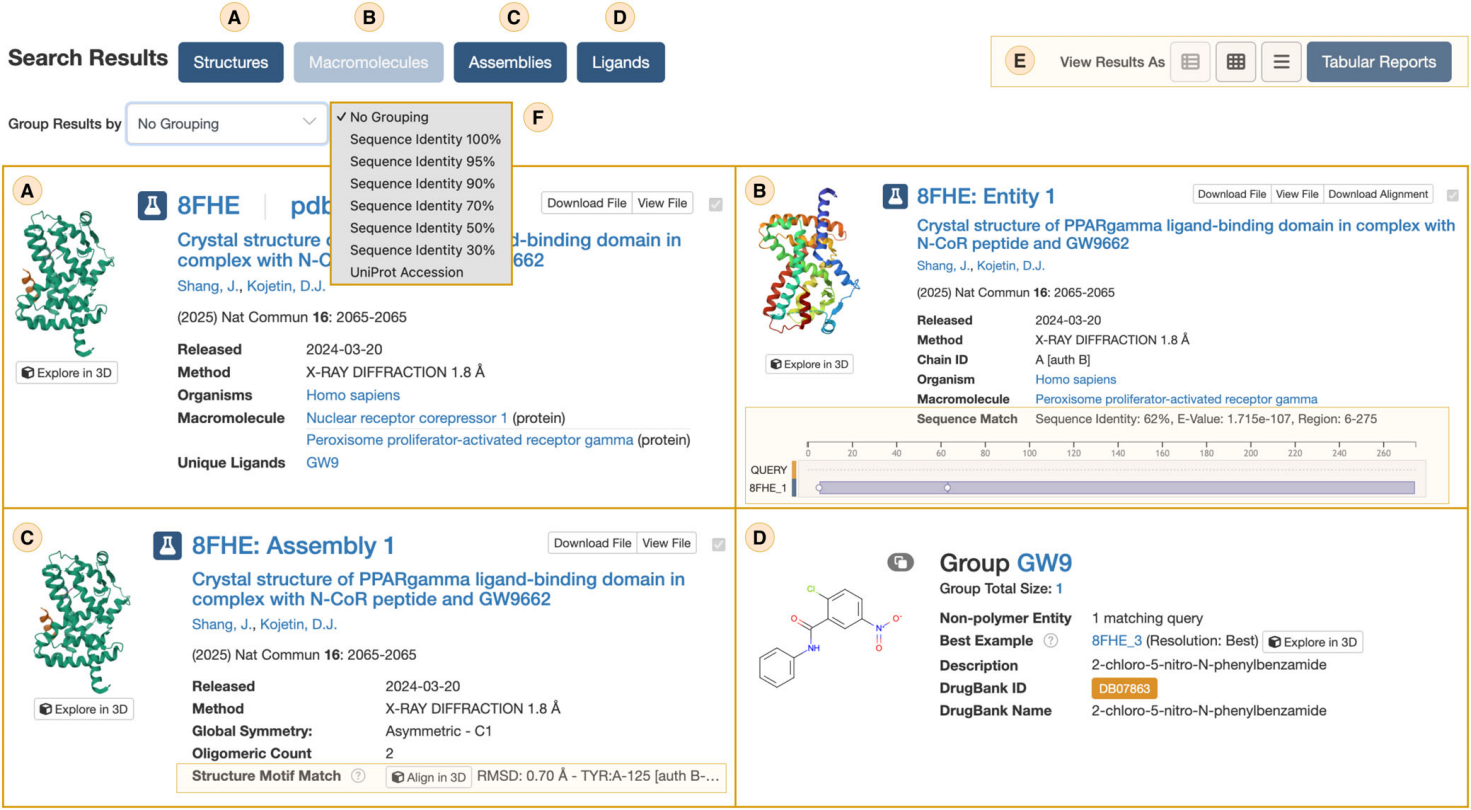
Multi‐level search results exploration in the redesigned Advanced Search interface. (a) Structures view showing individual PDB or CSM entries with entry‐level deposition metadata. (b) Macromolecules view presenting polymer entities for sequence‐level analysis, including sequence match statistics and alignments. (c) Assemblies view highlighting biologically relevant macromolecular assemblies with statistics and visualization links for 3D motif matches. (d) Ligands view enabling ligand‐centric exploration of small molecules, cofactors, metal ions, and other non‐polymeric components across structures. (e) Multiple presentation modes (Summary, Gallery, Compact, and Tabular Reports) allow flexible visualization of search hits, including customizable metadata display for each result. (f) Dynamic grouping controls applied during results exploration, allowing interactive aggregation and reorganization of result sets without modifying the original query.

The Structures view (Figure 5a) displays individual PDB or CSM entries and summarizes relevant entry‐level metadata, including release date, experimental method, macromolecular content, and associated ligands. This view is well suited for users interested in overall structural context or dataset‐level exploration.

The Macromolecules view (Figure 5b) focuses on polymer entities (proteins, DNA, or RNA) within each structure, enabling analysis at the sequence level. This view is particularly suited for queries involving sequence‐based properties, such as macromolecular names, organism taxonomy, enzyme classification, sequence similarity, and sequence motifs. It provides additional metadata and visualization tools, including sequence match statistics, embedded sequence alignment displays for sequence similarity searches, and direct links to pairwise structural alignment for chain‐level 3D similarity matches.

The Assemblies view (Figure 5c) highlights biologically relevant assemblies, which are functional macromolecular complexes formed by one or more polymer chains. By emphasizing functional complexes rather than individual chains, this view provides a biologically meaningful context for interpreting structural similarity and 3D motif search results. For 3D motif searches, each hit includes motif match statistics and a direct link to a 3D alignment visualization of the matched motif.

The Ligands view (Figure 5d), newly introduced in the redesigned interface, enables ligand‐centric exploration of non‐polymeric chemical components associated with search results, including small molecules, cofactors, metal ions, drug‐like molecules, and US FDA‐approved drugs. This view facilitates comparative analysis of ligand diversity, recurrence, and binding context across structures.

Within each view, search hits can be presented in Summary, Gallery, or Compact formats, or as customizable Tabular Reports that display results in a multi‐column, multi‐row table with optional metadata fields for each hit (Figure 5e).

An important modification to the redesigned interface is the relocation of result‐granularity selection from query construction to result exploration. In the legacy interface, users were required to choose in advance whether a query should return structures, polymer entities, or assemblies. The redesigned interface defers this decision until after execution of the search, allowing users to focus initially on defining what to search for rather than how results should be presented. To further reduce the burden of decision‐making, the most appropriate results view is automatically selected based on the query composition, while permitting users to switch to alternative views during exploration.

A related enhancement is the relocation of result grouping controls from query construction to results exploration (Figure 5f). Previously, PDB data consumers were required to specify grouping behavior, such as aggregation of polymer sequences by sequence similarity, during query building, introducing additional decisions before any results were visible. In the redesigned interface, grouping options are applied dynamically within the results view, allowing users to summarize result sets post hoc without modifying the underlying query.

### 1.6 Chemical search capabilities

The Chemical Search, available at rcsb.org/search/chemical, enables users to query chemical components found in PDB entries, including small molecules (such as cofactors, ions, modified residues, amino acids, and nucleotides) defined in the Chemical Component Dictionary (CCD) (Westbrook et al., 2015), and larger molecules (such as peptide‐like inhibitors and antibiotic molecules) defined in the Biologically Interesting Molecule Reference Dictionary (BIRD) (Dutta et al., 2013). The interface supports two complementary search tools:

- The Chemical Similarity search tool enables discovery of molecules with chemical structures similar to a specified query structure or formula, with multiple similarity modes that support both high‐precision chemical matching and exploratory searches for structurally related compounds; and
- The Chemical Attributes search tool allows users to filter molecules using curated metadata from the CCD and BIRD, such as chemical name or molecular weight. Filtering can also be based on external value‐added annotations, including PubChem (Kim et al., 2024) or DrugBank (Knox et al., 2023) IDs.

By supporting both structural similarity and attribute‐driven searches, Chemical Search enables fine‐grained exploration of small molecules and polymeric residues within the PDB archive and identification of functional ligands, cofactors, and peptide‐like molecules relevant to structural and functional studies.

### 1.7 Embedded chemical structure editor

**FIGURE 6:**
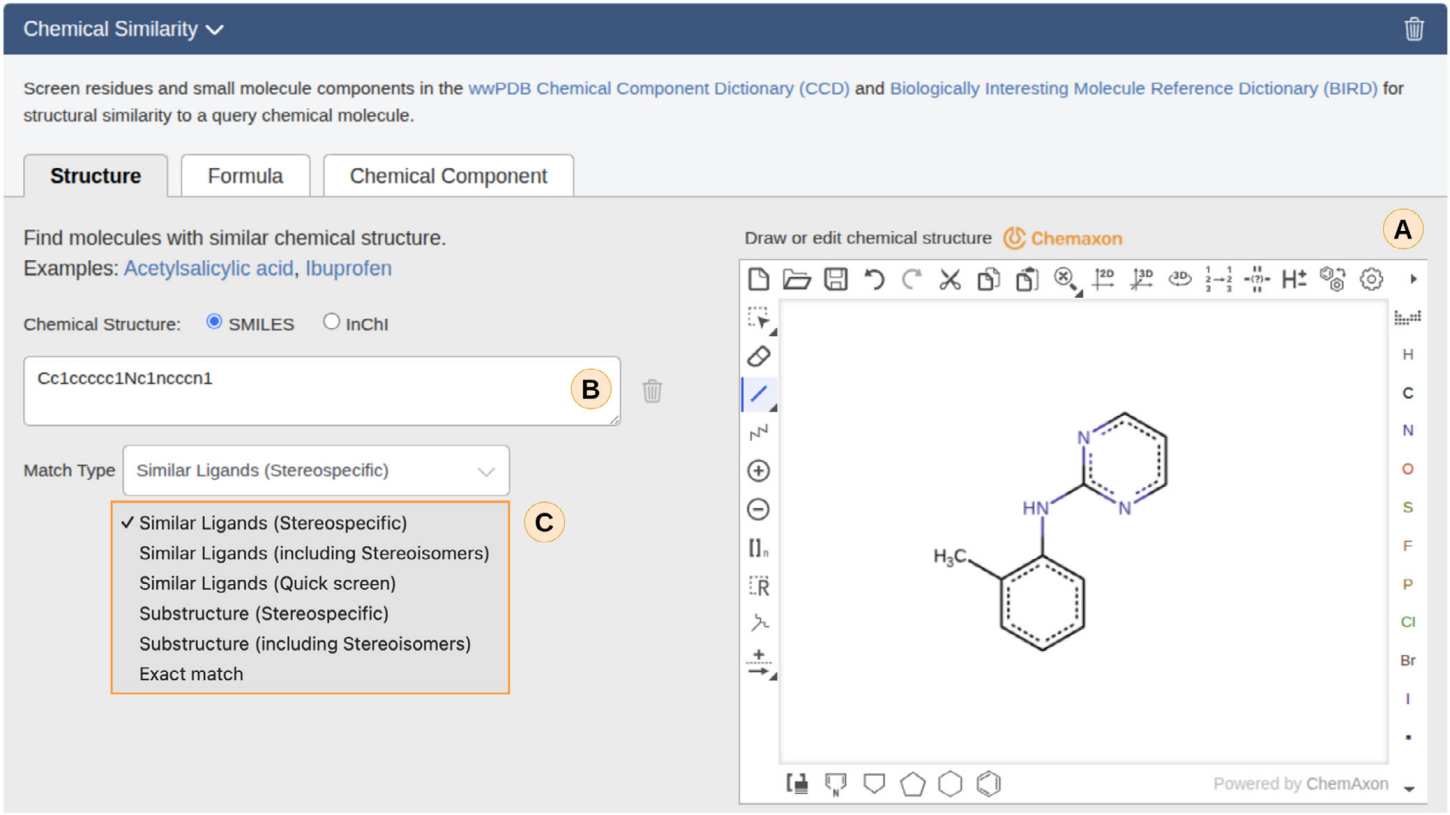
Chemical query construction in the redesigned Chemical Search interface. (a) Embedded MarvinJS chemical drawing editor enabling interactive creation and modification of small‐molecule structures. (b) Standard chemical identifiers (e.g., SMILES or InChI) encoding the drawn chemical structure. (c) Match type selection menu allowing users to control chemical comparison behavior during similarity or substructure searches. The example shown is the methyl‐bearing 2‐phenylaminopyrimidine scaffold: Starting with SMILES N(c1ccccc1)c1ncccn1, corresponding to the 2‐phenylaminopyrimidine core, a methyl group was added to the aniline ring ortho to the anilino nitrogen.

To support interactive chemical query construction, the redesigned Chemical Search interface integrates an embedded chemical drawing tool based on the Marvin JS web‐based editor (Marvin 22.11.1, ChemAxon; chemaxon.com) (Figure 6a, upper right). This editor enables interactive creation and modification of chemical structures, including specification of atom and bond types, stereochemistry, and ring systems, providing a flexible input mechanism for chemical similarity searches. Chemical structures created or edited within MarvinJS are automatically converted into standard chemical identifiers, such as SMILES or InChI, for downstream similarity searching. Users may select the preferred representation, with automatic interconversion handled by the interface.

The interface also supports direct textual input of chemical identifiers, including SMILES and InChI strings (Figure 6b). Entered identifiers are immediately rendered as molecular structures within the editor, allowing users to verify visually and, if necessary, further modify the molecule prior to executing the desired search. The Match Type dropdown includes options for measuring similarity that range from exact match, which requires near‐identical molecular graphs, to stereospecific and stereochemistry‐agnostic similarity or substructure searches, and a fast fingerprint‐based similarity screen for rapid retrieval of broadly related molecules (Figure 6c). This integration enables seamless definition of chemical queries within the Chemical Search interface, eliminating the need for external cheminformatics tools or manual format conversion.

## 2 DISCUSSION

### 2.1 Case study 1: 3D Motif search using the trypsin catalytic site template

To demonstrate the utility of template‐based 3D motif search driven by curated catalytic site definitions, we present a use case based on the trypsin catalytic site. Trypsin is a very well‐characterized serine protease, whose activity depends on a conserved set of residues that support peptide bond hydrolysis (Hedstrom, 2002). While the catalytic mechanism is often summarized by the canonical Ser‐His‐Asp triad, efficient catalysis requires a broader, spatially organized chemical environment that stabilizes reaction intermediates and governs substrate specificity.

In the redesigned Advanced Search interface, users can select the trypsin catalytic site directly from the M‐CSA library by entering *trypsin* as the molecule name in the template selector. The corresponding M‐CSA entry (M‐CSA 173) provides an expert‐curated definition of this functional site, mapped to a representative trypsin structure (PDB ID pdb_00001pq5), and includes six key residues: His:A 41 [auth 56], Asp:A 84 [auth 99] and Ser:A 180 [auth 195] that form the catalytic triad as well as Gln:A 177 [auth 192], Gly:A 178 [auth 193], and Asp:A 179 [auth 194] that define the specificity pocket that governs substrate recognition by favoring positively charged residues, that is, lysine or arginine. Together, these six amino acid residues form a spatially defined and functionally critical active‐site architecture required for tryptase activity. Upon selection, catalytic and supporting residues are automatically loaded as a 3D motif and visualized in the integrated Mol* viewer, allowing users to inspect both the canonical triad and the surrounding structural features that complete the active site geometry.

Execution of this search at the time of writing (June 2026) yielded 1552 biological assemblies. Each assembly contained at least one 3D motif matching the query with a root‐mean‐square deviation (RMSD) of 2.0 Å or less. A single assembly can contain multiple, repeated motif matches, leading to redundancy at the protein level. To obtain a non‐redundant set of motif occurrences across distinct proteins, we selected the single motif match with the lowest RMSD for each unique polymeric PDB entity. Such filtering yielded 1281 best‐matching motifs across distinct structures (Table S1). Those in turn correspond to 50 unique UniProt accession IDs. The EC classification of retrieved proteins is consistent with the use of a trypsin catalytic site. As expected, the most abundant subclass is trypsin itself (EC 3.4.21.4; 699 chains). Going beyond trypsin, results include multiple well‐represented subclasses of trypsin‐like serine proteases, which are distinguished by substrate specificity rather than catalytic mechanism. EC 3.4.21.36 (122 chains) and EC 3.4.21.73 (137 chains) correspond to regulatory and immune‐associated serine proteases, including complement and coagulation‐related enzymes. These proteins retain the conserved His‐Asp‐Ser catalytic core and oxyanion hole architecture but differ in surface loops that dictate substrate‐binding specificity and regulatory interactions. EC 3.4.21.6 (140 chains) and EC 3.4.21.22 (29 chains) represent chymotrypsin‐ and elastase‐like proteases, respectively. Although their substrate preferences differ from those of trypsin, their catalytic residues adopt nearly identical 3D arrangements, explaining their recovery by geometry‐driven motif matching. EC 3.4.21.59 (30 chains) and EC 3.4.21.109 (22 chains) include proteases functioning in more specialized physiological contexts (e.g., developmental or immune signaling), again reflecting conservation of catalytic site geometry despite functional diversification. Less frequently identified EC subclasses (e.g., EC 3.4.21.10, 3.4.21.38, 3.4.21.68, 3.4.21.118, and 3.4.21.122) appear with smaller counts, but all of these enzymes utilize the same fundamental serine protease reaction mechanism. Overall, the EC distribution demonstrates that the 3D motif search is driven by conservation of catalytic geometry, as opposed to sequence identity. All retrieved subclasses share the core features of the serine protease mechanism, including a conserved His‐Asp‐Ser catalytic triad, a glycine‐rich oxyanion hole that stabilizes the tetrahedral reaction intermediate, and surrounding residues that precisely position the nucleophile and the general base.

When the same search is performed with CSMs included, more than 300 additional structures are returned. Of these, 235 are classified as hydrolases, with their catalytic residues recovered within the 2.0 Å RMSD threshold, indicating that the conserved His‐Asp‐Ser geometry is present in the predicted structures. Notably, many of these matches correspond to proteins from model organisms for which the experimental‐structure‐only searching returned no hits. For example, 42 additional structures were recovered from Mus musculus, including multiple trypsin‐like serine proteases. Hits from other relevant organisms, including *Rattus norvegicus*, *Danio rerio*, and *Drosophila melanogaster*, were also identified when CSMs were included but were absent from the experimental‐structure‐only result set. These results demonstrate that incorporating CSMs augments rather than replaces experimentally determined structures, extending coverage into taxonomic spaces that are not yet represented by experimental structural data.

This case study illustrates the value of curated, multi‐residue catalytic site definitions for structure‐based discovery. By capturing both catalytic and supporting residues required for enzymatic function, M‐CSA templates provide biologically meaningful 3D motifs that can be reused directly in Advanced Search. Our approach embeds expert knowledge into the query construction process, enabling users to perform precise, function‐oriented structural searches without needing to manually define residue identities, numbering schemes, or catalytic geometry.

### 2.2 Case study 2: Ligand‐guided 3D motif search for Zn^2+^‐binding proteins

Zn^2+^‐binding proteins span a wide range of biological functions, including catalysis, regulation, and macromolecular recognition. Despite diversity in sequence and fold, Zn^2+^ binding is mediated by a small set of conserved three‐dimensional coordination geometries. In most cases, the metal ion is coordinated by three to four residues, typically histidine, cysteine, aspartate, or glutamate, arranged in a characteristic tetrahedral configuration (Vallee & Auld, 1990).

**FIGURE 7:**
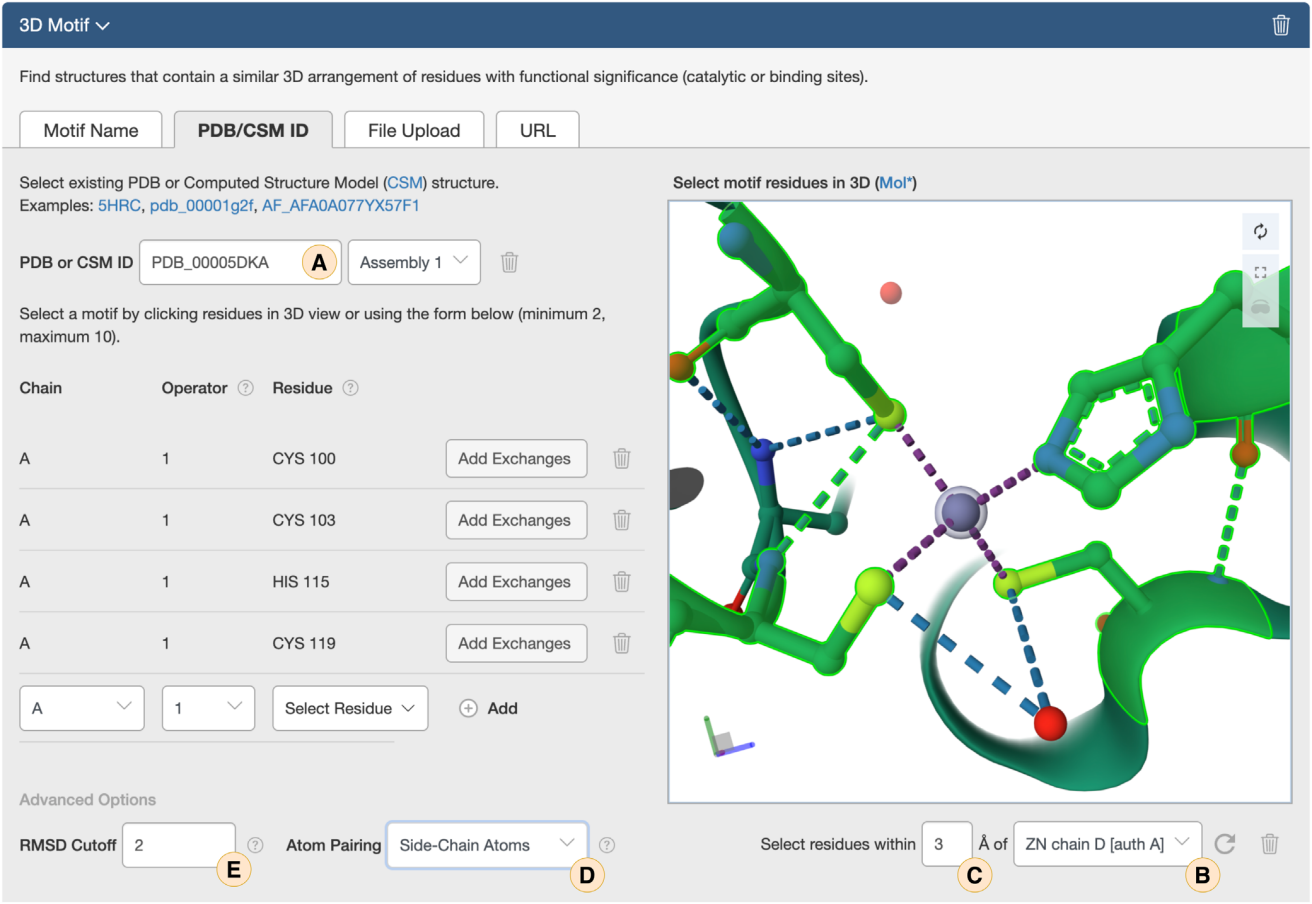
Ligand‐guided selection of a C2HC zinc‐finger motif from PDB ID pdb_00005dka (Bijlmakers et al., 2016) using the 3D Motif interface. (a) Structure selection from the PDB archive. (b) Ligand chosen from the dropdown menu. (c) Spatial radius control defining the distance for selecting interacting residues. (d) Motif comparison mode specifying which atoms are used for alignment and RMSD calculation. (e) RMSD cutoff control determining the required structural similarity for reported matches.

To demonstrate ligand‐guided residue selection, we used the C2HC zinc‐finger structure from PDB ID pdb_00005dka, in which the Zn^2+^ ion is coordinated by the canonical C2HC motif (two cysteines and one histidine plus a third cysteine). Using the Advanced Search interface, this structure was selected from the PDB archive (Figure 7a) and the Zn^2+^ ion was chosen directly from the ligand dropdown (Figure 7b, lower right). A shorter spatial radius of 3 Å was specified to capture direct coordination interactions, consistent with typical metal–ligand bond lengths (Figure 7c). This approach resulted in a minimal motif consisting solely of Zn^2+^‐coordinating residues. Motif comparison was performed using sidechain atom pairing (Figure 7d), restricting spatial matching to sidechain atoms, which is appropriate for metal‐binding sites wherein sidechain chemistry and orientation determine function. The search performed at the time of writing (June 2026) retrieved approximately 400 biological assemblies, each containing at least one motif with a root‐mean‐square deviation (RMSD) ≤ 2.0 Å relative to the query (Figure 7e). For downstream analysis, we focused on 326 PDB sequences corresponding to the best motif match (Table S2). Those in turn correspond to 141 unique UniProt accessions.

Most of the Zn^2+^‐binding proteins identified by this search correspond to E3 ubiquitin‐protein ligases (EC 2.3.2.x), including RNF125, RAD18, TRAF4/6, UBR1, ARIH1/2, RNF168, RNF8, and multiple RING‐family proteins. Although these proteins vary widely in sequence and biological context, they consistently share a C2HC Zn^2+^‐binding motif that stabilizes zinc‐finger domains involved in protein–protein interactions (not catalysis). Additional hits include histone acetyltransferases from the MYST and p300/CBP families (EC 2.3.1.48), where Zn^2+^ motifs occur in regulatory domains adjacent to the catalytic center, and a smaller set of hydrolases, including RNA‐processing enzymes and cardiolipin hydrolases, where Zn^2+^ contributes to substrate recognition and positioning of catalytic residues. The search also recovered non‐enzymatic proteins such as transcription factors, ribosomal proteins, viral proteins, and transposases, indicating that results are driven by ligand coordination geometry rather than enzyme class or fold type.

These results show that the 3D Motif search effectively identifies proteins unified by ligand environment rather than by sequence similarity, protein fold type, or enzyme class. This outcome illustrates the utility of ligand‐centered 3D motif searches for exploring functional convergence and mechanistic diversity in macromolecular structures. Importantly, construction of this 3D motif query in the redesigned Advanced Search interface required only a few guided interactions and relied on basic biochemical knowledge of metal coordination rather than residue numbering or chain identifiers. Despite the need for minimal input, the search produced a focused and biologically coherent result set, demonstrating that the new interface dramatically lowers the barrier to constructing expressive structural queries and enables users to leverage fundamental biochemical and chemical principles rather than detailed familiarity with structural identifiers or database‐specific metadata.

### 2.3 Case study 3: Chemical scaffold search for kinase‐inhibitor using the chemical sketcher

Small‐molecule drug discovery frequently centers on a shared chemical substructure, or scaffold, that recurs across otherwise dissimilar inhibitors and confers a common mode of target engagement. For example, the 2‐phenylaminopyrimidine scaffold, composed of a pyrimidine ring linked through an exocyclic amine to a substituted aniline, constitutes the ATP‐site hinge‐binding core of numerous protein kinase inhibitors (Pimentel et al., 2020). Within this chemotype, addition of a single methyl group on the aniline ring, ortho to the anilino nitrogen (the “flag methyl”), shifts selectivity toward tyrosine kinases such as Bcr‐Abl and PDGFR, in part by sterically restricting the accessible inhibitor conformations (Longo, 2017). A researcher studying this fragment cannot enumerate the corresponding chemical components by identifier or keyword, because matching molecules are deposited under unrelated, non‐mnemonic ligand codes; therefore, graph‐based chemical search is required.

To demonstrate substructure‐driven chemical discovery, we constructed a query for this methyl‐bearing 2‐phenylaminopyrimidine scaffold directly within the Chemical Search interface. Using the embedded Marvin JS editor, we drew the fragment with the methyl substituent positioned ortho to the anilino nitrogen (Figure 6). We then selected the Substructure match type from the Match Type menu, which retrieves CCD and BIRD chemical components that contain the query fragment as a substructure within a larger molecular graph.

**FIGURE 8:**
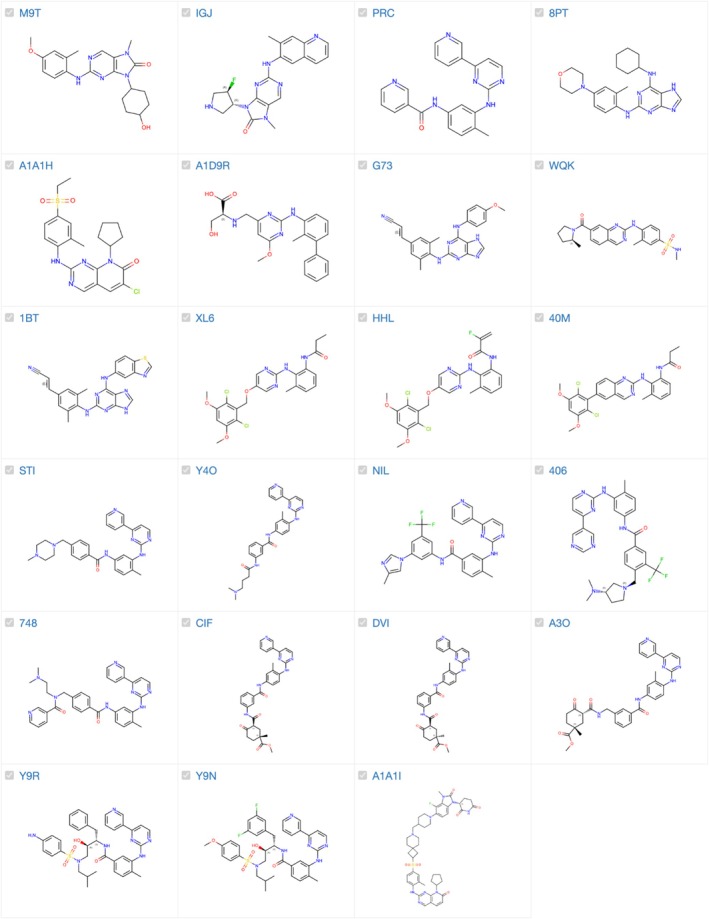
Chemical Search results for the methyl‐bearing 2‐phenylaminopyrimidine scaffold, shown in the Ligands view. A substructure search returned 23 chemical components spanning a broad range of biological targets and therapeutic modalities, including the FDA‐approved Bcr‐Abl inhibitors imatinib (STI) and nilotinib (NIL), CDK2 and FGFR inhibitors (WQK and 40 M), and a covalent inhibitor (HHL).

At the time of writing (June 2026), this chemical search returned 23 chemical components spanning a broad range of biological targets and therapeutic modalities (Figure 8). The results included the US FDA‐approved drugs imatinib (STI) (Iqbal and Iqbal 2014) and nilotinib (NIL) (Blay and von Mehren 2011) alongside several 2‐anilinopyrimidine benzamide analogs (e.g., PRC, Y4O, 748) representing type‐II Bcr‐Abl kinase inhibitors. The returned set also included inhibitors targeting CDK2 (e.g., A1A1H, WQK), the receptor tyrosine kinase FGFR (e.g., XL6, 40M), and multiple purine‐based kinase inhibitors (e.g., M9T, 8PT). Beyond conventional reversible inhibitors, the results captured a covalently bound compound bearing a 2‐fluoroacrylamide cysteine‐targeting warhead (HHL).

This case study illustrates that substructure search within the redesigned Chemical Search interface retrieves molecules unified by a shared chemical recognition element rather than by ligand naming or keyword‐based annotation. As with the geometry‐driven 3D motif searches in Case Studies 1 and 2, the result set is organized by an underlying structural principle, in this case a two‐dimensional chemical substructure that remains coherent across diverse targets and molecular architectures. Constructing the query only required a SMILES string, drawing a small fragment in the embedded editor, and selecting a match type, without recourse to external cheminformatics tools, ligand identifiers, or format conversion. This demonstrates how the new interface lowers the barrier to chemically driven exploration of the archive.

## AUTHOR CONTRIBUTIONS

**Yana Rose:** Conceptualization; writing – review and editing; writing – original draft; project administration; supervision; visualization; software. **Stephen K. Burley:** Conceptualization; writing – review and editing; supervision; funding acquisition. **Charmi Bhikadiya:** Writing – review and editing; software. **Sebastian Bittrich:** Conceptualization; writing – review and editing. **Ronald Brown:** Software; writing – review and editing. **Jose M. Duarte:** Conceptualization; writing – review and editing; supervision. **Joan Segura:** Conceptualization; writing – review and editing. **Maria Voigt:** Conceptualization; software; writing – review and editing. **Rachel Kramer Green:** Conceptualization; writing – review and editing.

## Supporting information


**TABLE S1.** Distribution of Enzyme Commission (EC) subclasses among best‐matching 3D motif hits identified by 3D Motif search using a trypsin catalytic site template. The table was generated using a Python script that queries the 3D Motif search API; the script is provided as a publicly available example in the RCSB PDB training resources repository: trypsin‐catalytic‐site.py.

**TABLE S2.** Distribution of functional classes and Enzyme Commission (EC) annotations among PDB sequences corresponding to the best‐matching Zn^2+^‐binding motifs. The table was generated using a Python script that queries the 3D Motif search API; the script is provided as a publicly available example in the RCSB PDB training resources repository: C2HC‐zinc‐binding‐proteins.py.

## Data Availability

The data that supports the findings of this study are available in the supplementary material of this article.
