# Supplementary material for: RCSB protein data Bank: Next‐generation advanced search for exploration of experimental structures and computed structure models
Source: Protein Sci. 2026 Jul 28;35(8):e70731. doi: 10.1002/pro.70731 (PMC13410952; doi:10.1002/pro.70731)
Supplement: Supplementary file 1 — TABLE S1. Distribution of Enzyme Commission (EC) subclasses among best‐matching 3D motif hits identified by 3D Motif search using a trypsin catalytic site template. The table was generated using a Python script that queries the 3D Motif search API; the script is provided as a publicly available example in the RCSB PDB training resources repository: trypsin‐catalytic‐site.py. [file PRO-35-e70731-s001.pdf]

PDB ID	Entity ID	Chain ID(s)	UniProt ID(s)	Protein(s)	EC Class	EC Number(s)	RMSD
pdb_00001pq7	1	A	P35049	Trypsin	Hydrolase	3.4.21.4	0.09
pdb_00001xvm	1	A	P35049	Trypsin	Hydrolase	3.4.21.4	0.12
pdb_00001xvo	1	A	P35049	trypsin	Hydrolase	3.4.21.4	0.12
pdb_00001gdu	1	A	P35049	TRYPSIN	Hydrolase	3.4.21.4	0.13
pdb_00002g51	1	A	P35049	Trypsin	Hydrolase	3.4.21.4	0.13
pdb_00001pq8	1	A	P35049	Trypsin	Hydrolase	3.4.21.4	0.15
pdb_00002g52	1	A	P35049	Trypsin	Hydrolase	3.4.21.4	0.15
pdb_00001fn8	1	A	P35049	TRYPSIN	Hydrolase	3.4.21.4	0.17
pdb_00001gdq	1	A	P35049	TRYPSIN	Hydrolase	3.4.21.4	0.17
pdb_00002agg	1	A	P00760	Cationic trypsin	Hydrolase	3.4.21.4	0.17
pdb_00005dj7	1	A	Q54137	Trypsin			0.17
pdb_00006yiy	1	A	P00760	Cationic trypsin	Hydrolase	3.4.21.4	0.17
pdb_00001fy5	1	A	P35049	TRYPSIN	Hydrolase	3.4.21.4	0.18
pdb_00001fy4	1	A	P35049	TRYPSIN	Hydrolase	3.4.21.4	0.19
pdb_00001gdn	1	A	P35049	TRYPSIN	Hydrolase	3.4.21.4	0.19
pdb_00001ppz	1	A	P35049	Trypsin	Hydrolase	3.4.21.4	0.19
pdb_00002age	1	A	P00760	Cationic trypsin	Hydrolase	3.4.21.4	0.19
pdb_00002agi	1	A	P00760	beta-trypsin	Hydrolase	3.4.21.4	0.2
pdb_00005moq	1	A	P00760	Cationic trypsin	Hydrolase	3.4.21.4	0.2
pdb_00005xw1	2	A,B	P00761	Trypsin	Hydrolase	3.4.21.4	0.2
pdb_00005xwa	2	A,B	P00761	Trypsin	Hydrolase	3.4.21.4	0.2
pdb_00004m7g	1	A	P24664	Trypsin-like prote	Hydrolase	3.4.21.4	0.21
pdb_00005mnx	1	A	P00760	Cationic trypsin	Hydrolase	3.4.21.4	0.21
pdb_00005mo0	1	A	P00760	Cationic trypsin	Hydrolase	3.4.21.4	0.21
pdb_00005mon	1	A	P00760	Cationic trypsin	Hydrolase	3.4.21.4	0.21
pdb_00005mor	1	A	P00760	Cationic trypsin	Hydrolase	3.4.21.4	0.21
pdb_00005xw9	2	A,B	P00761	Trypsin	Hydrolase	3.4.21.4	0.21
pdb_00005xwj	1	A	P00761	Trypsin	Hydrolase	3.4.21.4	0.21
pdb_00007ahv	4	D	P00740	Coagulation facto	Hydrolase	3.4.21.22	0.21
pdb_00002f9n	1	C	Q15661	alpha I tryptase	Hydrolase	3.4.21.59	0.22
pdb_00005mna	1	A	P00760	Cationic trypsin	Hydrolase	3.4.21.4	0.22
pdb_00005mnb	1	A	P00760	Cationic trypsin	Hydrolase	3.4.21.4	0.22
pdb_00005moo	1	A	P00760	Cationic trypsin	Hydrolase	3.4.21.4	0.22
pdb_00005xw8	2	A,B	P00761	Trypsin	Hydrolase	3.4.21.4	0.22
pdb_00001w12	1	A	P00749	UROKINASE-TYPE	Hydrolase	3.4.21.73	0.23
pdb_00004mnv	1	A	P00749	Urokinase-type pl	Hydrolase	3.4.21.73	0.23
pdb_00005mny	1	A	P00760	Cationic trypsin	Hydrolase	3.4.21.4	0.23
pdb_00006b6q	1	A	P00760	Cationic trypsin	Hydrolase	3.4.21.4	0.23

PDB ID	Entity ID	Chain ID(s)	UniProt ID(s)	Protein(s)	EC Class	EC Number(s)	RMSD
pdb_00006sux	1	A	P00760	Cationic trypsin	Hydrolase	3.4.21.4	0.23
pdb_00006sv0	1	A	P00760	Cationic trypsin	Hydrolase	3.4.21.4	0.23
pdb_00006sv6	1	A	P00760	Cationic trypsin	Hydrolase	3.4.21.4	0.23
pdb_00006sv8	1	A	P00760	Cationic trypsin	Hydrolase	3.4.21.4	0.23
pdb_00007xyd	2	D	O15393	Transmembrane p	Hydrolase	3.4.21.122	0.23
pdb_00002f3c	1	A	P00760	Cationic trypsin	Hydrolase	3.4.21.4	0.24
pdb_00002fs9	1	D	P20231	Tryptase beta-2	Hydrolase	3.4.21.59	0.24
pdb_00005mo1	1	A	P00760	Cationic trypsin	Hydrolase	3.4.21.4	0.24
pdb_00005wxs	1	A	P00749	Urokinase-type pl	Hydrolase	3.4.21.73	0.24
pdb_00006eat	1	A	P00760	Cationic trypsin	Hydrolase	3.4.21.4	0.24
pdb_00006eau	1	A	P00760	Cationic trypsin	Hydrolase	3.4.21.4	0.24
pdb_00006eaw	1	A	P00760	Cationic trypsin	Hydrolase	3.4.21.4	0.24
pdb_00006svg	1	A	P00760	Cationic trypsin	Hydrolase	3.4.21.4	0.24
pdb_00006svi	1	A	P00760	Cationic trypsin	Hydrolase	3.4.21.4	0.24
pdb_00006svj	1	A	P00760	Cationic trypsin	Hydrolase	3.4.21.4	0.24
pdb_00006svv	1	A	P00760	Cationic trypsin	Hydrolase	3.4.21.4	0.24
pdb_00006svx	1	A	P00760	Cationic trypsin	Hydrolase	3.4.21.4	0.24
pdb_00006sw0	1	A	P00760	Cationic trypsin	Hydrolase	3.4.21.4	0.24
pdb_00009i24	1	A	P00742	Activated factor X	Hydrolase	3.4.21.6	0.24
pdb_00001h9h	1	A	P00761	TRYPSIN	Hydrolase	3.4.21.4	0.25
pdb_00002cmy	1	A	P00760	CATIONIC TRYPSI	Hydrolase	3.4.21.4	0.25
pdb_00002g8t	1	A	P00760	Cationic trypsin	Hydrolase	3.4.21.4	0.25
pdb_00003a7v	1	A	P00760	Cationic trypsin	Hydrolase	3.4.21.4	0.25
pdb_00003gy2	1	A	P00760	Cationic trypsin	Hydrolase	3.4.21.4	0.25
pdb_00004doq	1	E	P00761	Trypsin	Hydrolase	3.4.21.4	0.25
pdb_00004i8k	1	A	P00760	Cationic trypsin	Hydrolase	3.4.21.4	0.25
pdb_00004u2w	2	B	P00760	Cationic trypsin	Hydrolase	3.4.21.4	0.25
pdb_00005jbb	2	B	P00740	Coagulation facto	Hydrolase	3.4.21.22	0.25
pdb_00005k7r	1	A	P00760	Cationic trypsin	Hydrolase	3.4.21.4	0.25
pdb_00005mne	1	A	P00760	Cationic trypsin	Hydrolase	3.4.21.4	0.25
pdb_00006b6p	1	A	P00760	Cationic trypsin	Hydrolase	3.4.21.4	0.25
pdb_00006sv9	1	A	P00760	Cationic trypsin	Hydrolase	3.4.21.4	0.25
pdb_00006svb	1	A	P00760	Cationic trypsin	Hydrolase	3.4.21.4	0.25
pdb_00006svd	1	A	P00760	Cationic trypsin	Hydrolase	3.4.21.4	0.25
pdb_00006svn	1	A	P00760	Cationic trypsin	Hydrolase	3.4.21.4	0.25
pdb_00006svr	1	A	P00760	Cationic trypsin	Hydrolase	3.4.21.4	0.25
pdb_00006svu	1	A	P00760	Cationic trypsin	Hydrolase	3.4.21.4	0.25
pdb_00006svw	1	A	P00760	Cationic trypsin	Hydrolase	3.4.21.4	0.25

PDB ID	Entity ID	Chain ID(s)	UniProt ID(s)	Protein(s)	EC Class	EC Number(s)	RMSD
pdb_00006svz	1	A	P00760	Cationic trypsin	Hydrolase	3.4.21.4	0.25
pdb_00006yit	1	A	P00760	Cationic trypsin	Hydrolase	3.4.21.4	0.25
pdb_00006yza	1	A	P00760	Cationic trypsin	Hydrolase	3.4.21.4	0.25
pdb_00008kdu	1	A	P00760	Cationic trypsin	Hydrolase	3.4.21.4	0.25
pdb_00001pqa	1	A	P35049	Trypsin	Hydrolase	3.4.21.4	0.26
pdb_00001tio	1	A	P00760	PROTEIN (BETA-T	Hydrolase	3.4.21.4	0.26
pdb_00001w10	1	A	P00749	UROKINASE-TYPE	Hydrolase	3.4.21.73	0.26
pdb_00001z8g	1	A	P05981	Serine protease h	Hydrolase	3.4.21.106	0.26
pdb_00002fxr	1	A	P20231	Tryptase beta-2	Hydrolase	3.4.21.59	0.26
pdb_00002g5n	1	A	P00760	Cationic trypsin	Hydrolase	3.4.21.4	0.26
pdb_00002plx	1	A	P00760	Cationic trypsin	Hydrolase	3.4.21.4	0.26
pdb_00003ljj	1	A	P00760	Cationic trypsin	Hydrolase	3.4.21.4	0.26
pdb_00005jb8	2	B	P00740	Coagulation facto	Hydrolase	3.4.21.22	0.26
pdb_00005mng	1	A	P00760	Cationic trypsin	Hydrolase	3.4.21.4	0.26
pdb_00005mnk	1	A	P00760	Cationic trypsin	Hydrolase	3.4.21.4	0.26
pdb_00005mop	1	A	P00760	Cationic trypsin	Hydrolase	3.4.21.4	0.26
pdb_00005t3h	1	A	P00760	Cationic trypsin	Hydrolase	3.4.21.4	0.26
pdb_00008zvx	1	A		snFPITE-n2 A cha			0.26
pdb_00008zvx	2	B		snFPITE-n2 B cha			1.14
pdb_00009aw1	1	A	P00760	Cationic trypsin	Hydrolase	3.4.21.4	0.26
pdb_00009aw4	1	A	P00760	Cationic trypsin	Hydrolase	3.4.21.4	0.26
pdb_00001g9i	1	A	P00760	TRYPSINOGEN, C	Hydrolase	3.4.21.4	0.27
pdb_00001hay	1	A	P00772	ELASTASE 1	Hydrolase	3.4.21.36	0.27
pdb_00001jrs	1	A	P00760	TRYPSIN	Hydrolase	3.4.21.4	0.27
pdb_00001jrt	1	A	P00760	TRYPSIN	Hydrolase	3.4.21.4	0.27
pdb_00001w14	1	A	P00749	UROKINASE-TYPE	Hydrolase	3.4.21.73	0.27
pdb_00002fpz	1	A	P20231	Tryptase beta-2	Hydrolase	3.4.21.59	0.27
pdb_00002fx4	1	A	P00760	trypsin	Hydrolase	3.4.21.4	0.27
pdb_00002zq2	1	A	P00760	Cationic trypsin	Hydrolase	3.4.21.4	0.27
pdb_00003a80	1	A	P00760	Cationic trypsin	Hydrolase	3.4.21.4	0.27
pdb_00004i8j	1	A	P00760	Cationic trypsin	Hydrolase	3.4.21.4	0.27
pdb_00004i8l	1	A	P00760	Cationic trypsin	Hydrolase	3.4.21.4	0.27
pdb_00005mnf	1	A	P00760	Cationic trypsin	Hydrolase	3.4.21.4	0.27
pdb_00005mo2	1	A	P00760	Cationic trypsin	Hydrolase	3.4.21.4	0.27
pdb_00006mrq	1	A	P00760	Cationic trypsin	Hydrolase	3.4.21.4	0.27
pdb_00006rfk	2	B	P00740	Coagulation facto	Hydrolase	3.4.21.22	0.27
pdb_00009avx	1	A	P00760	Cationic trypsin	Hydrolase	3.4.21.4	0.27
pdb_00009aw8	1	A	P00760	Cationic trypsin	Hydrolase	3.4.21.4	0.27

PDB ID	Entity ID	Chain ID(s)	UniProt ID(s)	Protein(s)	EC Class	EC Number(s)	RMSD
pdb_00009awa	1	A	P00760	Cationic trypsin	Hydrolase	3.4.21.4	0.27
pdb_00009bom	1	A	P00760	Cationic trypsin	Hydrolase	3.4.21.4	0.27
pdb_00001gwa	1	A	P00772	ELASTASE 1	Hydrolase	3.4.21.36	0.28
pdb_00001hb0	1	A	P00772	ELASTASE 1	Hydrolase	3.4.21.36	0.28
pdb_00002fs8	1	A	P20231	Tryptase beta-2	Hydrolase	3.4.21.59	0.28
pdb_00002gdd	1	A	P20231	Tryptase beta-2	Hydrolase	3.4.21.59	0.28
pdb_00002tio	1	A	P00760	PROTEIN (BETA-T	Hydrolase	3.4.21.4	0.28
pdb_00002uuy	1	A	P00760	CATIONIC TRYPSI	Hydrolase	3.4.21.4	0.28
pdb_00003e0p	1	A	Q16651	Prostasin	Hydrolase	3.4.21	0.28
pdb_00003rdz	1	A	P00760	Cationic trypsin	Hydrolase	3.4.21.4	0.28
pdb_00004dgj	1	A	P98073	Enteropeptidase c	Hydrolase	3.4.21.9	0.28
pdb_00004i8h	1	A	P00760	Cationic trypsin	Hydrolase	3.4.21.4	0.28
pdb_00004ncy	1	A	P00760	Cationic trypsin	Hydrolase	3.4.21.4	0.28
pdb_00005kwm	1	A	Q54137	Trypsin			0.28
pdb_00006b6o	1	A	P00760	Cationic trypsin	Hydrolase	3.4.21.4	0.28
pdb_00006eax	1	A	P00760	Cationic trypsin	Hydrolase	3.4.21.4	0.28
pdb_00007meq	1	A	O15393	Transmembrane p	Hydrolase	3.4.21.122	0.28
pdb_00007y0e	2	D	O15393	Transmembrane p	Hydrolase	3.4.21.122	0.28
pdb_00009avy	1	A	P00760	Cationic trypsin	Hydrolase	3.4.21.4	0.28
pdb_00009aw0	1	A	P00760	Cationic trypsin	Hydrolase	3.4.21.4	0.28
pdb_00009aw2	1	A	P00760	Cationic trypsin	Hydrolase	3.4.21.4	0.28
pdb_00009aw9	1	A	P00760	Cationic trypsin	Hydrolase	3.4.21.4	0.28
pdb_00009awc	1	A	P00760	Cationic trypsin	Hydrolase	3.4.21.4	0.28
pdb_00009qfu	1	C	P20231	Tryptase beta-2	Hydrolase	3.4.21.59	0.28
pdb_00001esb	1	A	P00772	PORCINE PANCRI	Hydrolase	3.4.21.36	0.29
pdb_00001j15	1	A	P00763	Trypsin II, anionic	Hydrolase	3.4.21.4	0.29
pdb_00001j16	1	A	P00763	Trypsin II, anionic	Hydrolase	3.4.21.4	0.29
pdb_00001l1g	1	A	P00772	ELASTASE 1	Hydrolase	3.4.21.36	0.29
pdb_00001y3w	1	A	P00760	Trypsinogen, catic	Hydrolase	3.4.21.4	0.29
pdb_00002fof	1	A	P00772	elastase-1	Hydrolase	3.4.21.36	0.29
pdb_00002stb	1	A	P35031	PROTEIN (TRYPSI	Hydrolase	3.4.21.4	0.29
pdb_00002zq1	1	A	P00760	Cationic trypsin	Hydrolase	3.4.21.4	0.29
pdb_00003a85	1	A	P00760	Cationic trypsin	Hydrolase	3.4.21.4	0.29
pdb_00003fp6	1	A	P00763	Anionic trypsin-2	Hydrolase	3.4.21.4	0.29
pdb_00003mfj	1	A	P00760	Cationic trypsin	Hydrolase	3.4.21.4	0.29
pdb_00003mi4	1	A	P00760	Cationic trypsin	Hydrolase	3.4.21.4	0.29
pdb_00004gux	1	B	P00760	Cationic trypsin	Hydrolase	3.4.21.4	0.29
pdb_00004i8g	1	A	P00760	Cationic trypsin	Hydrolase	3.4.21.4	0.29

PDB ID	Entity ID	Chain ID(s)	UniProt ID(s)	Protein(s)	EC Class	EC Number(s)	RMSD
pdb_00005mnc	1	A	P00760	Cationic trypsin	Hydrolase	3.4.21.4	0.29
pdb_00005mnz	1	A	P00760	Cationic trypsin	Hydrolase	3.4.21.4	0.29
pdb_00009awo	1	A	P00760	Cationic trypsin	Hydrolase	3.4.21.4	0.29
pdb_00001esa	1	A	P00772	PORCINE PANCRI	Hydrolase	3.4.21.36	0.3
pdb_00001h9i	1	A	P00761	TRYPSIN	Hydrolase	3.4.21.4	0.3
pdb_00001tyn	1	A	P00760	BETA-TRYPSIN	Hydrolase	3.4.21.4	0.3
pdb_00001uo6	1	A	P00772	ELASTASE 1	Hydrolase	3.4.21.36	0.3
pdb_00002a7c	1	A	P00772	Elastase 1	Hydrolase	3.4.21.36	0.3
pdb_00002bdb	1	A	P00772	Elastase-1	Hydrolase	3.4.21.36	0.3
pdb_00002ftm	1	A	P00760	Cationic trypsin	Hydrolase	3.4.21.4	0.3
pdb_00002oqu	1	A	P00772	Elastase-1	Hydrolase	3.4.21.36	0.3
pdb_00002sta	1	A	P35031	PROTEIN (TRYPSI	Hydrolase	3.4.21.4	0.3
pdb_00003a7x	1	A	P00760	Cationic trypsin	Hydrolase	3.4.21.4	0.3
pdb_00003est	1	A	P00772	PORCINE PANCRI	Hydrolase	3.4.21.36	0.3
pdb_00004abi	1	A	P00760	CATIONIC TRYPSI	Hydrolase	3.4.21.4	0.3
pdb_00005wi6	1	B	Q15661	Tryptase alpha/be	Hydrolase	3.4.21.59	0.3
pdb_00006bfp	1	A	P00760	Cationic trypsin	Hydrolase	3.4.21.4	0.3
pdb_00006zov	1	A	P98073	Enteropeptidase	Hydrolase	3.4.21.9	0.3
pdb_00008uo7	1	A	P00760	Cationic trypsin	Hydrolase	3.4.21.4	0.3
pdb_00008utl	1	A	P00760	Cationic trypsin	Hydrolase	3.4.21.4	0.3
pdb_00001bth	2	D	P00734	THROMBIN	Hydrolase	3.4.21.5	0.31
pdb_00001btx	1	A	P00760	BETA-TRYPSIN	Hydrolase	3.4.21.4	0.31
pdb_00001btz	1	A	P00760	BETA-TRYPSIN	Hydrolase	3.4.21.4	0.31
pdb_00001co7	1	A	P00763	TRYPSIN II	Hydrolase	3.4.21.4	0.31
pdb_00001f2s	1	A	P00760	TRYPSIN	Hydrolase	3.4.21.4	0.31
pdb_00001l0z	1	A	P00772	ELASTASE 1	Hydrolase	3.4.21.36	0.31
pdb_00001ldt	1	A	P00761	TRYPSIN	Hydrolase	3.4.21.4	0.31
pdb_00001qcp	1	A	P00760	PROTEIN (BETA-T	Hydrolase	3.4.21.4	0.31
pdb_00001qnj	1	A	P00772	ELASTASE	Hydrolase	3.4.21.36	0.31
pdb_00001s6h	1	A	P00761	Trypsin	Hydrolase	3.4.21.4	0.31
pdb_00001sfi	1	A	P00760	TRYPSIN	Hydrolase	3.4.21.4	0.31
pdb_00001smf	1	A	P00760	TRYPSIN	Hydrolase	3.4.21.4	0.31
pdb_00002bda	2	B	P00772	Chymotrypsin-like	Hydrolase	3.4.21.36	0.31
pdb_00002fog	1	A	P00772	elastase-1	Hydrolase	3.4.21.36	0.31
pdb_00003e3t	1	A	P00772	Elastase-1	Hydrolase	3.4.21.36	0.31
pdb_00003gy5	1	A	P00760	Cationic trypsin	Hydrolase	3.4.21.4	0.31
pdb_00004kts	1	A	P00760	Cationic trypsin	Hydrolase	3.4.21.4	0.31
pdb_00004xoj	1	A	P00760	Cationic trypsin	Hydrolase	3.4.21.4	0.31

PDB ID	Entity ID	Chain ID(s)	UniProt ID(s)	Protein(s)	EC Class	EC Number(s)	RMSD
pdb_00005f6m	1	A	P00760	Cationic trypsin	Hydrolase	3.4.21.4	0.31
pdb_00006bvh	1	A	P00760	Cationic trypsin	Hydrolase	3.4.21.4	0.31
pdb_00006dzf	1	A	P00760	Cationic trypsin	Hydrolase	3.4.21.4	0.31
pdb_00006q8s	1	A	P00772	Chymotrypsin-like	Hydrolase	3.4.21.36	0.31
pdb_00007fag	1	A	P00772	Chymotrypsin-like	Hydrolase	3.4.21.36	0.31
pdb_00008b1y	1	A	P00772	Chymotrypsin-like	Hydrolase	3.4.21.36	0.31
pdb_00009pct	1	A	P00761	Trypsin	Hydrolase	3.4.21.4	0.31
pdb_00001bty	1	A	P00760	BETA-TRYPSIN	Hydrolase	3.4.21.4	0.32
pdb_00001ejm	1	A	P00760	BETA-TRYPSIN	Hydrolase	3.4.21.4	0.32
pdb_00001ekb	2	B	P98072	ENTEROPEPTIDA	Hydrolase	3.4.21.9	0.32
pdb_00001j14	1	A	P00763	trypsin II, anionic	Hydrolase	3.4.21.4	0.32
pdb_00001lka	1	A	P00772	Elastase 1	Hydrolase	3.4.21.36	0.32
pdb_00001lkb	1	A	P00772	Elastase 1	Hydrolase	3.4.21.36	0.32
pdb_00001n6x	1	A	P00760	Trypsinogen, catio	Hydrolase	3.4.21.4	0.32
pdb_00001n6y	1	A	P00760	Trypsinogen, catio	Hydrolase	3.4.21.4	0.32
pdb_00001ppe	1	A	P00760	TRYPSIN	Hydrolase	3.4.21.4	0.32
pdb_00001sbw	1	A	P00760	PROTEIN (BETA-T	Hydrolase	3.4.21.4	0.32
pdb_00001uvo	1	A	P00772	ELASTASE 1	Hydrolase	3.4.21.36	0.32
pdb_00001v2j	1	A	P00760	Trypsin	Hydrolase	3.4.21.4	0.32
pdb_00001v2v	1	A	P00760	Trypsin	Hydrolase	3.4.21.4	0.32
pdb_00002bd3	2	B	P00772	Chymotrypsin-like	Hydrolase	3.4.21.36	0.32
pdb_00002bdc	1	A	P00772	Elastase-1	Hydrolase	3.4.21.36	0.32
pdb_00002blo	1	A	P00772	ELASTASE 1	Hydrolase	3.4.21.36	0.32
pdb_00002blq	1	A	P00772	ELASTASE 1	Hydrolase	3.4.21.36	0.32
pdb_00002fi4	1	A	P00760	Cationic trypsin	Hydrolase	3.4.21.4	0.32
pdb_00002fi5	1	A	P00760	Cationic trypsin	Hydrolase	3.4.21.4	0.32
pdb_00002foa	1	A	P00772	elastase-1	Hydrolase	3.4.21.36	0.32
pdb_00002foc	1	A	P00772	elastase-1	Hydrolase	3.4.21.36	0.32
pdb_00002foh	1	A	P00772	elastase-1	Hydrolase	3.4.21.36	0.32
pdb_00002ftl	1	A	P00760	Cationic trypsin	Hydrolase	3.4.21.4	0.32
pdb_00002h1u	2	B	P00772	Elastase-1	Hydrolase	3.4.21.36	0.32
pdb_00003a86	1	A	P00760	Cationic trypsin	Hydrolase	3.4.21.4	0.32
pdb_00003d65	2	B	P00760	Cationic trypsin	Hydrolase	3.4.21.4	0.32
pdb_00003odf	1	A	P00772	Chymotrypsin-like	Hydrolase	3.4.21.36	0.32
pdb_00004iso	1	A	Q9Y5Y6	Suppressor of tur	Hydrolase	3.4.21.109	0.32
pdb_00004ktu	1	A	P00760	Cationic trypsin	Hydrolase	3.4.21.4	0.32
pdb_00004y0y	1	A	P00760	Cationic trypsin	Hydrolase	3.4.21.4	0.32
pdb_00005avd	1	A	P00772	Chymotrypsin-like	Hydrolase	3.4.21.36	0.32

PDB ID	Entity ID	Chain ID(s)	UniProt ID(s)	Protein(s)	EC Class	EC Number(s)	RMSD
pdb_00006avl	1	A	P00760	Cationic trypsin	Hydrolase	3.4.21.4	0.32
pdb_00006t0m	1	A	P00760	Cationic trypsin	Hydrolase	3.4.21.4	0.32
pdb_00006t5w	1	A	P00760	Cationic Trypsin	Hydrolase	3.4.21.4	0.32
pdb_00008g1v	1	A	Q9Y5Y6	Suppressor of tun	Hydrolase	3.4.21.109	0.32
pdb_00008g1w	1	A	Q9Y5Y6	Suppressor of tun	Hydrolase	3.4.21.109	0.32
pdb_00009pd9	1	A	P00761	Trypsin	Hydrolase	3.4.21.4	0.32
pdb_00001bra	1	A	P00763	TRYPSIN	Hydrolase	3.4.21.4	0.33
pdb_00001btu	1	A	P00772	ELASTASE	Hydrolase	3.4.21.36	0.33
pdb_00001f7z	1	A	P00763	TRYPSIN II, ANIOI	Hydrolase	3.4.21.4	0.33
pdb_00001fxy	1	A	P00742	COAGULATION F/	Hydrolase	3.4.21.6	0.33
pdb_00001h9l	2	B	P00772	ELASTASE	Hydrolase	3.4.21.36	0.33
pdb_00001nc6	1	A	P00760	Trypsinogen	Hydrolase	3.4.21.4	0.33
pdb_00001ox1	1	A	P00760	Trypsinogen, catio	Hydrolase	3.4.21.4	0.33
pdb_00001p2i	1	A	P00760	Trypsinogen, catio	Hydrolase	3.4.21.4	0.33
pdb_00001tpa	1	A	P00760	ANHYDRO-TRYPS	Hydrolase	3.4.21.4	0.33
pdb_00001tps	1	A	P00760	TRYPSIN	Hydrolase	3.4.21.4	0.33
pdb_00001uvp	1	A	P00772	ELASTASE 1	Hydrolase	3.4.21.36	0.33
pdb_00002a31	1	A	P00761	Trypsin	Hydrolase	3.4.21.4	0.33
pdb_00002bd9	1	A	P00772	Elastase-1	Hydrolase	3.4.21.36	0.33
pdb_00002fi3	1	A	P00760	Cationic trypsin	Hydrolase	3.4.21.4	0.33
pdb_00002fob	1	A	P00772	elastase-1	Hydrolase	3.4.21.36	0.33
pdb_00002fod	1	A	P00772	elastase-1	Hydrolase	3.4.21.36	0.33
pdb_00002fww	1	D	P20231	Tryptase beta-2	Hydrolase	3.4.21.59	0.33
pdb_00003e16	1	A	Q16651	Prostasin	Hydrolase	3.4.21	0.33
pdb_00003mnb	1	A	P00772	Chymotrypsin-like	Hydrolase	3.4.21.36	0.33
pdb_00003mo9	1	A	P00772	Chymotrypsin-like	Hydrolase	3.4.21.36	0.33
pdb_00003mty	1	A	P00772	Chymotrypsin-like	Hydrolase	3.4.21.36	0.33
pdb_00003mu0	1	A	P00772	Chymotrypsin-like	Hydrolase	3.4.21.36	0.33
pdb_00003mu4	1	A	P00772	Chymotrypsin-like	Hydrolase	3.4.21.36	0.33
pdb_00003odd	1	A	P00772	Chymotrypsin-like	Hydrolase	3.4.21.36	0.33
pdb_00003tgi	1	A	P00763	TRYPSIN	Hydrolase	3.4.21.4	0.33
pdb_00004abj	1	A	P00760	CATIONIC TRYPSI	Hydrolase	3.4.21.4	0.33
pdb_00005mnh	1	A	P00760	Cationic trypsin	Hydrolase	3.4.21.4	0.33
pdb_00005nx1	1	A	Q92876	Kallikrein-6	Hydrolase	3.4.21	0.33
pdb_00006dwf	1	G	P00760	Cationic trypsin	Hydrolase	3.4.21.4	0.33
pdb_00006e5m	1	A	P00760	Cationic trypsin	Hydrolase	3.4.21.4	0.33
pdb_00006n4t	1	A	Q9Y5Y6	Suppressor of tun	Hydrolase	3.4.21.109	0.33
pdb_00006ydy	1	A	P00760	Cationic Trypsin	Hydrolase	3.4.21.4	0.33

PDB ID	Entity ID	Chain ID(s)	UniProt ID(s)	Protein(s)	EC Class	EC Number(s)	RMSD
pdb_00006zq2	1	A	P00760	Cationic trypsin	Hydrolase	3.4.21.4	0.33
pdb_00007fbp	1	A	P00748	Coagulation facto	Hydrolase	3.4.21.38	0.33
pdb_00007ph1	1	A	P00760	Cationic trypsin	Hydrolase	3.4.21.4	0.33
pdb_00008b04	1	A	P00772	Chymotrypsin-like	Hydrolase	3.4.21.36	0.33
pdb_00008zvs	1	A		snFPITE-n1			0.33
pdb_00009e83	1	A	O15393	Transmembrane p	Hydrolase	3.4.21.122	0.33
pdb_00001c1m	1	A	P00772	PROTEIN (PORCI	Hydrolase	3.4.21.36	0.34
pdb_00001ela	1	A	P00772	ELASTASE	Hydrolase	3.4.21.36	0.34
pdb_00001elb	1	A	P00772	ELASTASE	Hydrolase	3.4.21.36	0.34
pdb_00001elf	1	A	P00772	PORCINE PANCRI	Hydrolase	3.4.21.36	0.34
pdb_00001elg	1	A	P00772	PORCINE PANCRI	Hydrolase	3.4.21.36	0.34
pdb_00001fy8	1	A	P00763	TRYPSIN II, ANIOI	Hydrolase	3.4.21.4	0.34
pdb_00001haz	2	B	P00772	ELASTASE 1	Hydrolase	3.4.21.36	0.34
pdb_00001tfx	1	B	P00761	TRYPSIN	Hydrolase	3.4.21.4	0.34
pdb_00001zr0	1	A	P00760	Cationic trypsin	Hydrolase	3.4.21.4	0.34
pdb_00002a7j	1	A	P00772	Elastase 1	Hydrolase	3.4.21.36	0.34
pdb_00002bb4	2	B	P00772	Chymotrypsin-like	Hydrolase	3.4.21.36	0.34
pdb_00002bd2	2	B	P00772	Chymotrypsin-like	Hydrolase	3.4.21.36	0.34
pdb_00002bya	1	A	P00760	CATIONIC TRYPSI	Hydrolase	3.4.21.4	0.34
pdb_00002foe	1	A	P00772	elastase-1	Hydrolase	3.4.21.36	0.34
pdb_00002g4t	1	A	P00772	Elastase-1	Hydrolase	3.4.21.36	0.34
pdb_00002g4u	1	A	P00772	elastase-1	Hydrolase	3.4.21.36	0.34
pdb_00002psx	1	A	Q9Y337	Kallikrein-5	Hydrolase	3.4.21	0.34
pdb_00002zdk	1	A	P00760	Cationic trypsin	Hydrolase	3.4.21.4	0.34
pdb_00003btk	1	A	P00760	PROTEIN (TRYPSI	Hydrolase	3.4.21.4	0.34
pdb_00003mnc	1	A	P00772	Chymotrypsin-like	Hydrolase	3.4.21.36	0.34
pdb_00003mu1	1	A	P00772	Chymotrypsin-like	Hydrolase	3.4.21.36	0.34
pdb_00003mu5	1	A	P00772	Chymotrypsin-like	Hydrolase	3.4.21.36	0.34
pdb_00004aoq	1	C	P00760	CATIONIC TRYPSI	Hydrolase	3.4.21.4	0.34
pdb_00004est	1	A	P00772	ELASTASE	Hydrolase	3.4.21.36	0.34
pdb_00004y10	1	A	P00760	Cationic trypsin	Hydrolase	3.4.21.4	0.34
pdb_00004y11	1	A	P00760	Cationic trypsin	Hydrolase	3.4.21.4	0.34
pdb_00006b6r	1	A	P00760	Cationic trypsin	Hydrolase	3.4.21.4	0.34
pdb_00006skc	1	B	Q92876	Kallikrein-6	Hydrolase	3.4.21	0.34
pdb_00009awr	1	A	P00760	Cationic trypsin	Hydrolase	3.4.21.4	0.34
pdb_00001eja	1	A	P00761	TRYPSIN	Hydrolase	3.4.21.4	0.35
pdb_00001gvk	2	B	P00772	ELASTASE 1	Hydrolase	3.4.21.36	0.35
pdb_00001hax	2	B	P00772	ELASTASE 1	Hydrolase	3.4.21.36	0.35

PDB ID	Entity ID	Chain ID(s)	UniProt ID(s)	Protein(s)	EC Class	EC Number(s)	RMSD
pdb_00001lvy	1	A	P00772	ELASTASE	Hydrolase	3.4.21.36	0.35
pdb_00001yc0	1	A	Q04756	Hepatocyte growt	Hydrolase	3.4.21	0.35
pdb_00002bd4	2	B	P00772	Chymotrypsin-like	Hydrolase	3.4.21.36	0.35
pdb_00002bd7	2	B	P00772	Chymotrypsin-like	Hydrolase	3.4.21.36	0.35
pdb_00002bd8	2	B	P00772	Chymotrypsin-like	Hydrolase	3.4.21.36	0.35
pdb_00002by6	1	A	P00760	CATIONIC TRYPSI	Hydrolase	3.4.21.4	0.35
pdb_00002by7	1	A	P00760	CATIONIC TRYPSI	Hydrolase	3.4.21.4	0.35
pdb_00002by9	1	A	P00760	CATIONIC TRYPSI	Hydrolase	3.4.21.4	0.35
pdb_00002zfs	1	A	P00760	Cationic trypsin	Hydrolase	3.4.21.4	0.35
pdb_00003a84	1	A	P00760	Cationic trypsin	Hydrolase	3.4.21.4	0.35
pdb_00003a8a	1	A	P00760	Cationic trypsin	Hydrolase	3.4.21.4	0.35
pdb_00003a8d	1	A	P00760	Cationic trypsin	Hydrolase	3.4.21.4	0.35
pdb_00003mo3	1	A	P00772	Chymotrypsin-like	Hydrolase	3.4.21.36	0.35
pdb_00003otj	1	A	P00760	Cationic trypsin	Hydrolase	3.4.21.4	0.35
pdb_00004aor	1	B	P00760	CATIONIC TRYPSI	Hydrolase	3.4.21.4	0.35
pdb_00004y0z	1	A	P00760	Cationic trypsin	Hydrolase	3.4.21.4	0.35
pdb_00005f03	1	A	Q15661	Tryptase beta-2	Hydrolase	3.4.21.59	0.35
pdb_00005mn1	1	A	P00760	Cationic trypsin	Hydrolase	3.4.21.4	0.35
pdb_00006b6n	1	A	P00760	Cationic trypsin	Hydrolase	3.4.21.4	0.35
pdb_00006d3y	1	A	P00747	Plasminogen	Hydrolase	3.4.21.7	0.35
pdb_00006d40	1	A	P00747	Plasminogen	Hydrolase	3.4.21.7	0.35
pdb_00006dwh	1	G	P00760	Cationic trypsin	Hydrolase	3.4.21.4	0.35
pdb_00006vxy	1	A	P00760	Cationic trypsin	Hydrolase	3.4.21.4	0.35
pdb_00007pzo	1	A	P39675	mite allergen Der	Hydrolase	3.4.21	0.35
pdb_00008izk	1	A	P00760	Cationic trypsin	Hydrolase	3.4.21.4	0.35
pdb_00001an1	1	A	P00761	TRYPSIN	Hydrolase	3.4.21.4	0.36
pdb_00001eat	1	A	P00772	PORCINE PANCRI	Hydrolase	3.4.21.36	0.36
pdb_00001f5r	1	A	P00763	TRYPSIN II, ANIOI	Hydrolase	3.4.21.4	0.36
pdb_00001g3c	1	A	P00760	BETA-TRYPSIN	Hydrolase	3.4.21.4	0.36
pdb_00001p2j	1	A	P00760	Trypsinogen, cati	Hydrolase	3.4.21.4	0.36
pdb_00002by5	1	A	P00760	CATIONIC TRYPSI	Hydrolase	3.4.21.4	0.36
pdb_00002by8	1	A	P00760	CATIONIC TRYPSI	Hydrolase	3.4.21.4	0.36
pdb_00002iot	1	A	P00772	Elastase-1	Hydrolase	3.4.21.36	0.36
pdb_00002v0b	1	A	P00772	ELASTASE-1	Hydrolase	3.4.21.36	0.36
pdb_00002xtt	2	B	P00760	CATIONIC TRYPSI	Hydrolase	3.4.21.4	0.36
pdb_00003a82	1	A	P00760	Cationic trypsin	Hydrolase	3.4.21.4	0.36
pdb_00003btg	1	A	P00760	PROTEIN (TRYPSI	Hydrolase	3.4.21.4	0.36
pdb_00003bth	1	A	P00760	PROTEIN (TRYPSI	Hydrolase	3.4.21.4	0.36

PDB ID	Entity ID	Chain ID(s)	UniProt ID(s)	Protein(s)	EC Class	EC Number(s)	RMSD
pdb_00003gy7	1	A	P00760	Cationic trypsin	Hydrolase	3.4.21.4	0.36
pdb_00003mns	1	A	P00772	Chymotrypsin-like	Hydrolase	3.4.21.36	0.36
pdb_00003mnx	1	A	P00772	Chymotrypsin-like	Hydrolase	3.4.21.36	0.36
pdb_00003mo6	1	A	P00772	Chymotrypsin-like	Hydrolase	3.4.21.36	0.36
pdb_00003p8f	1	A	Q9Y5Y6	ST14 protein	Hydrolase	3.4.21.109	0.36
pdb_00004bnr	1	A	Q52V24	HEPATOPANCREA	Hydrolase	3.4.21.4	0.36
pdb_00004mtb	1	A	P00760	Cationic trypsin	Hydrolase	3.4.21.4	0.36
pdb_00005mnq	1	A	P00760	Cationic trypsin	Hydrolase	3.4.21.4	0.36
pdb_00006u22	1	A	P00760	Cationic trypsin	Hydrolase	3.4.21.4	0.36
pdb_00008b49	1	A	P00772	Chymotrypsin-like	Hydrolase	3.4.21.36	0.36
pdb_00009pdc	1	A	P00761	Trypsin	Hydrolase	3.4.21.4	0.36
pdb_00009qfv	1	C	P20231	Tryptase beta-2	Hydrolase	3.4.21.59	0.36
pdb_00001btw	1	A	P00760	BETA-TRYPSIN	Hydrolase	3.4.21.4	0.37
pdb_00001eaw	1	A	Q9Y5Y6	SUPPRESSOR OF	Hydrolase	3.4.21.109	0.37
pdb_00001fzz	1	A	P00772	ELASTASE 1	Hydrolase	3.4.21.36	0.37
pdb_00001s5s	1	A	P00761	Trypsin	Hydrolase	3.4.21.4	0.37
pdb_00002bd5	2	B	P00772	Chymotrypsin-like	Hydrolase	3.4.21.36	0.37
pdb_00002zdn	1	A	P00760	Cationic trypsin	Hydrolase	3.4.21.4	0.37
pdb_00003a7t	1	A	P00760	Cationic trypsin	Hydrolase	3.4.21.4	0.37
pdb_00003a7w	1	A	P00760	Cationic trypsin	Hydrolase	3.4.21.4	0.37
pdb_00003btq	1	A	P00760	PROTEIN (TRYPSI	Hydrolase	3.4.21.4	0.37
pdb_00003mu8	1	A	P00772	Chymotrypsin-like	Hydrolase	3.4.21.36	0.37
pdb_00006t9u	1	A	P00760	Cationic Trypsin	Hydrolase	3.4.21.4	0.37
pdb_00006yiu	1	A	P00760	Cationic trypsin	Hydrolase	3.4.21.4	0.37
pdb_00006yix	1	A	P00760	Cationic trypsin	Hydrolase	3.4.21.4	0.37
pdb_00008b53	1	A	P00772	Chymotrypsin-like	Hydrolase	3.4.21.36	0.37
pdb_00008hd8	2	D	O15393	Transmembrane p	Hydrolase	3.4.21.122	0.37
pdb_00008xni	1	A	P00760	Cationic trypsin	Hydrolase	3.4.21.4	0.37
pdb_00001mcv	1	A	P00772	Elastase 1	Hydrolase	3.4.21.36	0.38
pdb_00001nes	1	A	P00772	ELASTASE	Hydrolase	3.4.21.36	0.38
pdb_00001taw	1	A	P00760	TRYPSIN	Hydrolase	3.4.21.4	0.38
pdb_00002ptc	1	A	P00760	BETA-TRYPSIN	Hydrolase	3.4.21.4	0.38
pdb_00003btt	1	A	P00760	PROTEIN (TRYPSI	Hydrolase	3.4.21.4	0.38
pdb_00003tpi	1	A	P00760	TRYPSINOGEN	Hydrolase	3.4.21.4	0.38
pdb_00005xwl	1	A	P00761	Trypsin	Hydrolase	3.4.21.4	0.38
pdb_00007bs7	1	A	P00760	Cationic trypsin	Hydrolase	3.4.21.4	0.38
pdb_00001a5i	1	A	P98119	PLASMINOGEN A	Hydrolase	3.4.21.68	0.39
pdb_00001eas	1	A	P00772	PORCINE PANCRI	Hydrolase	3.4.21.36	0.39

PDB ID	Entity ID	Chain ID(s)	UniProt ID(s)	Protein(s)	EC Class	EC Number(s)	RMSD
pdb_00001p2k	1	A	P00760	Trypsinogen, catio	Hydrolase	3.4.21.4	0.39
pdb_00002zhd	1	A	P00760	Cationic trypsin	Hydrolase	3.4.21.4	0.39
pdb_00003btd	1	A	P00760	PROTEIN (TRYPSI	Hydrolase	3.4.21.4	0.39
pdb_00003nkk	1	A	P00760	Cationic trypsin	Hydrolase	3.4.21.4	0.39
pdb_00004tpi	1	A	P00760	TRYPSINOGEN	Hydrolase	3.4.21.4	0.39
pdb_00005hgg	1	B	P00749	Urokinase-type pla	Hydrolase	3.4.21.73	0.39
pdb_00006kd5	2	B	Q9BYE2	Transmembrane p	Hydrolase	3.4.21	0.39
pdb_00007y0f	2	D	O15393	Transmembrane p	Hydrolase	3.4.21.122	0.39
pdb_00001anc	1	A	P00763	ANIONIC TRYPSIN	Hydrolase	3.4.21.4	0.4
pdb_00001brb	1	A	P00763	TRYPSIN	Hydrolase	3.4.21.4	0.4
pdb_00002zpq	1	A	Q8AV11	Anionic trypsin	Hydrolase	3.4.21.4	0.4
pdb_00003a7y	1	A	P00760	Cationic trypsin	Hydrolase	3.4.21.4	0.4
pdb_00003btf	1	A	P00760	PROTEIN (TRYPSI	Hydrolase	3.4.21.4	0.4
pdb_00003gy3	1	A	P00760	Cationic trypsin	Hydrolase	3.4.21.4	0.4
pdb_00005est	1	A	P00772	ELASTASE	Hydrolase	3.4.21.36	0.4
pdb_00005nx3	1	A	Q92876	Kallikrein-6	Hydrolase	3.4.21	0.4
pdb_00006yis	1	A	P00760	Cationic Trypsin	Hydrolase	3.4.21.4	0.4
pdb_00007jr2	1	C	P00760	Cationic trypsin	Hydrolase	3.4.21.4	0.4
pdb_00001ane	1	A	P00763	ANIONIC TRYPSIN	Hydrolase	3.4.21.4	0.41
pdb_00001c9p	1	A	P00761	TRYPSIN	Hydrolase	3.4.21.4	0.41
pdb_00001qix	2	B	P00772	ELASTASE	Hydrolase	3.4.21.36	0.41
pdb_00002tgp	1	A	P00760	TRYPSINOGEN	Hydrolase	3.4.21.4	0.41
pdb_00005mnm	1	A	P00760	Cationic trypsin	Hydrolase	3.4.21.4	0.41
pdb_00006gt6	1	A	P00748	Coagulation facto	Hydrolase	3.4.21.38	0.41
pdb_00007jr1	1	C	P00760	Cationic trypsin	Hydrolase	3.4.21.4	0.41
pdb_00001eau	1	A	P00772	PORCINE PANCRE	Hydrolase	3.4.21.36	0.42
pdb_00002f91	1	A	Q52V24	hepatopancreas t	Hydrolase	3.4.21.4	0.42
pdb_00003myw	1	A	P00761	Trypsin	Hydrolase	3.4.21.4	0.42
pdb_00001fle	1	A	P00772	ELASTASE	Hydrolase	3.4.21.36	0.43
pdb_00003t26	1	A	P00760	Cationic trypsin	Hydrolase	3.4.21.4	0.43
pdb_00004isn	2	B	Q9Y5Y6	Suppressor of tun	Hydrolase	3.4.21.109	0.43
pdb_00005mnl	1	A	P00760	Cationic trypsin	Hydrolase	3.4.21.4	0.43
pdb_00006dwu	1	UB	P00760	Cationic trypsin	Hydrolase	3.4.21.4	0.43
pdb_00007wa0	1	A	P00760	Cationic trypsin	Hydrolase	3.4.21.4	0.43
pdb_00004bxw	1	A	Q56VR3	FACTOR XA	Hydrolase	3.4.21.6	0.44
pdb_00005ms4	1	B	O60259	Kallikrein-8	Hydrolase	3.4.21.118	0.44
pdb_00006mv4	1	A	P00740	Coagulation facto	Hydrolase	3.4.21.22	0.44
pdb_00001aq7	1	A	P00760	TRYPSIN	Hydrolase	3.4.21.4	0.45

PDB ID	Entity ID	Chain ID(s)	UniProt ID(s)	Protein(s)	EC Class	EC Number(s)	RMSD
pdb_00001bui	1	A	P00747	Plasminogen	Hydrolase	3.4.21.7	0.45
pdb_00006qbu	1	A	P00772	Chymotrypsin-like	Hydrolase	3.4.21.36	0.45
pdb_00006qen	1	A	P00772	Chymotrypsin-like	Hydrolase	3.4.21.36	0.45
pdb_00006qeo	1	A	P00772	Chymotrypsin-like	Hydrolase	3.4.21.36	0.45
pdb_00006xyg	1	A	P00760	Cationic trypsin	Hydrolase	3.4.21.4	0.45
pdb_00001xug	1	A	P00760	TRYPSIN	Hydrolase	3.4.21.4	0.46
pdb_00007al8	1	K	P00760	Cationic trypsin	Hydrolase	3.4.21.4	0.46
pdb_00001fiz	1	A	P08001	BETA-ACROSIN H	Hydrolase	3.4.21.10	0.47
pdb_00002tpi	1	A	P00760	TRYPSINOGEN	Hydrolase	3.4.21.4	0.47
pdb_00002wph	3	C	P00740	COAGULATION FA	Hydrolase	3.4.21.22	0.47
pdb_00003a8b	1	A	P00760	Cationic trypsin	Hydrolase	3.4.21.4	0.47
pdb_00003moc	1	A	P00772	Chymotrypsin-like	Hydrolase	3.4.21.36	0.47
pdb_00001b0e	1	A	P00772	PROTEIN (ELASTA	Hydrolase	3.4.21.36	0.48
pdb_00001qr3	1	A	P00772	Chymotrypsin-like	Hydrolase	3.4.21.36	0.48
pdb_00002d8w	1	A	P00760	Cationic trypsin	Hydrolase	3.4.21.4	0.48
pdb_00003a87	1	A	P00760	Cationic trypsin	Hydrolase	3.4.21.4	0.48
pdb_00003hgn	1	A	P00772	Elastase-1	Hydrolase	3.4.21.36	0.48
pdb_00006th7	1	A	P00772	Chymotrypsin-like	Hydrolase	3.4.21.36	0.48
pdb_00001bda	1	B	P00750	SINGLE CHAIN TI	Hydrolase	3.4.21.68	0.49
pdb_00001lmw	2	D	P00749	UROKINASE-TYPE	Hydrolase	3.4.21.73	0.49
pdb_00001okx	1	A	P00772	ELASTASE 1	Hydrolase	3.4.21.36	0.49
pdb_00003a8c	1	A	P00760	Cationic trypsin	Hydrolase	3.4.21.4	0.49
pdb_00003hgp	1	A	P00772	Elastase-1	Hydrolase	3.4.21.36	0.49
pdb_00003t28	1	A	P00760	Cationic trypsin	Hydrolase	3.4.21.4	0.49
pdb_00005jbc	2	B	P00740	Coagulation facto	Hydrolase	3.4.21.22	0.49
pdb_00006l63	1	A	P00748	Coagulation facto	Hydrolase	3.4.21.38	0.49
pdb_00007ays	1	A	P00760	Cationic trypsin	Hydrolase	3.4.21.4	0.49
pdb_00009est	1	A	P00772	PORCINE PANCRI	Hydrolase	3.4.21.36	0.49
pdb_00002oq5	1	A	Q9UL52	Transmembrane p	Hydrolase	3.4.21	0.5
pdb_00002r0k	1	A	Q04756	Hepatocyte growt	Hydrolase	3.4.21	0.5
pdb_00004gvu	1	A	P00772	Chymotrypsin-like	Hydrolase	3.4.21.36	0.5
pdb_00009cm2	2	D	P00742	Coagulation facto	Hydrolase	3.4.21.6	0.5
pdb_00003t25	1	A	P00760	Cationic trypsin	Hydrolase	3.4.21.4	0.51
pdb_00009aws	1	A	P00760	Cationic trypsin	Hydrolase	3.4.21.4	0.51
pdb_00001uhb	2	A,B	P00761	Trypsin	Hydrolase	3.4.21.4	0.52
pdb_00002cv3	1	A	P00772	Elastase 1	Hydrolase	3.4.21.36	0.52
pdb_00002p3f	1	A	P00742	Coagulation facto	Hydrolase	3.4.21.6	0.52
pdb_00002wpk	3	C	P00740	COAGULATION FA	Hydrolase	3.4.21.22	0.52

PDB ID	Entity ID	Chain ID(s)	UniProt ID(s)	Protein(s)	EC Class	EC Number(s)	RMSD
pdb_00007jwx	1	A	P00760	Cationic trypsin	Hydrolase	3.4.21.4	0.52
pdb_00009cli	2	D	P00742	Coagulation facto	Hydrolase	3.4.21.6	0.52
pdb_00001anb	1	A	P00763	ANIONIC TRYPSIN	Hydrolase	3.4.21.4	0.53
pdb_00001yf4	1	A	P00761	Trypsin	Hydrolase	3.4.21.4	0.53
pdb_00002r2w	1	A	P00749	Plasminogen acti	Hydrolase	3.4.21.73	0.53
pdb_00003btm	1	A	P00760	PROTEIN (TRYPSI	Hydrolase	3.4.21.4	0.53
pdb_00005gib	1	A	P00760	Cationic trypsin	Hydrolase	3.4.21.4	0.53
pdb_00006eav	1	A	P00760	Cationic trypsin	Hydrolase	3.4.21.4	0.53
pdb_00006p0p	1	A	Q15661	Tryptase alpha/be	Hydrolase	3.4.21.59	0.54
pdb_00009p77	1	A	P00760	Pretrypsinogen I	Hydrolase	3.4.21.4	0.54
pdb_00001ept	3	A,B,C	P00761	PORCINE E-TRYP:	Hydrolase	3.4.21.4	0.55
pdb_00001fn6	1	A	P00761	TRYPSIN	Hydrolase	3.4.21.4	0.55
pdb_00001utn	1	A	P00760	TRYPSINOGEN	Hydrolase	3.4.21.4	0.55
pdb_00002blv	1	A	P00760	TRYPSIN	Hydrolase	3.4.21.4	0.55
pdb_00002blw	1	A	P00760	TRYPSIN	Hydrolase	3.4.21.4	0.55
pdb_00002eek	1	A	P16049	Trypsin-1	Hydrolase	3.4.21.4	0.55
pdb_00003ru4	1	A	P00760	Cationic trypsin	Hydrolase	3.4.21.4	0.55
pdb_00004an7	1	A	P00761	TRYPSIN	Hydrolase	3.4.21.4	0.55
pdb_00004mpw	1	A	Q15661	Tryptase alpha/be	Hydrolase	3.4.21.59	0.55
pdb_00009avz	1	A	P00760	Cationic trypsin	Hydrolase	3.4.21.4	0.55
pdb_00009awg	1	A	P00760	Cationic trypsin	Hydrolase	3.4.21.4	0.55
pdb_00009awm	1	A	P00760	Cationic trypsin	Hydrolase	3.4.21.4	0.55
pdb_00009awp	1	A	P00760	Cationic trypsin	Hydrolase	3.4.21.4	0.55
pdb_00001aks	2	A,B	P00761	ALPHA TRYPSIN	Hydrolase	3.4.21.4	0.56
pdb_00001bjv	1	A	P00760	BETA-TRYPSIN	Hydrolase	3.4.21.4	0.56
pdb_00001ce5	1	A	P00760	PROTEIN (TRYPSI	Hydrolase	3.4.21.4	0.56
pdb_00001fmg	1	A	P00761	TRYPSIN	Hydrolase	3.4.21.4	0.56
pdb_00001hj8	1	A	P35031	TRYPSIN I	Hydrolase	3.4.21.4	0.56
pdb_00001qqu	1	A	P00761	BETA TRYPSIN	Hydrolase	3.4.21.4	0.56
pdb_00002de9	1	A	P00772	Elastase-1	Hydrolase	3.4.21.36	0.56
pdb_00002o9q	1	A	P00760	Cationic trypsin	Hydrolase	3.4.21.4	0.56
pdb_00002wpj	3	C	P00740	COAGULATION F/	Hydrolase	3.4.21.22	0.56
pdb_00009awd	1	A	P00760	Cationic trypsin	Hydrolase	3.4.21.4	0.56
pdb_00001bzx	1	A	P35031	PROTEIN (TRYPSI	Hydrolase	3.4.21.4	0.57
pdb_00001v6d	1	A	P00761	Trypsin	Hydrolase	3.4.21.4	0.57
pdb_00002btc	1	A	P00760	PROTEIN (TRYPSI	Hydrolase	3.4.21.4	0.57
pdb_00002de8	1	A	P00772	Elastase-1	Hydrolase	3.4.21.36	0.57
pdb_00002g55	1	A	P00760	Cationic trypsin	Hydrolase	3.4.21.4	0.57

PDB ID	Entity ID	Chain ID(s)	UniProt ID(s)	Protein(s)	EC Class	EC Number(s)	RMSD
pdb_00002wpl	3	C	P00740	COAGULATION FA	Hydrolase	3.4.21.22	0.57
pdb_00003bte	1	A	P00760	TRYPSIN	Hydrolase	3.4.21.4	0.57
pdb_00007wr7	2	B	P98073	Enteropeptidase c	Hydrolase	3.4.21.9	0.57
pdb_00009awb	1	A	P00760	Cationic trypsin	Hydrolase	3.4.21.4	0.57
pdb_00009i2h	1	A	P00742	Activated factor X	Hydrolase	3.4.21.6	0.57
pdb_00001c9t	1	C	P00760	TRYPSIN	Hydrolase	3.4.21.4	0.58
pdb_00001elc	1	A	P00772	ELASTASE	Hydrolase	3.4.21.36	0.58
pdb_00001eld	1	A	P00772	ELASTASE	Hydrolase	3.4.21.36	0.58
pdb_00001ele	1	A	P00772	ELASTASE	Hydrolase	3.4.21.36	0.58
pdb_00001fni	1	A	P00761	TRYPSIN	Hydrolase	3.4.21.4	0.58
pdb_00001mct	1	A	P00761	BETA-TRYPSIN	Hydrolase	3.4.21.4	0.58
pdb_00001tgs	1	A	P00760	TRYPSINOGEN	Hydrolase	3.4.21.4	0.58
pdb_00002fo9	1	A	P00772	elastase-1	Hydrolase	3.4.21.36	0.58
pdb_00002g81	1	A	P00760	Cationic trypsin	Hydrolase	3.4.21.4	0.58
pdb_00003m7q	1	A	P00760	Cationic trypsin	Hydrolase	3.4.21.4	0.58
pdb_00004ym9	1	A	P00772	Chymotrypsin-like	Hydrolase	3.4.21.36	0.58
pdb_00007ahu	3	G	P00742	Coagulation facto	Hydrolase	3.4.21.6	0.58
pdb_00001auj	1	A	P00760	TRYPSIN	Hydrolase	3.4.21.4	0.59
pdb_00001avw	1	A	P00761	TRYPSIN	Hydrolase	3.4.21.4	0.59
pdb_00001bju	1	A	P00760	BETA-TRYPSIN	Hydrolase	3.4.21.4	0.59
pdb_00001tx6	1	A	P00761	Trypsin	Hydrolase	3.4.21.4	0.59
pdb_00002iln	1	B	P00760	Cationic trypsin	Hydrolase	3.4.21.4	0.59
pdb_00002oxs	1	A	P00760	Cationic trypsin	Hydrolase	3.4.21.4	0.59
pdb_00002wpm	3	C	P00740	COAGULATION FA	Hydrolase	3.4.21.22	0.59
pdb_00006vvu	1	D	Q15661	Tryptase alpha/be	Hydrolase	3.4.21.59	0.59
pdb_00001d6r	1	A	P00760	TRYPSINOGEN	Hydrolase	3.4.21.4	0.6
pdb_00001s81	1	A	P00761	TRYPSIN	Hydrolase	3.4.21.4	0.6
pdb_00001s83	1	A	P00761	TRYPSIN	Hydrolase	3.4.21.4	0.6
pdb_00001v2m	1	A	P00760	Trypsin	Hydrolase	3.4.21.4	0.6
pdb_00002wuc	1	A	Q04756	HEPATOCYTE GR	Hydrolase	3.4.21	0.6
pdb_00002zft	1	A	P00760	Cationic trypsin	Hydrolase	3.4.21.4	0.6
pdb_00001eai	1	B	P00772	PROTEIN (ELASTA	Hydrolase	3.4.21.36	0.61
pdb_00001eb2	1	A	P00760	TRYPSIN	Hydrolase	3.4.21.4	0.61
pdb_00001z7k	1	A	P00761	Trypsin	Hydrolase	3.4.21.4	0.61
pdb_00002ah4	1	A	P00760	beta-trypsin	Hydrolase	3.4.21.4	0.61
pdb_00002psy	1	A	Q9Y337	Kallikrein-5	Hydrolase	3.4.21	0.61
pdb_00003iti	1	A	P00760	Cationic trypsin	Hydrolase	3.4.21.4	0.61
pdb_00003ptn	1	A	P00760	TRYPSIN	Hydrolase	3.4.21.4	0.61

PDB ID	Entity ID	Chain ID(s)	UniProt ID(s)	Protein(s)	EC Class	EC Number(s)	RMSD
pdb_00004tpy	1	A	P00760	Cationic trypsin	Hydrolase	3.4.21.4	0.61
pdb_00007qfv	1	B	Q92876	Kallikrein-6	Hydrolase	3.4.21	0.61
pdb_00001bma	1	A	P00772	Chymotrypsin-like	Hydrolase	3.4.21.36	0.62
pdb_00001c1q	1	A	P00760	TRYPSIN	Hydrolase	3.4.21.4	0.62
pdb_00001c1t	1	A	P00760	TRYPSIN	Hydrolase	3.4.21.4	0.62
pdb_00001gbt	1	A	P00760	BETA-TRYPSIN	Hydrolase	3.4.21.4	0.62
pdb_00001mmj	1	A	P00772	elastase 1	Hydrolase	3.4.21.36	0.62
pdb_00002fmj	1	A	P00775	Trypsin	Hydrolase	3.4.21.4	0.62
pdb_00003uou	1	A	P00772	Chymotrypsin-like	Hydrolase	3.4.21.36	0.62
pdb_00001k1o	1	A	P00760	TRYPSIN	Hydrolase	3.4.21.4	0.63
pdb_00002g5v	1	A	P00760	Cationic trypsin	Hydrolase	3.4.21.4	0.63
pdb_00002j9n	1	A	P00760	CATIONIC TRYPSI	Hydrolase	3.4.21.4	0.63
pdb_00002zdm	1	A	P00760	Cationic trypsin	Hydrolase	3.4.21.4	0.63
pdb_00004mpu	1	A	Q15661	Tryptase alpha/be	Hydrolase	3.4.21.59	0.63
pdb_00004mpv	1	A	Q15661	Tryptase alpha/be	Hydrolase	3.4.21.59	0.63
pdb_00002qyi	1	A	P00760	Cationic trypsin	Hydrolase	3.4.21.4	0.64
pdb_00002vu8	1	A	P35049	TRYPSIN	Hydrolase	3.4.21.4	0.64
pdb_00002zdl	1	A	P00760	Cationic trypsin	Hydrolase	3.4.21.4	0.64
pdb_00002zpr	1	A	B3Y8K5	Anionic trypsin	Hydrolase	3.4.21.4	0.64
pdb_00003t29	1	A	P00760	Cationic trypsin	Hydrolase	3.4.21.4	0.64
pdb_00004ab9	1	A	P00760	CATIONIC TRYPSI	Hydrolase	3.4.21.4	0.64
pdb_00001hj9	1	A	P00760	BETA-TRYPSIN	Hydrolase	3.4.21.4	0.65
pdb_00001qa0	1	A	P00760	TRYPSIN	Hydrolase	3.4.21.4	0.65
pdb_00001utk	1	A	P35031	TRYPSIN I	Hydrolase	3.4.21.4	0.65
pdb_00002a7h	1	A	P00760	Cationic trypsin	Hydrolase	3.4.21.4	0.65
pdb_00002fx6	1	A	P00760	Trypsin	Hydrolase	3.4.21.4	0.65
pdb_00008wk1	1	C	P00760	Cationic trypsin	Hydrolase	3.4.21.4	0.65
pdb_00009pda	1	A	P00761	Trypsin	Hydrolase	3.4.21.4	0.66
pdb_00003btw	1	A	P00760	PROTEIN (TRYPSI	Hydrolase	3.4.21.4	0.67
pdb_00003gym	1	C	Q16651	Prostasin	Hydrolase	3.4.21	0.67
pdb_00003rxg	1	A	P00760	Cationic trypsin	Hydrolase	3.4.21.4	0.67
pdb_00003tk5	1	A	P00742	Factor X heavy ch	Hydrolase	3.4.21.6	0.67
pdb_00003veq	1	A	P00760	Cationic trypsin	Hydrolase	3.4.21.4	0.67
pdb_00004mqa	1	B	Q15661	Tryptase alpha/be	Hydrolase	3.4.21.59	0.67
pdb_00001rfn	1	A	P00740	PROTEIN (COAGU	Hydrolase	3.4.21.22	0.68
pdb_00001xka	2	B	P00742	BLOOD COAGULA	Hydrolase	3.4.21.6	0.68
pdb_00003a7z	1	A	P00760	Cationic trypsin	Hydrolase	3.4.21.4	0.68
pdb_00003ptb	1	A	P00760	BETA-TRYPSIN	Hydrolase	3.4.21.4	0.68

PDB ID	Entity ID	Chain ID(s)	UniProt ID(s)	Protein(s)	EC Class	EC Number(s)	RMSD
pdb_00004a6l	1	A	P20231	TRYPTASE ALPH/	Hydrolase	3.4.21.59	0.68
pdb_00004mpx	1	B	Q15661	Tryptase alpha/be	Hydrolase	3.4.21.59	0.68
pdb_00001fiw	1	A	Q9GL10	BETA-ACROSIN H	Hydrolase	3.4.21.10	0.69
pdb_00001slw	2	B	P00763	ANIONIC TRYPSIN	Hydrolase	3.4.21.4	0.69
pdb_00001xuf	1	A	P00760	TRYPSIN	Hydrolase	3.4.21.4	0.7
pdb_00002ayw	1	A	P00760	Cationic trypsin	Hydrolase	3.4.21.4	0.7
pdb_00005mno	1	A	P00760	Cationic trypsin	Hydrolase	3.4.21.4	0.7
pdb_00001brc	1	A	P00763	TRYPSIN	Hydrolase	3.4.21.4	0.71
pdb_00001k1p	1	A	P00760	TRYPSIN	Hydrolase	3.4.21.4	0.71
pdb_00001may	1	A	P00760	BETA-TRYPSIN	Hydrolase	3.4.21.4	0.71
pdb_00001xuk	1	A	P00760	TRYPSIN	Hydrolase	3.4.21.4	0.71
pdb_00003e8l	2	C	P00760	Cationic trypsin	Hydrolase	3.4.21.4	0.71
pdb_00005mos	1	A	P00760	Cationic trypsin	Hydrolase	3.4.21.4	0.71
pdb_00005ugd	1	A	P00747	Plasminogen	Hydrolase	3.4.21.7	0.71
pdb_00007qi0	1	B	Q92876	Kallikrein-6	Hydrolase	3.4.21	0.71
pdb_00001c1n	1	A	P00760	TRYPSIN	Hydrolase	3.4.21.4	0.72
pdb_00001slv	2	B	P00763	ANIONIC TRYPSIN	Hydrolase	3.4.21.4	0.72
pdb_00001v2u	1	A	P00760	Trypsin	Hydrolase	3.4.21.4	0.72
pdb_00001xui	1	A	P00760	TRYPSIN	Hydrolase	3.4.21.4	0.72
pdb_00003i29	1	A	P00760	Cationic trypsin	Hydrolase	3.4.21.4	0.73
pdb_00005ugg	1	A	P00747	Plasminogen	Hydrolase	3.4.21.7	0.73
pdb_00007est	1	A	P00772	ELASTASE	Hydrolase	3.4.21.36	0.73
pdb_00001a0j	1	A	P35033	TRYPSIN	Hydrolase	3.4.21.4	0.74
pdb_00001c2k	1	A	P00760	TRYPSIN	Hydrolase	3.4.21.4	0.74
pdb_00001c2l	1	A	P00760	TRYPSIN	Hydrolase	3.4.21.4	0.74
pdb_00001c2m	1	A	P00760	TRYPSIN	Hydrolase	3.4.21.4	0.74
pdb_00001slx	2	B	P00763	ANIONIC TRYPSIN	Hydrolase	3.4.21.4	0.74
pdb_00001try	1	A	P35049	TRYPSIN	Hydrolase	3.4.21.4	0.74
pdb_00001xuj	1	A	P00760	TRYPSIN	Hydrolase	3.4.21.4	0.74
pdb_00003ljo	1	A	P00760	Cationic trypsin	Hydrolase	3.4.21.4	0.74
pdb_00005mnp	1	A	P00760	Cationic trypsin	Hydrolase	3.4.21.4	0.74
pdb_00001c2i	1	A	P00760	TRYPSIN	Hydrolase	3.4.21.4	0.75
pdb_00001g2m	1	A	P00742	COAGULATION F/	Hydrolase	3.4.21.6	0.75
pdb_00001v3x	1	A	P00742	Coagulation facto	Hydrolase	3.4.21.6	0.75
pdb_00001xuh	1	A	P00760	TRYPSIN	Hydrolase	3.4.21.4	0.75
pdb_00002bza	1	A	P00760	PROTEIN (TRYPSI	Hydrolase	3.4.21.4	0.75
pdb_00004j2y	2	B	P00760	Cationic trypsin	Hydrolase	3.4.21.4	0.75
pdb_00001c1o	1	A	P00760	TRYPSIN	Hydrolase	3.4.21.4	0.76

PDB ID	Entity ID	Chain ID(s)	UniProt ID(s)	Protein(s)	EC Class	EC Number(s)	RMSD
pdb_00001c5p	1	A	P00760	PROTEIN (TRYPSI	Hydrolase	3.4.21.4	0.76
pdb_00001mts	1	A	P00760	TRYPSIN	Hydrolase	3.4.21.4	0.76
pdb_00003plp	1	A	P00760	Cationic trypsin	Hydrolase	3.4.21.4	0.76
pdb_00005lh8	1	A	P00760	Cationic trypsin	Hydrolase	3.4.21.4	0.76
pdb_00007qhz	1	A	Q92876	Kallikrein-6	Hydrolase	3.4.21	0.76
pdb_00001c5v	1	A	P00760	PROTEIN (TRYPSI	Hydrolase	3.4.21.4	0.77
pdb_00001mq5	1	A	P00742	COAGULATION F/	Hydrolase	3.4.21.6	0.77
pdb_00001mtu	1	A	P00760	TRYPSIN	Hydrolase	3.4.21.4	0.77
pdb_00001v2n	1	A	P00760	Trypsin	Hydrolase	3.4.21.4	0.77
pdb_00003uy9	1	B	P00760	Cationic trypsin	Hydrolase	3.4.21.4	0.77
pdb_00008vgk	1	D	Q15661	Tryptase alpha/be	Hydrolase	3.4.21.59	0.77
pdb_00001k1l	1	A	P00760	TRYPSIN	Hydrolase	3.4.21.4	0.78
pdb_00002bm2	1	B	P20231	HUMAN BETA2 TI	Hydrolase	3.4.21.59	0.78
pdb_00003v13	1	A	P00760	Cationic trypsin	Hydrolase	3.4.21.4	0.78
pdb_00001k1m	1	A	P00760	TRYPSIN	Hydrolase	3.4.21.4	0.79
pdb_00002vvv	1	A	P00742	ACTIVATED FACT	Hydrolase	3.4.21.6	0.79
pdb_00003nk8	1	A	P00760	Cationic trypsin	Hydrolase	3.4.21.4	0.79
pdb_00006b6s	1	A	P00760	Cationic trypsin	Hydrolase	3.4.21.4	0.79
pdb_00001avx	1	A	P00761	TRYPSIN	Hydrolase	3.4.21.4	0.8
pdb_00001y5b	1	A	P00760	Trypsin, cationic	Hydrolase	3.4.21.4	0.8
pdb_00003v7t	1	C	P20231	TPSB2 protein	Hydrolase	3.4.21.59	0.8
pdb_00001bit	1	A	P35031	TRYPSIN	Hydrolase	3.4.21.4	0.81
pdb_00001c1s	1	A	P00760	TRYPSIN	Hydrolase	3.4.21.4	0.81
pdb_00001mtv	1	A	P00760	TRYPSIN	Hydrolase	3.4.21.4	0.81
pdb_00001xkb	2	B	P00742	BLOOD COAGULA	Hydrolase	3.4.21.6	0.81
pdb_00003hpt	2	B	P00742	Coagulation facto	Hydrolase	3.4.21.6	0.81
pdb_00003t27	1	A	P00760	Cationic trypsin	Hydrolase	3.4.21.4	0.81
pdb_00001c1p	1	A	P00760	TRYPSIN	Hydrolase	3.4.21.4	0.82
pdb_00001v2s	1	A	P00760	Trypsin	Hydrolase	3.4.21.4	0.82
pdb_00003rxv	1	A	P00760	Cationic trypsin	Hydrolase	3.4.21.4	0.82
pdb_00006o1f	1	A	Q15661	Tryptase alpha/be	Hydrolase	3.4.21.59	0.82
pdb_00006yiw	1	A	P00760	Cationic trypsin	Hydrolase	3.4.21.4	0.82
pdb_00001c5u	1	A	P00760	PROTEIN (TRYPSI	Hydrolase	3.4.21.4	0.83
pdb_00001mtw	1	A	P00760	TRYPSIN	Hydrolase	3.4.21.4	0.83
pdb_00001trn	1	A	P07477	TRYPSIN	Hydrolase	3.4.21.4	0.83
pdb_00003gy4	1	A	P00760	Cationic trypsin	Hydrolase	3.4.21.4	0.83
pdb_00006swv	1	A	P00760	Cationic trypsin	Hydrolase	3.4.21.4	0.83
pdb_00007e50	2	B	P00747	Plasminogen	Hydrolase	3.4.21.7	0.83

PDB ID	Entity ID	Chain ID(s)	UniProt ID(s)	Protein(s)	EC Class	EC Number(s)	RMSD
pdb_00009cm9	2	D	P00742	Coagulation facto	Hydrolase	3.4.21.6	0.83
pdb_00001a5h	2	D	P00750	TISSUE PLASMIN	Hydrolase	3.4.21.68	0.84
pdb_00001gj7	2	B	P00749	UROKINASE-TYPE	Hydrolase	3.4.21.73	0.84
pdb_00001rtf	2	B	P00750	TWO CHAIN TISS	Hydrolase	3.4.21.68	0.84
pdb_00003e0n	1	A	Q16651	Prostasin heavy c	Hydrolase	3.4.21	0.84
pdb_00003rxe	1	A	P00760	Cationic trypsin	Hydrolase	3.4.21.4	0.84
pdb_00006qff	1	B	Q92876	Kallikrein-6	Hydrolase	3.4.21	0.84
pdb_00006zfj	1	A	P00760	Cationic trypsin	Hydrolase	3.4.21.4	0.84
pdb_00008vgh	1	A	Q15661	Tryptase alpha/be	Hydrolase	3.4.21.59	0.84
pdb_00001gjc	2	B	P00749	UROKINASE-TYPE	Hydrolase	3.4.21.73	0.85
pdb_00001qb1	1	A	P00760	PROTEIN (TRYPSI	Hydrolase	3.4.21.4	0.85
pdb_00001qb6	1	A	P00760	PROTEIN (TRYPSI	Hydrolase	3.4.21.4	0.85
pdb_00001qbn	1	A	P00760	PROTEIN (TRYPSI	Hydrolase	3.4.21.4	0.85
pdb_00001v2l	1	A	P00760	Trypsin	Hydrolase	3.4.21.4	0.85
pdb_00003atl	1	A	P00760	Cationic trypsin	Hydrolase	3.4.21.4	0.85
pdb_00004yta	1	A	P00760	Cationic trypsin	Hydrolase	3.4.21.4	0.85
pdb_00001c5z	2	B	P00749	PROTEIN (UROKII	Hydrolase	3.4.21.73	0.86
pdb_00001gjb	2	B	P00749	UROKINASE-TYPE	Hydrolase	3.4.21.73	0.86
pdb_00001k1j	1	A	P00760	TRYPSIN	Hydrolase	3.4.21.4	0.86
pdb_00001o3p	2	B	P00749	Urokinase-type pl	Hydrolase	3.4.21.73	0.86
pdb_00001tld	1	A	P00760	BETA-TRYPSIN	Hydrolase	3.4.21.4	0.86
pdb_00003rxb	1	A	P00760	Cationic trypsin	Hydrolase	3.4.21.4	0.86
pdb_00006yiv	1	A	P00760	Cationic trypsin	Hydrolase	3.4.21.4	0.86
pdb_00006yzc	1	A	P00760	Cationic trypsin	Hydrolase	3.4.21.4	0.86
pdb_00001fax	1	A	P00742	FACTOR XA	Hydrolase	3.4.21.6	0.87
pdb_00001qb9	1	A	P00760	PROTEIN (TRYPSI	Hydrolase	3.4.21.4	0.87
pdb_00002boh	2	B	P00742	COAGULATION FA	Hydrolase	3.4.21.6	0.87
pdb_00002vvc	1	B	P00742	ACTIVATED FACT	Hydrolase	3.4.21.6	0.87
pdb_00003q00	1	A	P00760	Cationic trypsin	Hydrolase	3.4.21.4	0.87
pdb_00006zfk	1	A	P00760	Cationic trypsin	Hydrolase	3.4.21.4	0.87
pdb_00001c5t	1	A	P00760	PROTEIN (TRYPSI	Hydrolase	3.4.21.4	0.88
pdb_00001c5w	2	B	P00749	PROTEIN (UROKII	Hydrolase	3.4.21.73	0.88
pdb_00001o5b	2	B	P00749	Urokinase-type pl	Hydrolase	3.4.21.73	0.88
pdb_00001w11	1	A	P00749	UROKINASE-TYPE	Hydrolase	3.4.21.73	0.88
pdb_00006est	1	A	P00772	PORCINE PANCRI	Hydrolase	3.4.21.36	0.88
pdb_00001c5x	2	B	P00749	PROTEIN (UROKII	Hydrolase	3.4.21.73	0.89
pdb_00001c5y	2	B	P00749	PROTEIN (UROKII	Hydrolase	3.4.21.73	0.89
pdb_00001mq6	1	A	P00742	COAGULATION FA	Hydrolase	3.4.21.6	0.89

PDB ID	Entity ID	Chain ID(s)	UniProt ID(s)	Protein(s)	EC Class	EC Number(s)	RMSD
pdb_00003pm3	1	A	P00760	Cationic trypsin	Hydrolase	3.4.21.4	0.9
pdb_00003pmj	1	A	P00760	Cationic trypsin	Hydrolase	3.4.21.4	0.9
pdb_00003rxd	1	A	P00760	Cationic trypsin	Hydrolase	3.4.21.4	0.9
pdb_00003vfe	1	A	Q92876	Kallikrein-6	Hydrolase	3.4.21	0.9
pdb_00001qbo	1	A	P00760	PROTEIN (TRYPSI	Hydrolase	3.4.21.4	0.91
pdb_00001tpp	1	A	P00760	BETA-TRYPSIN	Hydrolase	3.4.21.4	0.91
pdb_00003kqd	1	A	P00742	factor Xa heavy cl	Hydrolase	3.4.21.6	0.91
pdb_00003rxu	1	A	P00760	Cationic trypsin	Hydrolase	3.4.21.4	0.91
pdb_00003uqv	1	A	P00760	Cationic trypsin	Hydrolase	3.4.21.4	0.91
pdb_00005mnn	1	A	P00760	Cationic trypsin	Hydrolase	3.4.21.4	0.91
pdb_00001ezq	1	A	P00742	COAGULATION F/	Hydrolase	3.4.21.6	0.92
pdb_00001gj9	2	B	P00749	UROKINASE-TYPE	Hydrolase	3.4.21.73	0.92
pdb_00001tx8	1	A	P00760	Trypsinogen	Hydrolase	3.4.21.4	0.92
pdb_00003k9x	2	B	P00742	PROTEIN (Coagul	Hydrolase	3.4.21.6	0.92
pdb_00003pyh	1	A	P00760	Cationic trypsin	Hydrolase	3.4.21.4	0.92
pdb_00003rxq	1	A	P00760	Cationic trypsin	Hydrolase	3.4.21.4	0.92
pdb_00004yzu	1	A	P00740	Coagulation facto	Hydrolase	3.4.21.22	0.92
pdb_00006t9v	1	A	P00760	Cationic Trypsin	Hydrolase	3.4.21.4	0.92
pdb_00001c5r	1	A	P00760	PROTEIN (TRYPSI	Hydrolase	3.4.21.4	0.93
pdb_00001jir	1	A	P00760	beta-Trypsin	Hydrolase	3.4.21.4	0.93
pdb_00008eok	6	F	P00742	Activated factor X	Hydrolase	3.4.21.6	0.93
pdb_00008vgj	1	C	Q15661	Tryptase alpha/be	Hydrolase	3.4.21.59	0.93
pdb_00001g3e	1	A	P00760	BETA-TRYPSIN	Hydrolase	3.4.21.4	0.94
pdb_00001tpo	1	A	P00760	BETA-TRYPSIN	Hydrolase	3.4.21.4	0.94
pdb_00001vja	1	A	P00749	plasminogen acti	Hydrolase	3.4.21.73	0.94
pdb_00001z6e	1	A	P00742	Coagulation facto	Hydrolase	3.4.21.6	0.94
pdb_00002p3u	2	B	P00742	Coagulation facto	Hydrolase	3.4.21.6	0.94
pdb_00002ptn	1	A	P00760	TRYPSIN	Hydrolase	3.4.21.4	0.94
pdb_00003rxa	1	A	P00760	Cationic trypsin	Hydrolase	3.4.21.4	0.94
pdb_00006qfe	1	B	Q9Y337	Kallikrein-5	Hydrolase	3.4.21	0.94
pdb_00002xbw	1	A	P00742	ACTIVATED FACT	Hydrolase	3.4.21.6	0.95
pdb_00004a7i	2	B	P00742	ACTIVATED FACT	Hydrolase	3.4.21.6	0.95
pdb_00004fud	1	A	P00749	Urokinase-type pl	Hydrolase	3.4.21.73	0.95
pdb_00004z0k	1	A	P00740	Coagulation facto	Hydrolase	3.4.21.22	0.95
pdb_00005tno	1	A	P00740	Coagulation facto	Hydrolase	3.4.21.22	0.95
pdb_00008r8d	1	A	P00748	Coagulation facto	Hydrolase	3.4.21.38	0.95
pdb_00009kk5	1	A	P00760	Cationic trypsin	Hydrolase	3.4.21.4	0.95
pdb_00001o37	1	A	P00760	BETA-TRYPSIN	Hydrolase	3.4.21.4	0.96

PDB ID	Entity ID	Chain ID(s)	UniProt ID(s)	Protein(s)	EC Class	EC Number(s)	RMSD
pdb_00001sqo	1	A	P00749	Urokinase-type pl	Hydrolase	3.4.21.73	0.96
pdb_00002vin	1	A	P00749	UROKINASE-TYPE	Hydrolase	3.4.21.73	0.96
pdb_00002wpi	3	C	P00740	COAGULATION FA	Hydrolase	3.4.21.22	0.96
pdb_00002zps	1	A	B3Y8K6	Anionic trypsin	Hydrolase	3.4.21.4	0.96
pdb_00003m37	1	A	P00742	Coagulation facto	Hydrolase	3.4.21.6	0.96
pdb_00003rxr	1	A	P00760	Cationic trypsin	Hydrolase	3.4.21.4	0.96
pdb_00005jb9	2	B	P00740	Coagulation facto	Hydrolase	3.4.21.22	0.96
pdb_00005jyi	1	A	P00760	Cationic trypsin	Hydrolase	3.4.21.4	0.96
pdb_00006qf7	2	B	P00748	Coagulation facto	Hydrolase	3.4.21.38	0.96
pdb_00001a0l	1	B	P20231	BETA-TRYPTASE	Hydrolase	3.4.21.59	0.97
pdb_00001mbq	1	A	Q8AV11	Trypsin	Hydrolase	3.4.21.4	0.97
pdb_00001nfu	1	A	P00742	COAGULATION FA	Hydrolase	3.4.21.6	0.97
pdb_00002fzz	1	A	P00742	Coagulation facto	Hydrolase	3.4.21.6	0.97
pdb_00002r0l	3	C	Q04756	Hepatocyte growt	Hydrolase	3.4.21	0.97
pdb_00003ens	2	D	P00742	Activated factor X	Hydrolase	3.4.21.6	0.97
pdb_00001gi8	2	B	P00749	UROKINASE-TYPE	Hydrolase	3.4.21.73	0.98
pdb_00001k1n	1	A	P00760	TRYPSIN	Hydrolase	3.4.21.4	0.98
pdb_00001utl	1	A	P35031	TRYPSIN I	Hydrolase	3.4.21.4	0.98
pdb_00001ybw	1	A	Q04756	Hepatocyte growt	Hydrolase	3.4.21	0.98
pdb_00002ei7	1	A	P00742	Coagulation facto	Hydrolase	3.4.21.6	0.98
pdb_00002zeb	1	C	Q15661	Tryptase beta 2	Hydrolase	3.4.21.59	0.98
pdb_00003bn9	1	D	Q9Y5Y6	Membrane-type s	Hydrolase	3.4.21.109	0.98
pdb_00004fug	1	A	P00749	Urokinase-type pl	Hydrolase	3.4.21.73	0.98
pdb_00006qh9	1	A	Q92876	Kallikrein-6	Hydrolase	3.4.21	0.98
pdb_00008vgi	1	A	Q15661	Tryptase alpha/be	Hydrolase	3.4.21.59	0.98
pdb_00002gv7	1	A	Q9Y5Y6	Suppressor of tun	Hydrolase	3.4.21.109	0.99
pdb_00003cen	1	A	P00742	COAGULATION FA	Hydrolase	3.4.21.6	0.99
pdb_00003m36	1	A	P00742	Coagulation facto	Hydrolase	3.4.21.6	0.99
pdb_00003nps	1	A	Q9Y5Y6	Suppressor of tun	Hydrolase	3.4.21.109	0.99
pdb_00005tnt	1	A	P00740	Coagulation facto	Hydrolase	3.4.21.22	0.99
pdb_00001ksn	1	A	P00742	COAGULATION FA	Hydrolase	3.4.21.6	1
pdb_00001owh	1	A	P00749	Urokinase-type pl	Hydrolase	3.4.21.73	1
pdb_00002p94	1	A	P00742	Factor Xa	Hydrolase	3.4.21.6	1
pdb_00003tk6	1	A	P00742	Factor X heavy ch	Hydrolase	3.4.21.6	1
pdb_00003w94	1	A	A4UWM5	Enteropeptidase-1			1
pdb_00005dk1	1	A	P24664	Trypsin	Hydrolase	3.4.21.4	1
pdb_00005jba	2	B	P00740	Coagulation facto	Hydrolase	3.4.21.22	1
pdb_00008h3u	2	B	P98073	Enteropeptidase c	Hydrolase	3.4.21.9	1

PDB ID	Entity ID	Chain ID(s)	UniProt ID(s)	Protein(s)	EC Class	EC Number(s)	RMSD
pdb_00001c5s	1	A	P00760	PROTEIN (TRYPSI	Hydrolase	3.4.21.4	1.01
pdb_00001f0s	1	A	P00742	COAGULATION FA	Hydrolase	3.4.21.6	1.01
pdb_00001f0u	1	A	P00760	TRYPSIN	Hydrolase	3.4.21.4	1.01
pdb_00001fjs	1	A	P00742	COAGULATION FA	Hydrolase	3.4.21.6	1.01
pdb_00001nfx	1	A	P00742	COAGULATION FA	Hydrolase	3.4.21.6	1.01
pdb_00001o2u	1	A	P00760	BETA-TRYPSIN	Hydrolase	3.4.21.4	1.01
pdb_00001o2w	1	A	P00760	BETA-TRYPSIN	Hydrolase	3.4.21.4	1.01
pdb_00001owk	1	A	P00749	Urokinase-type pl	Hydrolase	3.4.21.73	1.01
pdb_00001s82	1	A	P00761	TRYPSIN	Hydrolase	3.4.21.4	1.01
pdb_00001y3y	1	A	P00760	Trypsin, cationic	Hydrolase	3.4.21.4	1.01
pdb_00002p95	1	A	P00742	Factor Xa	Hydrolase	3.4.21.6	1.01
pdb_00003iit	1	A	P00742	Activated factor X	Hydrolase	3.4.21.6	1.01
pdb_00003kqc	1	A	P00742	factor Xa heavy cl	Hydrolase	3.4.21.6	1.01
pdb_00003liw	1	A	P00742	Activated factor X	Hydrolase	3.4.21.6	1.01
pdb_00004os5	1	A	P00749	Urokinase-type pl	Hydrolase	3.4.21.73	1.01
pdb_00006skd	1	A	Q92876	Kallikrein-6	Hydrolase	3.4.21	1.01
pdb_00009awi	1	A	P00760	Cationic trypsin	Hydrolase	3.4.21.4	1.01
pdb_00001g2l	1	A	P00742	COAGULATION FA	Hydrolase	3.4.21.6	1.02
pdb_00001gj8	2	B	P00749	UROKINASE-TYPE	Hydrolase	3.4.21.73	1.02
pdb_00001gjd	2	B	P00749	UROKINASE-TYPE	Hydrolase	3.4.21.73	1.02
pdb_00001max	1	A	P00760	BETA-TRYPSIN	Hydrolase	3.4.21.4	1.02
pdb_00001o2h	1	A	P00760	BETA-TRYPSIN	Hydrolase	3.4.21.4	1.02
pdb_00001o5a	2	B	P00749	Urokinase-type pl	Hydrolase	3.4.21.73	1.02
pdb_00002vio	1	A	P00749	UROKINASE-TYPE	Hydrolase	3.4.21.73	1.02
pdb_00002vnt	1	A	P00749	UROKINASE-TYPE	Hydrolase	3.4.21.73	1.02
pdb_00002vwo	1	A	P00742	ACTIVATED FACT	Hydrolase	3.4.21.6	1.02
pdb_00002xbv	1	A	P00742	ACTIVATED FACT	Hydrolase	3.4.21.6	1.02
pdb_00002za5	1	B	P20231	Tryptase beta 2	Hydrolase	3.4.21.59	1.02
pdb_00003ffg	1	A	P00742	Coagulation facto	Hydrolase	3.4.21.6	1.02
pdb_00004os7	1	A	P00749	Urokinase-type pl	Hydrolase	3.4.21.73	1.02
pdb_00005egm	1	A	P00740	Coagulation facto	Hydrolase	3.4.21.22	1.02
pdb_00006qfh	1	A	Q92876	Kallikrein-6	Hydrolase	3.4.21	1.02
pdb_00001gja	2	B	P00749	UROKINASE-TYPE	Hydrolase	3.4.21.73	1.03
pdb_00001nfw	1	A	P00742	COAGULATION FA	Hydrolase	3.4.21.6	1.03
pdb_00001o2v	1	A	P00760	BETA-TRYPSIN	Hydrolase	3.4.21.4	1.03
pdb_00001o2x	1	A	P00760	BETA-TRYPSIN	Hydrolase	3.4.21.4	1.03
pdb_00001o2y	1	A	P00760	BETA-TRYPSIN	Hydrolase	3.4.21.4	1.03
pdb_00001o31	1	A	P00760	BETA-TRYPSIN	Hydrolase	3.4.21.4	1.03

PDB ID	Entity ID	Chain ID(s)	UniProt ID(s)	Protein(s)	EC Class	EC Number(s)	RMSD
pdb_00001owe	1	A	P00749	Urokinase-type pl:	Hydrolase	3.4.21.73	1.03
pdb_00002gv6	1	A	Q9Y5Y6	Suppressor of tur	Hydrolase	3.4.21.109	1.03
pdb_00002j95	1	A	P00742	ACTIVATED FACT	Hydrolase	3.4.21.6	1.03
pdb_00002vwn	1	A	P00742	ACTIVATED FACT	Hydrolase	3.4.21.6	1.03
pdb_00002w26	1	A	P00742	ACTIVATED FACT	Hydrolase	3.4.21.6	1.03
pdb_00003lc3	1	C	P00740	Coagulation facto	Hydrolase	3.4.21.22	1.03
pdb_00004fuc	1	A	P00749	Urokinase-type pl:	Hydrolase	3.4.21.73	1.03
pdb_00004y79	1	A	P00742	Coagulation facto	Hydrolase	3.4.21.6	1.03
pdb_00007brx	1	A	P00760	Cationic trypsin	Hydrolase	3.4.21.4	1.03
pdb_00009kk4	1	A	P00760	Cationic trypsin	Hydrolase	3.4.21.4	1.03
pdb_00009p7b	1	A	P00760	Pretrypsinogen I	Hydrolase	3.4.21.4	1.03
pdb_00009r8r	1	A	P00742	Coagulation facto	Hydrolase	3.4.21.6	1.03
pdb_00001amh	1	B	P00763	ANIONIC TRYPSIN	Hydrolase	3.4.21.4	1.04
pdb_00001c1r	1	A	P00760	TRYPSIN	Hydrolase	3.4.21.4	1.04
pdb_00001gi7	2	B	P00749	UROKINASE-TYPE	Hydrolase	3.4.21.73	1.04
pdb_00001lpk	2	B	P00742	Blood coagulation	Hydrolase	3.4.21.6	1.04
pdb_00001lpz	2	B	P00742	Blood coagulation	Hydrolase	3.4.21.6	1.04
pdb_00001o2j	1	A	P00760	BETA-TRYPSIN	Hydrolase	3.4.21.4	1.04
pdb_00001o33	1	A	P00760	BETA-TRYPSIN	Hydrolase	3.4.21.4	1.04
pdb_00001tab	1	A	P00760	TRYPSIN	Hydrolase	3.4.21.4	1.04
pdb_00002phb	1	A	P00742	Coagulation facto	Hydrolase	3.4.21.6	1.04
pdb_00002pr3	1	A	P00742	COAGULATION F/	Hydrolase	3.4.21.6	1.04
pdb_00002uwo	1	A	P00742	COAGULATION F/	Hydrolase	3.4.21.6	1.04
pdb_00002v35	1	A	P00772	ELASTASE-1	Hydrolase	3.4.21.36	1.04
pdb_00002zec	1	C	Q15661	Tryptase beta 2	Hydrolase	3.4.21.59	1.04
pdb_00003kqb	1	A	P00742	factor Xa heavy cl	Hydrolase	3.4.21.6	1.04
pdb_00003kqe	1	A	P00742	factor Xa heavy cl	Hydrolase	3.4.21.6	1.04
pdb_00004fuf	1	A	P00749	Urokinase-type pl:	Hydrolase	3.4.21.73	1.04
pdb_00004os4	1	A	P00749	Urokinase-type pl:	Hydrolase	3.4.21.73	1.04
pdb_00006skb	1	A	Q92876	Kallikrein-6	Hydrolase	3.4.21	1.04
pdb_00006x5l	1	A	P00740	Coagulation facto	Hydrolase	3.4.21.22	1.04
pdb_00008xnj	1	A	P00760	Cationic trypsin	Hydrolase	3.4.21.4	1.04
pdb_00001f92	1	A	P00749	UROKINASE-TYPE	Hydrolase	3.4.21.73	1.05
pdb_00001lpg	2	B	P00742	Blood coagulation	Hydrolase	3.4.21.6	1.05
pdb_00001o3d	1	A	P00760	BETA-TRYPSIN	Hydrolase	3.4.21.4	1.05
pdb_00001owi	1	A	P00749	Urokinase-type pl:	Hydrolase	3.4.21.73	1.05
pdb_00001sqt	1	A	P00749	Urokinase-type pl:	Hydrolase	3.4.21.73	1.05
pdb_00002g00	1	A	P00742	Coagulation facto	Hydrolase	3.4.21.6	1.05

PDB ID	Entity ID	Chain ID(s)	UniProt ID(s)	Protein(s)	EC Class	EC Number(s)	RMSD
pdb_00002w3k	1	A	P00742	COAGULATION FA	Hydrolase	3.4.21.6	1.05
pdb_00003atk	1	A	P00760	Cationic trypsin	Hydrolase	3.4.21.4	1.05
pdb_00003kl6	1	A	P00742	Coagulation Facto	Hydrolase	3.4.21.6	1.05
pdb_00003v12	1	A	P00760	Cationic trypsin	Hydrolase	3.4.21.4	1.05
pdb_00004bti	2	B	P00742	COAGULATION FA	Hydrolase	3.4.21.6	1.05
pdb_00004fu8	1	A	P00749	Urokinase-type pla	Hydrolase	3.4.21.73	1.05
pdb_00004zae	1	A	P00740	Coagulation facto	Hydrolase	3.4.21.22	1.05
pdb_00009awn	1	A	P00760	Cationic trypsin	Hydrolase	3.4.21.4	1.05
pdb_00001c2g	1	A	P00760	TRYPSIN	Hydrolase	3.4.21.4	1.06
pdb_00001f0r	1	A	P00742	COAGULATION FA	Hydrolase	3.4.21.6	1.06
pdb_00001g3b	1	A	P00760	BETA-TRYPSIN	Hydrolase	3.4.21.4	1.06
pdb_00001o38	1	A	P00760	BETA-TRYPSIN	Hydrolase	3.4.21.4	1.06
pdb_00001o3l	1	A	P00760	BETA-TRYPSIN	Hydrolase	3.4.21.4	1.06
pdb_00001o3m	1	A	P00760	BETA-TRYPSIN	Hydrolase	3.4.21.4	1.06
pdb_00001o3n	1	A	P00760	BETA-TRYPSIN	Hydrolase	3.4.21.4	1.06
pdb_00002tbs	1	A	P35031	TRYPSIN	Hydrolase	3.4.21.4	1.06
pdb_00002w3i	1	A	P00742	COAGULATION FA	Hydrolase	3.4.21.6	1.06
pdb_00003q3k	1	A	P00742	Activated factor X	Hydrolase	3.4.21.6	1.06
pdb_00003uns	1	A	P00760	Cationic trypsin	Hydrolase	3.4.21.4	1.06
pdb_00003vpk	1	A	P00760	Cationic trypsin	Hydrolase	3.4.21.4	1.06
pdb_00004btu	2	B	P00742	COAGULATION FA	Hydrolase	3.4.21.6	1.06
pdb_00004fu7	1	A	P00749	Urokinase-type pla	Hydrolase	3.4.21.73	1.06
pdb_00004fub	1	A	P00749	Urokinase-type pla	Hydrolase	3.4.21.73	1.06
pdb_00004fue	1	A	P00749	Urokinase-type pla	Hydrolase	3.4.21.73	1.06
pdb_00005ce1	1	A	P05981	Serine protease h	Hydrolase	3.4.21.106	1.06
pdb_00005eg4	1	A	P00760	Cationic trypsin	Hydrolase	3.4.21.4	1.06
pdb_00005za7	1	A	P00749	Urokinase-type pla	Hydrolase	3.4.21.73	1.06
pdb_00001c2e	1	A	P00760	TRYPSIN	Hydrolase	3.4.21.4	1.07
pdb_00001gi5	1	A	P00760	BETA-TRYPSIN	Hydrolase	3.4.21.4	1.07
pdb_00001gi9	2	B	P00749	UROKINASE-TYPE	Hydrolase	3.4.21.73	1.07
pdb_00001o2i	1	A	P00760	BETA-TRYPSIN	Hydrolase	3.4.21.4	1.07
pdb_00001o2o	1	A	P00760	BETA-TRYPSIN	Hydrolase	3.4.21.4	1.07
pdb_00001o2p	1	A	P00760	BETA-TRYPSIN	Hydrolase	3.4.21.4	1.07
pdb_00001o2z	1	A	P00760	BETA-TRYPSIN	Hydrolase	3.4.21.4	1.07
pdb_00001o39	1	A	P00760	BETA-TRYPSIN	Hydrolase	3.4.21.4	1.07
pdb_00001o5c	2	B	P00749	Urokinase-type pla	Hydrolase	3.4.21.73	1.07
pdb_00001y3u	1	A	P00760	Trypsin, cationic	Hydrolase	3.4.21.4	1.07
pdb_00003cs7	1	A	P00742	Coagulation facto	Hydrolase	3.4.21.6	1.07

PDB ID	Entity ID	Chain ID(s)	UniProt ID(s)	Protein(s)	EC Class	EC Number(s)	RMSD
pdb_00003fvf	1	A	Q16651	Prostasin	Hydrolase	3.4.21	1.07
pdb_00003k6y	1	A	P9WHR9	POSSIBLE MEMB	Hydrolase	3.4.21	1.07
pdb_00004btt	2	B	P00742	COAGULATION FA	Hydrolase	3.4.21.6	1.07
pdb_00004fuh	1	A	P00749	Urokinase-type pl	Hydrolase	3.4.21.73	1.07
pdb_00001gi2	1	A	P00760	BETA-TRYPSIN	Hydrolase	3.4.21.4	1.08
pdb_00001kig	1	A	P00743	FACTOR XA	Hydrolase	3.4.21.6	1.08
pdb_00001nfy	1	A	P00742	Coagulation facto	Hydrolase	3.4.21.6	1.08
pdb_00001o2k	1	A	P00760	BETA-TRYPSIN	Hydrolase	3.4.21.4	1.08
pdb_00001o30	1	A	P00760	BETA-TRYPSIN	Hydrolase	3.4.21.4	1.08
pdb_00001o34	1	A	P00760	BETA-TRYPSIN	Hydrolase	3.4.21.4	1.08
pdb_00001owd	1	A	P00749	Urokinase-type pl	Hydrolase	3.4.21.73	1.08
pdb_00001p57	2	B	P05981	Serine protease h	Hydrolase	3.4.21.106	1.08
pdb_00001y5a	1	A	P00760	Trypsin, cationic	Hydrolase	3.4.21.4	1.08
pdb_00002bmg	2	B	P00742	COAGULATION FA	Hydrolase	3.4.21.6	1.08
pdb_00002ei8	1	A	P00742	Coagulation facto	Hydrolase	3.4.21.6	1.08
pdb_00002p93	1	A	P00742	Factor Xa	Hydrolase	3.4.21.6	1.08
pdb_00002q1j	1	A	P00742	Activated factor X	Hydrolase	3.4.21.6	1.08
pdb_00002vvu	1	A	P00742	ACTIVATED FACT	Hydrolase	3.4.21.6	1.08
pdb_00004mny	1	B	P00749	Urokinase-type pl	Hydrolase	3.4.21.73	1.08
pdb_00006ql0	1	A	P00760	Cationic trypsin	Hydrolase	3.4.21.4	1.08
pdb_00001c2d	1	A	P00760	TRYPSIN	Hydrolase	3.4.21.4	1.09
pdb_00001g36	1	A	P00760	TRYPSINOGEN, C	Hydrolase	3.4.21.4	1.09
pdb_00001gi0	1	A	P00760	BETA-TRYPSIN	Hydrolase	3.4.21.4	1.09
pdb_00001gj6	1	A	P00760	BETA-TRYPSIN	Hydrolase	3.4.21.4	1.09
pdb_00001o2r	1	A	P00760	BETA-TRYPSIN	Hydrolase	3.4.21.4	1.09
pdb_00001o36	1	A	P00760	BETA-TRYPSIN	Hydrolase	3.4.21.4	1.09
pdb_00001o3b	1	A	P00760	BETA-TRYPSIN	Hydrolase	3.4.21.4	1.09
pdb_00001rxp	1	A	P00760	TRYPSIN	Hydrolase	3.4.21.4	1.09
pdb_00001utj	1	A	P35031	TRYPSIN I	Hydrolase	3.4.21.4	1.09
pdb_00002ei6	1	A	P00742	Coagulation facto	Hydrolase	3.4.21.6	1.09
pdb_00002j94	1	A	P00742	COAGULATION FA	Hydrolase	3.4.21.6	1.09
pdb_00002vip	1	A	P00749	UROKINASE-TYPE	Hydrolase	3.4.21.73	1.09
pdb_00002viv	1	A	P00749	UROKINASE-TYPE	Hydrolase	3.4.21.73	1.09
pdb_00003gy6	1	A	P00760	Cationic trypsin	Hydrolase	3.4.21.4	1.09
pdb_00003uwi	1	A	P00760	Cationic trypsin	Hydrolase	3.4.21.4	1.09
pdb_00003v0x	1	A	P00760	Cationic trypsin	Hydrolase	3.4.21.4	1.09
pdb_00004fui	1	A	P00749	Urokinase-type pl	Hydrolase	3.4.21.73	1.09
pdb_00004mnx	1	A	P00749	Urokinase-type pl	Hydrolase	3.4.21.73	1.09

PDB ID	Entity ID	Chain ID(s)	UniProt ID(s)	Protein(s)	EC Class	EC Number(s)	RMSD
pdb_00004x1n	2	B	P00749	Urokinase-type pl	Hydrolase	3.4.21.73	1.09
pdb_00004zhl	1	A	P00749	Urokinase-type pl	Hydrolase	3.4.21.73	1.09
pdb_00005ptp	1	A	P00760	BETA TRYPSIN	Hydrolase	3.4.21.4	1.09
pdb_00005wxr	1	A	P00749	Urokinase-type pl	Hydrolase	3.4.21.73	1.09
pdb_00006b74	2	B	P00748	Coagulation facto	Hydrolase	3.4.21.38	1.09
pdb_00006qfg	1	A	Q92876	Kallikrein-6	Hydrolase	3.4.21	1.09
pdb_00006x5p	1	A	P00740	Coagulation facto	Hydrolase	3.4.21.22	1.09
pdb_00007bry	1	A	P00760	Cationic trypsin	Hydrolase	3.4.21.4	1.09
pdb_00007bs5	1	A	P00760	Cationic trypsin	Hydrolase	3.4.21.4	1.09
pdb_00007wb9	1	A	P00760	Cationic trypsin	Hydrolase	3.4.21.4	1.09
pdb_00001c2f	1	A	P00760	TRYPSIN	Hydrolase	3.4.21.4	1.1
pdb_00001f0t	1	A	P00760	TRYPSIN	Hydrolase	3.4.21.4	1.1
pdb_00001gi6	1	A	P00760	BETA-TRYPSIN	Hydrolase	3.4.21.4	1.1
pdb_00001o2m	1	A	P00760	BETA-TRYPSIN	Hydrolase	3.4.21.4	1.1
pdb_00001o3c	1	A	P00760	BETA-TRYPSIN	Hydrolase	3.4.21.4	1.1
pdb_00001o3h	1	A	P00760	BETA-TRYPSIN	Hydrolase	3.4.21.4	1.1
pdb_00001o3j	1	A	P00760	BETA-TRYPSIN	Hydrolase	3.4.21.4	1.1
pdb_00001o3k	1	A	P00760	BETA-TRYPSIN	Hydrolase	3.4.21.4	1.1
pdb_00001o3o	1	A	P00760	BETA-TRYPSIN	Hydrolase	3.4.21.4	1.1
pdb_00001o5f	2	B	P05981	Serine protease h	Hydrolase	3.4.21.106	1.1
pdb_00001utm	1	A	P35031	TRYPSIN I	Hydrolase	3.4.21.4	1.1
pdb_00002bqw	2	B	P00742	FACTOR XA	Hydrolase	3.4.21.6	1.1
pdb_00002vwl	1	A	P00742	ACTIVATED FACT	Hydrolase	3.4.21.6	1.1
pdb_00002xbx	1	A	P00742	ACTIVATED FACT	Hydrolase	3.4.21.6	1.1
pdb_00002xc0	1	A	P00742	ACTIVATED FACT	Hydrolase	3.4.21.6	1.1
pdb_00002xc4	1	A	P00742	ACTIVATED FACT	Hydrolase	3.4.21.6	1.1
pdb_00002xc5	1	A	P00742	ACTIVATED FACT	Hydrolase	3.4.21.6	1.1
pdb_00003gy8	1	A	P00760	Cationic trypsin	Hydrolase	3.4.21.4	1.1
pdb_00003rxi	1	A	P00760	Cationic trypsin	Hydrolase	3.4.21.4	1.1
pdb_00003rxm	1	A	P00760	Cationic trypsin	Hydrolase	3.4.21.4	1.1
pdb_00003so3	1	A	Q9Y5Y6	Suppressor of tur	Hydrolase	3.4.21.109	1.1
pdb_00004x1s	2	B	P00749	Urokinase-type pl	Hydrolase	3.4.21.73	1.1
pdb_00005gxp	1	B	P00760	Cationic trypsin	Hydrolase	3.4.21.4	1.1
pdb_00006ag7	1	A	P00749	Urokinase-type pl	Hydrolase	3.4.21.73	1.1
pdb_00006t0p	1	A	P00760	Cationic Trypsin	Hydrolase	3.4.21.4	1.1
pdb_00001ghz	1	A	P00760	BETA-TRYPSIN	Hydrolase	3.4.21.4	1.11
pdb_00001gi1	1	A	P00760	BETA-TRYPSIN	Hydrolase	3.4.21.4	1.11
pdb_00001o2l	1	A	P00760	BETA-TRYPSIN	Hydrolase	3.4.21.4	1.11

PDB ID	Entity ID	Chain ID(s)	UniProt ID(s)	Protein(s)	EC Class	EC Number(s)	RMSD
pdb_00001o2q	1	A	P00760	BETA-TRYPSIN	Hydrolase	3.4.21.4	1.11
pdb_00001o2t	1	A	P00760	BETA-TRYPSIN	Hydrolase	3.4.21.4	1.11
pdb_00001o3a	1	A	P00760	BETA-TRYPSIN	Hydrolase	3.4.21.4	1.11
pdb_00001o3i	1	A	P00760	BETA-TRYPSIN	Hydrolase	3.4.21.4	1.11
pdb_00001s84	1	A	P00761	TRYPSIN	Hydrolase	3.4.21.4	1.11
pdb_00001s85	1	A	P00761	TRYPSIN	Hydrolase	3.4.21.4	1.11
pdb_00001u6q	1	A	P00749	Urokinase-type pl	Hydrolase	3.4.21.73	1.11
pdb_00001wu1	1	A	P00742	Coagulation facto	Hydrolase	3.4.21.6	1.11
pdb_00002p16	1	A	P00742	Coagulation facto	Hydrolase	3.4.21.6	1.11
pdb_00002vwm	1	A	P00742	ACTIVATED FACT	Hydrolase	3.4.21.6	1.11
pdb_00003rxk	1	A	P00760	Cationic trypsin	Hydrolase	3.4.21.4	1.11
pdb_00003upe	1	A	P00760	Cationic trypsin	Hydrolase	3.4.21.4	1.11
pdb_00005wxq	1	A	P00749	Urokinase-type pl	Hydrolase	3.4.21.73	1.11
pdb_00005zaj	1	A	P00749	Urokinase-type pl	Hydrolase	3.4.21.73	1.11
pdb_00006jyp	1	A	P00749	Urokinase-type pl	Hydrolase	3.4.21.73	1.11
pdb_00006jyq	1	A	P00749	Urokinase-type pl	Hydrolase	3.4.21.73	1.11
pdb_00006qhc	1	A	Q92876	Kallikrein-6	Hydrolase	3.4.21	1.11
pdb_00006x5j	1	A	P00740	Coagulation facto	Hydrolase	3.4.21.22	1.11
pdb_00008izh	1	A	P00760	Cationic trypsin	Hydrolase	3.4.21.4	1.11
pdb_00001and	1	A	P00763	ANIONIC TRYPSII	Hydrolase	3.4.21.4	1.12
pdb_00001o2s	1	A	P00760	BETA-TRYPSIN	Hydrolase	3.4.21.4	1.12
pdb_00001s6f	1	A	P00761	Trypsin	Hydrolase	3.4.21.4	1.12
pdb_00001yp9	1	A	P00760	Cationic trypsin	Hydrolase	3.4.21.4	1.12
pdb_00002d1j	1	A	P00742	Coagulation facto	Hydrolase	3.4.21.6	1.12
pdb_00003lc5	1	A	P00740	Coagulation facto	Hydrolase	3.4.21.22	1.12
pdb_00004ab8	1	A	P00760	CATIONIC TRYPSI	Hydrolase	3.4.21.4	1.12
pdb_00004o97	1	A	Q9Y5Y6	Suppressor of tur	Hydrolase	3.4.21.109	1.12
pdb_00005za9	1	A	P00749	Urokinase-type pl	Hydrolase	3.4.21.73	1.12
pdb_00006l04	1	A	P00749	Urokinase-type pl	Hydrolase	3.4.21.73	1.12
pdb_00006sy3	1	A	P00760	Cationic Trypsin	Hydrolase	3.4.21.4	1.12
pdb_00007bs4	1	A	P00760	Cationic trypsin	Hydrolase	3.4.21.4	1.12
pdb_00007bs6	1	A	P00760	Cationic trypsin	Hydrolase	3.4.21.4	1.12
pdb_00007bs9	1	A	P00760	Cationic trypsin	Hydrolase	3.4.21.4	1.12
pdb_00001gi3	1	A	P00760	BETA-TRYPSIN	Hydrolase	3.4.21.4	1.13
pdb_00001o2n	1	A	P00760	BETA-TRYPSIN	Hydrolase	3.4.21.4	1.13
pdb_00001o35	1	A	P00760	BETA-TRYPSIN	Hydrolase	3.4.21.4	1.13
pdb_00001o3e	1	A	P00760	BETA-TRYPSIN	Hydrolase	3.4.21.4	1.13
pdb_00001o3f	1	A	P00760	BETA-TRYPSIN	Hydrolase	3.4.21.4	1.13

PDB ID	Entity ID	Chain ID(s)	UniProt ID(s)	Protein(s)	EC Class	EC Number(s)	RMSD
pdb_00001o3g	1	A	P00760	BETA-TRYPSIN	Hydrolase	3.4.21.4	1.13
pdb_00001o5e	2	B	P05981	Serine protease h	Hydrolase	3.4.21.106	1.13
pdb_00001w0z	1	A	P00749	UROKINASE-TYPE	Hydrolase	3.4.21.73	1.13
pdb_00001y3x	1	A	P00760	Trypsinogen, catio	Hydrolase	3.4.21.4	1.13
pdb_00002j2u	1	A	P00742	COAGULATION FA	Hydrolase	3.4.21.6	1.13
pdb_00002j34	1	A	P00742	ACTIVATED FACT	Hydrolase	3.4.21.6	1.13
pdb_00002ra0	1	A	P00742	Coagulation facto	Hydrolase	3.4.21.6	1.13
pdb_00002viw	1	A	P00749	UROKINASE-TYPE	Hydrolase	3.4.21.73	1.13
pdb_00003a83	1	A	P00760	Cationic trypsin	Hydrolase	3.4.21.4	1.13
pdb_00003rxh	1	A	P00760	Cationic trypsin	Hydrolase	3.4.21.4	1.13
pdb_00003rxl	1	A	P00760	Cationic trypsin	Hydrolase	3.4.21.4	1.13
pdb_00003rxo	1	A	P00760	Cationic trypsin	Hydrolase	3.4.21.4	1.13
pdb_00004fu9	1	A	P00749	Urokinase-type pl	Hydrolase	3.4.21.73	1.13
pdb_00004jk6	1	A	P00749	Urokinase-type pl	Hydrolase	3.4.21.73	1.13
pdb_00004x0w	2	B	P00749	Urokinase-type pl	Hydrolase	3.4.21.73	1.13
pdb_00005yc7	1	A	P00749	Urokinase-type pl	Hydrolase	3.4.21.73	1.13
pdb_00006ag2	1	A	P00749	Urokinase-type pl	Hydrolase	3.4.21.73	1.13
pdb_00007zrr	1	A	P00749	Urokinase-type pl	Hydrolase	3.4.21.73	1.13
pdb_00008v1f	2	D	O15393	Transmembrane p	Hydrolase	3.4.21.122	1.13
pdb_00009bok	1	A	P00760	Cationic trypsin	Hydrolase	3.4.21.4	1.13
pdb_00001c5q	1	A	P00760	PROTEIN (TRYPS	Hydrolase	3.4.21.4	1.14
pdb_00001lqd	2	B	P00742	Blood coagulation	Hydrolase	3.4.21.6	1.14
pdb_00001ntp	1	A	P00760	BETA-TRYPSIN	Hydrolase	3.4.21.4	1.14
pdb_00001oyq	1	A	P00760	Trypsin, cationic	Hydrolase	3.4.21.4	1.14
pdb_00001sc8	1	A	P00749	plasminogen acti	Hydrolase	3.4.21.73	1.14
pdb_00001utp	1	A	P00760	TRYPSINOGEN	Hydrolase	3.4.21.4	1.14
pdb_00001utq	1	A	P00760	TRYPSINOGEN	Hydrolase	3.4.21.4	1.14
pdb_00002bq7	2	B	P00742	FACTOR XA	Hydrolase	3.4.21.6	1.14
pdb_00002xby	1	A	P00742	ACTIVATED FACT	Hydrolase	3.4.21.6	1.14
pdb_00003a88	1	A	P00760	Cationic trypsin	Hydrolase	3.4.21.4	1.14
pdb_00003a89	1	A	P00760	Cationic trypsin	Hydrolase	3.4.21.4	1.14
pdb_00003aas	1	A	P00760	Cationic trypsin	Hydrolase	3.4.21.4	1.14
pdb_00003dfl	1	A	Q16651	Prostasin	Hydrolase	3.4.21	1.14
pdb_00003qn7	1	A	P00749	Urokinase-type pl	Hydrolase	3.4.21.73	1.14
pdb_00003rxf	1	A	P00760	Cationic trypsin	Hydrolase	3.4.21.4	1.14
pdb_00003sw2	2	B	P00742	Coagulation facto	Hydrolase	3.4.21.6	1.14
pdb_00003uop	1	A	P00760	Cationic trypsin	Hydrolase	3.4.21.4	1.14
pdb_00003uqo	1	A	P00760	Cationic trypsin	Hydrolase	3.4.21.4	1.14

PDB ID	Entity ID	Chain ID(s)	UniProt ID(s)	Protein(s)	EC Class	EC Number(s)	RMSD
pdb_00003uuz	1	A	P00760	Cationic trypsin	Hydrolase	3.4.21.4	1.14
pdb_00004jk5	1	A	P00749	Urokinase-type pl	Hydrolase	3.4.21.73	1.14
pdb_00004os1	1	A	P00749	Urokinase-type pl	Hydrolase	3.4.21.73	1.14
pdb_00004os2	1	A	P00749	Urokinase-type pl	Hydrolase	3.4.21.73	1.14
pdb_00004os6	1	A	P00749	Urokinase-type pl	Hydrolase	3.4.21.73	1.14
pdb_00004zh8	1	A	P00742	Coagulation facto	Hydrolase	3.4.21.6	1.14
pdb_00004zhm	2	B	P00749	Urokinase-type pl	Hydrolase	3.4.21.73	1.14
pdb_00005fxl	1	A	P00760	CATIONIC TRYPSI	Hydrolase	3.4.21.4	1.14
pdb_00005za8	1	A	P00749	Urokinase-type pl	Hydrolase	3.4.21.73	1.14
pdb_00006fid	1	A	P00760	Cationic trypsin	Hydrolase	3.4.21.4	1.14
pdb_00006qih	1	A	P00760	Cationic trypsin	Hydrolase	3.4.21.4	1.14
pdb_00007brz	1	A	P00760	Cationic trypsin	Hydrolase	3.4.21.4	1.14
pdb_00007bs3	1	A	P00760	Cationic trypsin	Hydrolase	3.4.21.4	1.14
pdb_00007zrt	1	A	P00749	Urokinase-type pl	Hydrolase	3.4.21.73	1.14
pdb_00001c2j	1	A	P00760	TRYPSIN	Hydrolase	3.4.21.4	1.15
pdb_00001o32	1	A	P00760	BETA-TRYPSIN	Hydrolase	3.4.21.4	1.15
pdb_00001owj	1	A	P00749	Urokinase-type pl	Hydrolase	3.4.21.73	1.15
pdb_00002wub	1	C	Q04756	HEPATOCYTE GR	Hydrolase	3.4.21	1.15
pdb_00003a81	1	A	P00760	Cationic trypsin	Hydrolase	3.4.21.4	1.15
pdb_00003qk1	1	A	P00760	Cationic trypsin	Hydrolase	3.4.21.4	1.15
pdb_00003rxc	1	A	P00760	Cationic trypsin	Hydrolase	3.4.21.4	1.15
pdb_00003rxp	1	A	P00760	Cationic trypsin	Hydrolase	3.4.21.4	1.15
pdb_00004mnw	1	A	P00749	Urokinase-type pl	Hydrolase	3.4.21.73	1.15
pdb_00004x1r	2	B	P00749	Urokinase-type pl	Hydrolase	3.4.21.73	1.15
pdb_00005lh4	1	A	P00760	Cationic trypsin	Hydrolase	3.4.21.4	1.15
pdb_00005zae	1	A	P00749	Urokinase-type pl	Hydrolase	3.4.21.73	1.15
pdb_00005zaf	1	A	P00749	Urokinase-type pl	Hydrolase	3.4.21.73	1.15
pdb_00006l05	1	A	P00749	Urokinase-type pl	Hydrolase	3.4.21.73	1.15
pdb_00007bsa	1	A	P00760	Cationic trypsin	Hydrolase	3.4.21.4	1.15
pdb_00007z25	1	A	P00760	Serine protease 1	Hydrolase	3.4.21.4	1.15
pdb_00007z2i	1	A	P00760	Serine protease 1	Hydrolase	3.4.21.4	1.15
pdb_00001ppc	1	A	P00760	TRYPSIN	Hydrolase	3.4.21.4	1.16
pdb_00001vj9	1	A	P00749	plasminogen acti	Hydrolase	3.4.21.73	1.16
pdb_00002a32	1	A	P00761	Trypsin	Hydrolase	3.4.21.4	1.16
pdb_00002jkh	1	A	P00742	ACTIVATED FACT	Hydrolase	3.4.21.6	1.16
pdb_00002nwn	1	A	P00749	Plasminogen acti	Hydrolase	3.4.21.73	1.16
pdb_00003m35	1	A	P00760	Cationic trypsin	Hydrolase	3.4.21.4	1.16
pdb_00003mhw	1	A	P00749	Urokinase-type pl	Hydrolase	3.4.21.73	1.16

PDB ID	Entity ID	Chain ID(s)	UniProt ID(s)	Protein(s)	EC Class	EC Number(s)	RMSD
pdb_00005zag	1	A	P00749	Urokinase-type pl	Hydrolase	3.4.21.73	1.16
pdb_00007bs8	1	A	P00760	Cationic trypsin	Hydrolase	3.4.21.4	1.16
pdb_00007q0w	1	A	P00760	Cationic trypsin	Hydrolase	3.4.21.4	1.16
pdb_00009jd0	2	D	O15393	Transmembrane p	Hydrolase	3.4.21.122	1.16
pdb_00001l2e	1	A	Q92876	Kallikrein 6	Hydrolase	3.4.21	1.17
pdb_00001lo6	1	A	Q92876	Kallikrein 6	Hydrolase	3.4.21	1.17
pdb_00001pph	1	A	P00760	TRYPSIN	Hydrolase	3.4.21.4	1.17
pdb_00001uto	1	A	P00760	TRYPSINOGEN	Hydrolase	3.4.21.4	1.17
pdb_00001y3v	1	A	P00760	Trypsin, cationic	Hydrolase	3.4.21.4	1.17
pdb_00002bok	1	A	P00742	COAGULATION FA	Hydrolase	3.4.21.6	1.17
pdb_00003k2u	1	A	Q04756	Hepatocyte growt	Hydrolase	3.4.21	1.17
pdb_00004x1q	2	B	P00749	Urokinase-type pl	Hydrolase	3.4.21.73	1.17
pdb_00005wxo	1	A	P00749	Urokinase-type pl	Hydrolase	3.4.21.73	1.17
pdb_00006qhb	1	A	Q92876	Kallikrein-6	Hydrolase	3.4.21	1.17
pdb_00007z9f	2	B,D	P07478	Trypsin-2	Hydrolase	3.4.21.4	1.17
pdb_00009awl	1	A	P00760	Cationic trypsin	Hydrolase	3.4.21.4	1.17
pdb_00009u8g	2	B	O15393	Transmembrane p	Hydrolase	3.4.21.122	1.17
pdb_00001pfx	1	A	P16293	FACTOR IXA	Hydrolase	3.4.21.22	1.18
pdb_00001yyy	1	A	P00760	TRYPSIN	Hydrolase	3.4.21.4	1.18
pdb_00001zzz	1	A	P00760	TRYPSIN	Hydrolase	3.4.21.4	1.18
pdb_00002cji	1	A	P00742	ACTIVATED FACT	Hydrolase	3.4.21.6	1.18
pdb_00002p3t	2	B	P00742	Coagulation facto	Hydrolase	3.4.21.6	1.18
pdb_00002viq	1	A	P00749	UROKINASE-TYPE	Hydrolase	3.4.21.73	1.18
pdb_00002y5h	1	A	P00742	ACTIVATED FACT	Hydrolase	3.4.21.6	1.18
pdb_00003m61	1	A	P00749	Urokinase-type pl	Hydrolase	3.4.21.73	1.18
pdb_00003ncl	1	A	Q9Y5Y6	Suppressor of tur	Hydrolase	3.4.21.109	1.18
pdb_00003rxj	1	A	P00760	Cationic trypsin	Hydrolase	3.4.21.4	1.18
pdb_00003unq	1	A	P00760	Cationic trypsin	Hydrolase	3.4.21.4	1.18
pdb_00004y76	1	A	P00742	Coagulation facto	Hydrolase	3.4.21.6	1.18
pdb_00004y7a	1	A	P00742	Coagulation facto	Hydrolase	3.4.21.6	1.18
pdb_00004y7b	1	A	P00742	Coagulation facto	Hydrolase	3.4.21.6	1.18
pdb_00005wxp	1	A	P00749	Urokinase-type pl	Hydrolase	3.4.21.73	1.18
pdb_00005zc5	1	A	P00749	Urokinase-type pl	Hydrolase	3.4.21.73	1.18
pdb_00006b77	2	B	P00748	Coagulation facto	Hydrolase	3.4.21.38	1.18
pdb_00006nmb	1	A	P00749	Urokinase-type pl	Hydrolase	3.4.21.73	1.18
pdb_00006qha	1	A	Q92876	Kallikrein-6	Hydrolase	3.4.21	1.18
pdb_00007qft	1	B	Q92876	Kallikrein-6	Hydrolase	3.4.21	1.18
pdb_00007vm6	1	A	P00749	Urokinase-type pl	Hydrolase	3.4.21.73	1.18

PDB ID	Entity ID	Chain ID(s)	UniProt ID(s)	Protein(s)	EC Class	EC Number(s)	RMSD
pdb_00008est	1	A	P00772	PORCINE PANCRI	Hydrolase	3.4.21.36	1.18
pdb_00001c2h	1	A	P00760	TRYPSIN	Hydrolase	3.4.21.4	1.19
pdb_00001f5k	1	A	P00749	UROKINASE-TYPE	Hydrolase	3.4.21.73	1.19
pdb_00001g3d	1	A	P00760	BETA-TRYPSIN	Hydrolase	3.4.21.4	1.19
pdb_00001ioe	1	A	P00742	COAGULATION FA	Hydrolase	3.4.21.6	1.19
pdb_00001iqf	1	A	P00742	coagulation Facto	Hydrolase	3.4.21.6	1.19
pdb_00001lqe	1	A	P00760	TRYPSIN	Hydrolase	3.4.21.4	1.19
pdb_00001w13	1	A	P00749	UROKINASE-TYPE	Hydrolase	3.4.21.73	1.19
pdb_00002j38	1	A	P00742	ACTIVATED FACT	Hydrolase	3.4.21.6	1.19
pdb_00002qy0	2	B	P00736	Complement C1r	Hydrolase	3.4.21.41	1.19
pdb_00002uwl	1	A	P00742	COAGULATION FA	Hydrolase	3.4.21.6	1.19
pdb_00002y5g	1	A	P00742	ACTIVATED FACT	Hydrolase	3.4.21.6	1.19
pdb_00002y7x	1	A	P00742	ACTIVATED FACT	Hydrolase	3.4.21.6	1.19
pdb_00002y81	1	A	P00742	ACTIVATED FACT	Hydrolase	3.4.21.6	1.19
pdb_00004dva	1	A	P00749	Urokinase-type pl	Hydrolase	3.4.21.73	1.19
pdb_00004x1p	1	A	P00749	Urokinase-type pl	Hydrolase	3.4.21.73	1.19
pdb_00005wxf	1	A	P00749	Urokinase-type pl	Hydrolase	3.4.21.73	1.19
pdb_00009awh	1	A	P00760	Cationic trypsin	Hydrolase	3.4.21.4	1.19
pdb_00009pyf	2	B	P00749	Urokinase-type pl	Hydrolase	3.4.21.73	1.19
pdb_00001sqa	1	A	P00749	Urokinase-type pl	Hydrolase	3.4.21.73	1.2
pdb_00001x7a	1	A	P16293	Coagulation Facto	Hydrolase	3.4.21.22	1.2
pdb_00003ati	1	A	P00760	Cationic trypsin	Hydrolase	3.4.21.4	1.2
pdb_00004h42	1	A	P00749	Urokinase-type pl	Hydrolase	3.4.21.73	1.2
pdb_00006ag9	1	A	P00749	Urokinase-type pl	Hydrolase	3.4.21.73	1.2
pdb_00006q1u	1	A	P00747	Plasminogen	Hydrolase	3.4.21.7	1.2
pdb_00006xvd	1	A	P00749	Urokinase-type pl	Hydrolase	3.4.21.73	1.2
pdb_00007vo7	1	A	P00760	Cationic trypsin	Hydrolase	3.4.21.4	1.2
pdb_00008iyv	1	A	P00760	Cationic trypsin	Hydrolase	3.4.21.4	1.2
pdb_00008v04	2	B	O15393	Transmembrane p	Hydrolase	3.4.21.122	1.2
pdb_00001az8	1	A	P00760	TRYPSIN	Hydrolase	3.4.21.4	1.21
pdb_00002j4i	1	A	P00742	COAGULATION FA	Hydrolase	3.4.21.6	1.21
pdb_00002uwp	1	A	P00742	COAGULATION FA	Hydrolase	3.4.21.6	1.21
pdb_00004fuj	1	A	P00749	Urokinase-type pl	Hydrolase	3.4.21.73	1.21
pdb_00004y6d	1	A	P00742	Coagulation facto	Hydrolase	3.4.21.6	1.21
pdb_00004y71	1	A	P00742	Coagulation facto	Hydrolase	3.4.21.6	1.21
pdb_00004zkr	1	A	P00749	Urokinase-type pl	Hydrolase	3.4.21.73	1.21
pdb_00005z1c	1	A	P00749	Urokinase-type pl	Hydrolase	3.4.21.73	1.21
pdb_00006t9t	1	A	Q9Y5Y6	Suppressor of tur	Hydrolase	3.4.21.109	1.21

PDB ID	Entity ID	Chain ID(s)	UniProt ID(s)	Protein(s)	EC Class	EC Number(s)	RMSD
pdb_00007wb8	1	A	P00760	Cationic trypsin	Hydrolase	3.4.21.4	1.21
pdb_00007wqx	1	A	P98073	Enteropeptidase	Hydrolase	3.4.21.9	1.21
pdb_00001eax	1	A	Q9Y5Y6	SUPPRESSOR OF	Hydrolase	3.4.21.109	1.22
pdb_00001f5l	1	A	P00749	UROKINASE-TYPE	Hydrolase	3.4.21.73	1.22
pdb_00001y5u	1	A	P00760	Trypsin, cationic	Hydrolase	3.4.21.4	1.22
pdb_00002vh6	1	A	P00742	ACTIVATED FACT	Hydrolase	3.4.21.6	1.22
pdb_00002wyg	1	A	P00742	ACTIVATED FACT	Hydrolase	3.4.21.6	1.22
pdb_00002y5f	1	A	P00742	ACTIVATED FACT	Hydrolase	3.4.21.6	1.22
pdb_00002y80	1	A	P00742	ACTIVATED FACT	Hydrolase	3.4.21.6	1.22
pdb_00003mwi	1	A	P00749	Urokinase-type pl	Hydrolase	3.4.21.73	1.22
pdb_00003rxs	1	A	P00760	Cationic trypsin	Hydrolase	3.4.21.4	1.22
pdb_00004jyt	1	A	Q9Y5Y6	Suppressor of tur	Hydrolase	3.4.21.109	1.22
pdb_00004jzi	1	A	Q9Y5Y6	Suppressor of tur	Hydrolase	3.4.21.109	1.22
pdb_00005ms3	1	A	O60259	Kallikrein-8	Hydrolase	3.4.21.118	1.22
pdb_00006d3x	1	A	P00747	Plasminogen	Hydrolase	3.4.21.7	1.22
pdb_00009jd1	1	A	O15393	Transmembrane p	Hydrolase	3.4.21.122	1.22
pdb_00001e36	1	A	P00772	ELASTASE	Hydrolase	3.4.21.36	1.23
pdb_00001iqn	1	A	P00742	coagulation Facto	Hydrolase	3.4.21.6	1.23
pdb_00002bq6	2	B	P00742	FACTOR XA	Hydrolase	3.4.21.6	1.23
pdb_00002vh0	1	A	P00742	ACTIVATED FACT	Hydrolase	3.4.21.6	1.23
pdb_00003aau	1	A	P00760	Cationic trypsin	Hydrolase	3.4.21.4	1.23
pdb_00003kgp	1	A	P00749	Urokinase-type pl	Hydrolase	3.4.21.73	1.23
pdb_00003rxt	1	A	P00760	Cationic trypsin	Hydrolase	3.4.21.4	1.23
pdb_00004jz1	1	A	Q9Y5Y6	Suppressor of tur	Hydrolase	3.4.21.109	1.23
pdb_00006x0t	1	A	P00748	Coagulation facto	Hydrolase	3.4.21.38	1.23
pdb_00007vm5	1	A	P00749	Urokinase-type pl	Hydrolase	3.4.21.73	1.23
pdb_00001e35	1	A	P00772	ELASTASE	Hydrolase	3.4.21.36	1.24
pdb_00002y82	1	A	P00742	ACTIVATED FACT	Hydrolase	3.4.21.6	1.24
pdb_00003khv	1	A	P00749	Urokinase-type pl	Hydrolase	3.4.21.73	1.24
pdb_00003kid	1	A	P00749	Urokinase-type pl	Hydrolase	3.4.21.73	1.24
pdb_00004zkn	1	A	P00749	Urokinase-type pl	Hydrolase	3.4.21.73	1.24
pdb_00004zko	1	A	P00749	Urokinase-type pl	Hydrolase	3.4.21.73	1.24
pdb_00006ag3	1	A	P00749	Urokinase-type pl	Hydrolase	3.4.21.73	1.24
pdb_00007wa2	1	A	P00760	Cationic trypsin	Hydrolase	3.4.21.4	1.24
pdb_00001iqg	1	A	P00742	coagulation Facto	Hydrolase	3.4.21.6	1.25
pdb_00001tnl	1	A	P00760	TRYPSIN	Hydrolase	3.4.21.4	1.25
pdb_00003atm	1	A	P00760	Cationic trypsin	Hydrolase	3.4.21.4	1.25
pdb_00004d8n	1	A	Q92876	Kallikrein-6	Hydrolase	3.4.21	1.25

PDB ID	Entity ID	Chain ID(s)	UniProt ID(s)	Protein(s)	EC Class	EC Number(s)	RMSD
pdb_00006x0s	1	A	P00748	Coagulation facto	Hydrolase	3.4.21.38	1.25
pdb_00007bs1	1	A	P00760	Cationic trypsin	Hydrolase	3.4.21.4	1.25
pdb_00001iqm	1	A	P00742	coagulation Facto	Hydrolase	3.4.21.6	1.26
pdb_00002h9e	1	A	P00742	Coagulation facto	Hydrolase	3.4.21.6	1.26
pdb_00003p8g	1	A	Q9Y5Y6	ST14 protein	Hydrolase	3.4.21.109	1.26
pdb_00004o9v	1	A	Q9Y5Y6	Suppressor of tun	Hydrolase	3.4.21.109	1.26
pdb_00004r0i	1	A	Q9Y5Y6	Suppressor of tun	Hydrolase	3.4.21.109	1.26
pdb_00009ct1	1	C	P00761	Trypsin	Hydrolase	3.4.21.4	1.26
pdb_00002y7z	1	A	P00742	ACTIVATED FACT	Hydrolase	3.4.21.6	1.27
pdb_00005zah	1	A	P00749	Urokinase-type pl	Hydrolase	3.4.21.73	1.27
pdb_00007bs0	1	A	P00760	Cationic trypsin	Hydrolase	3.4.21.4	1.27
pdb_00001e37	1	A	P00772	ELASTASE	Hydrolase	3.4.21.36	1.28
pdb_00001os8	1	A	P00775	trypsin	Hydrolase	3.4.21.4	1.28
pdb_00004gly	1	A	P00749	Urokinase-type pl	Hydrolase	3.4.21.73	1.28
pdb_00007bs2	1	A	P00760	Cationic trypsin	Hydrolase	3.4.21.4	1.28
pdb_00008ol9	3	C	P00740	Coagulation facto	Hydrolase	3.4.21.22	1.28
pdb_00009awv	1	A	P00760	Cationic trypsin	Hydrolase	3.4.21.4	1.28
pdb_00009awz	1	A	P00760	Cationic trypsin	Hydrolase	3.4.21.4	1.28
pdb_00001tnj	1	A	P00760	TRYPSIN	Hydrolase	3.4.21.4	1.29
pdb_00001tnk	1	A	P00760	TRYPSIN	Hydrolase	3.4.21.4	1.29
pdb_00003ig6	2	B	P00749	Urokinase-type pl	Hydrolase	3.4.21.73	1.29
pdb_00007q0x	1	A	P00760	Cationic trypsin	Hydrolase	3.4.21.4	1.29
pdb_00009awu	1	A	P00760	Cationic trypsin	Hydrolase	3.4.21.4	1.29
pdb_00001hv7	1	A	P00772	ELASTASE 1	Hydrolase	3.4.21.36	1.3
pdb_00001s0r	1	A	P00760	Trypsinogen	Hydrolase	3.4.21.4	1.3
pdb_00001tnh	1	A	P00760	TRYPSIN	Hydrolase	3.4.21.4	1.3
pdb_00001tni	1	A	P00760	TRYPSIN	Hydrolase	3.4.21.4	1.3
pdb_00002o8t	1	A	P00749	Urokinase plasmi	Hydrolase	3.4.21.73	1.3
pdb_00003oy6	1	A	P00749	Urokinase-type pl	Hydrolase	3.4.21.73	1.3
pdb_00007wb7	1	A	P00760	Cationic trypsin	Hydrolase	3.4.21.4	1.3
pdb_00009awf	1	A	P00760	Cationic trypsin	Hydrolase	3.4.21.4	1.3
pdb_00001iqh	1	A	P00742	coagulation Facto	Hydrolase	3.4.21.6	1.31
pdb_00003oy5	1	A	P00749	Urokinase-type pl	Hydrolase	3.4.21.73	1.31
pdb_00003plk	1	A	P00760	Cationic trypsin	Hydrolase	3.4.21.4	1.31
pdb_00003pwc	1	A	P00760	Cationic trypsin	Hydrolase	3.4.21.4	1.31
pdb_00005k0h	1	A	P00742	Coagulation facto	Hydrolase	3.4.21.6	1.31
pdb_00006b6t	1	A	P00760	Cationic trypsin	Hydrolase	3.4.21.4	1.31
pdb_00007brw	1	A	P00760	Cationic trypsin	Hydrolase	3.4.21.4	1.31

PDB ID	Entity ID	Chain ID(s)	UniProt ID(s)	Protein(s)	EC Class	EC Number(s)	RMSD
pdb_00007mzt	2	B	P00736	Complement C1r	Hydrolase	3.4.21.41	1.31
pdb_00001qgf	1	A	P00772	ELASTASE	Hydrolase	3.4.21.36	1.32
pdb_00001s0q	1	A	P00760	Trypsinogen	Hydrolase	3.4.21.4	1.32
pdb_00003e1x	1	A	Q16651	Prostasin	Hydrolase	3.4.21	1.32
pdb_00003i78	1	A	P00775	Trypsin	Hydrolase	3.4.21.4	1.32
pdb_00003ox7	1	A	P00749	Urokinase-type pl	Hydrolase	3.4.21.73	1.32
pdb_00004aba	1	A	P00760	CATIONIC TRYPSI	Hydrolase	3.4.21.4	1.32
pdb_00004abb	1	A	P00760	CATIONIC TRYPSI	Hydrolase	3.4.21.4	1.32
pdb_00004abd	1	A	P00760	CATIONIC TRYPSI	Hydrolase	3.4.21.4	1.32
pdb_00004abe	1	A	P00760	CATIONIC TRYPSI	Hydrolase	3.4.21.4	1.32
pdb_00004abh	1	A	P00760	CATIONIC TRYPSI	Hydrolase	3.4.21.4	1.32
pdb_00005xg4	1	A	P00749	Urokinase-type pl	Hydrolase	3.4.21.73	1.32
pdb_00001c5m	1	A	P00742	PROTEIN (COAGU	Hydrolase	3.4.21.6	1.33
pdb_00001e34	1	A	P00772	ELASTASE	Hydrolase	3.4.21.36	1.33
pdb_00001j8a	1	A	P00760	TRYPSINOGEN, C	Hydrolase	3.4.21.4	1.33
pdb_00001tx7	1	A	P00760	Trypsinogen	Hydrolase	3.4.21.4	1.33
pdb_00001v2p	1	A	P00760	Trypsin	Hydrolase	3.4.21.4	1.33
pdb_00002qn5	2	B	P00760	Cationic trypsin	Hydrolase	3.4.21.4	1.33
pdb_00003pwb	1	A	P00760	Cationic trypsin	Hydrolase	3.4.21.4	1.33
pdb_00006xyk	1	A	P00760	Cationic trypsin	Hydrolase	3.4.21.4	1.33
pdb_00001gi4	1	A	P00760	BETA-TRYPSIN	Hydrolase	3.4.21.4	1.34
pdb_00001v2k	1	A	P00760	Trypsin	Hydrolase	3.4.21.4	1.34
pdb_00001ym0	1	A	Q3HR18	fibrinotic enzyme	Hydrolase	3.4.21	1.34
pdb_00005yc6	1	A	P00749	Urokinase-type pl	Hydrolase	3.4.21.73	1.34
pdb_00007brv	1	A	P00760	Cationic trypsin	Hydrolase	3.4.21.4	1.34
pdb_00007wba	1	A	P00760	Cationic trypsin	Hydrolase	3.4.21.4	1.34
pdb_00001j17	1	A	P00763	Trypsin II, anionic	Hydrolase	3.4.21.4	1.35
pdb_00001k1i	1	A	P00760	TRYPSIN	Hydrolase	3.4.21.4	1.35
pdb_00001tng	1	A	P00760	TRYPSIN	Hydrolase	3.4.21.4	1.35
pdb_00001y59	1	A	P00760	Trypsin, cationic	Hydrolase	3.4.21.4	1.35
pdb_00003plb	1	A	P00760	Cationic trypsin	Hydrolase	3.4.21.4	1.35
pdb_00004abf	1	A	P00760	CATIONIC TRYPSI	Hydrolase	3.4.21.4	1.35
pdb_00007wb6	1	A	P00760	Cationic trypsin	Hydrolase	3.4.21.4	1.35
pdb_00009azk	1	C	P00747	Plasminogen	Hydrolase	3.4.21.7	1.35
pdb_00002otv	1	A	P00760	Cationic trypsin	Hydrolase	3.4.21.4	1.36
pdb_00001ql7	1	A	P00760	TRYPSIN	Hydrolase	3.4.21.4	1.37
pdb_00002wyj	1	A	P00742	ACTIVATED FACT	Hydrolase	3.4.21.6	1.37
pdb_00009awq	1	A	P00760	Cationic trypsin	Hydrolase	3.4.21.4	1.37

PDB ID	Entity ID	Chain ID(s)	UniProt ID(s)	Protein(s)	EC Class	EC Number(s)	RMSD
pdb_00001v2q	1	A	P00760	Trypsin	Hydrolase	3.4.21.4	1.38
pdb_00001oss	1	A	P00775	trypsin	Hydrolase	3.4.21.4	1.39
pdb_00003beu	1	B	P00775	Trypsin	Hydrolase	3.4.21.4	1.39
pdb_00003dfj	1	A	Q16651	Prostasin	Hydrolase	3.4.21	1.39
pdb_00003i77	1	A	P00775	Trypsin	Hydrolase	3.4.21.4	1.39
pdb_00001e38	1	A	P00772	ELASTASE	Hydrolase	3.4.21.36	1.4
pdb_00001iqk	1	A	P00742	coagulation Facto	Hydrolase	3.4.21.6	1.4
pdb_00001ql9	1	A	P00763	TRYPSIN	Hydrolase	3.4.21.4	1.4
pdb_00001v2o	1	A	P00760	Trypsin	Hydrolase	3.4.21.4	1.4
pdb_00001v2r	1	A	P00760	Trypsin	Hydrolase	3.4.21.4	1.4
pdb_00001v2w	1	A	P00760	Trypsin	Hydrolase	3.4.21.4	1.41
pdb_00001v2t	1	A	P00760	Trypsin	Hydrolase	3.4.21.4	1.42
pdb_00003aav	1	A	P00760	Cationic trypsin	Hydrolase	3.4.21.4	1.42
pdb_00004abg	1	A	P00760	CATIONIC TRYPSI	Hydrolase	3.4.21.4	1.42
pdb_00008izi	1	A	P00760	Cationic trypsin	Hydrolase	3.4.21.4	1.42
pdb_00001ql8	1	A	P00760	TRYPSIN	Hydrolase	3.4.21.4	1.43
pdb_00001sgt	1	A	P00775	TRYPSIN	Hydrolase	3.4.21.4	1.43
pdb_00001iqi	1	A	P00742	coagulation Facto	Hydrolase	3.4.21.6	1.46
pdb_00001btp	1	A	P00760	BETA-TRYPSIN	Hydrolase	3.4.21.4	1.47
pdb_00001iqe	1	A	P00742	coagulation Facto	Hydrolase	3.4.21.6	1.48
pdb_00003gyl	1	A	Q16651	Prostasin	Hydrolase	3.4.21	1.48
pdb_00007vm4	1	A	P00749	Urokinase-type pla	Hydrolase	3.4.21.73	1.55
pdb_00007dzd	1	A	P00749	Urokinase-type pla	Hydrolase	3.4.21.73	1.58
pdb_00003uir	1	B	P00747	Plasmin light chai	Hydrolase	3.4.21.7	1.6
pdb_00004wwy	1	B	P07477	Trypsin-1	Hydrolase	3.4.21.4	1.63
pdb_00001p0s	2	B	P00742	Coagulation facto	Hydrolase	3.4.21.6	1.74
pdb_00001tgt	1	A	P00760	TRYPSINOGEN	Hydrolase	3.4.21.4	1.75

PDB ID - Unique UniProt hits	Entity ID	Chain ID(s)	UniProt ID(s)	Protein(s)	EC Class	EC Number(s)	RMSD
pdb_00001pq7	1	A	P35049	Trypsin	Hydrolase	3.4.21.4	0.09
pdb_00002agg	1	A	P00760	Cationic trypsin	Hydrolase	3.4.21.4	0.17
pdb_00005dj7	1	A	Q54137	Trypsin			0.17
pdb_00005xw1	2	A,B	P00761	Trypsin	Hydrolase	3.4.21.4	0.2
pdb_00004m7g	1	A	P24664	Trypsin-like protease	Hydrolase	3.4.21.4	0.21
pdb_00007ahv	4	D	P00740	Coagulation factor IX	Hydrolase	3.4.21.22	0.21
pdb_00002f9n	1	C	Q15661	alpha I tryptase	Hydrolase	3.4.21.59	0.22
pdb_00001w12	1	A	P00749	UROKINASE-TYPE PLASMINOGEN ACTIVATOR	Hydrolase	3.4.21.73	0.23
pdb_00007xyd	2	D	O15393	Transmembrane protease serine 2 catalytic chain	Hydrolase	3.4.21.122	0.23
pdb_00002fs9	1	D	P20231	Tryptase beta-2	Hydrolase	3.4.21.59	0.24
pdb_00009i24	1	A	P00742	Activated factor Xa heavy chain	Hydrolase	3.4.21.6	0.24
pdb_00001z8g	1	A	P05981	Serine protease hepsin	Hydrolase	3.4.21.106	0.26
pdb_00008zvx	1	A		snFPITE-n2 A chain			0.26
pdb_00001hay	1	A	P00772	ELASTASE 1	Hydrolase	3.4.21.36	0.27
pdb_00003e0p	1	A	Q16651	Prostasin	Hydrolase	3.4.21	0.28
pdb_00004dgj	1	A	P98073	Enteropeptidase catalytic light chain	Hydrolase	3.4.21.9	0.28
pdb_00001j15	1	A	P00763	Trypsin II, anionic	Hydrolase	3.4.21.4	0.29
pdb_00002stb	1	A	P35031	PROTEIN (TRYPSIN)	Hydrolase	3.4.21.4	0.29
pdb_00001bth	2	D	P00734	THROMBIN	Hydrolase	3.4.21.5	0.31
pdb_00001ekb	2	B	P98072	ENTEROPEPTIDASE	Hydrolase	3.4.21.9	0.32
pdb_00004iso	1	A	Q9Y5Y6	Suppressor of tumorigenicity 14 protein	Hydrolase	3.4.21.109	0.32
pdb_00005nx1	1	A	Q92876	Kallikrein-6	Hydrolase	3.4.21	0.33
pdb_00007fbp	1	A	P00748	Coagulation factor XIIa light chain	Hydrolase	3.4.21.38	0.33
pdb_00002psx	1	A	Q9Y337	Kallikrein-5	Hydrolase	3.4.21	0.34
pdb_00001yc0	1	A	Q04756	Hepatocyte growth factor activator	Hydrolase	3.4.21	0.35
pdb_00006d3y	1	A	P00747	Plasminogen	Hydrolase	3.4.21.7	0.35
pdb_00007pzo	1	A	P39675	mite allergen Der p 3	Hydrolase	3.4.21	0.35
pdb_00004bnr	1	A	Q52V24	HEPATOPANCREAS TRYPSIN	Hydrolase	3.4.21.4	0.36
pdb_00001a5i	1	A	P98119	PLASMINOGEN ACTIVATOR	Hydrolase	3.4.21.68	0.39
pdb_00006kd5	2	B	Q9BYE2	Transmembrane protease serine 13	Hydrolase	3.4.21	0.39
pdb_00002zpq	1	A	Q8AV11	Anionic trypsin	Hydrolase	3.4.21.4	0.4
pdb_00004bxw	1	A	Q56VR3	FACTOR XA	Hydrolase	3.4.21.6	0.44
pdb_00005ms4	1	B	O60259	Kallikrein-8	Hydrolase	3.4.21.118	0.44
pdb_00001fiz	1	A	P08001	BETA-ACROSIN HEAVY CHAIN	Hydrolase	3.4.21.10	0.47
pdb_00001bda	1	B	P00750	SINGLE CHAIN TISSUE TYPE PLASMINOGEN ACTI	Hydrolase	3.4.21.68	0.49
pdb_00002oq5	1	A	Q9UL52	Transmembrane protease, serine 11E	Hydrolase	3.4.21	0.5
pdb_00002eek	1	A	P16049	Trypsin-1	Hydrolase	3.4.21.4	0.55
pdb_00002fmj	1	A	P00775	Trypsin	Hydrolase	3.4.21.4	0.62
pdb_00002zpr	1	A	B3Y8K5	Anionic trypsin	Hydrolase	3.4.21.4	0.64
pdb_00001fiw	1	A	Q9GL10	BETA-ACROSIN HEAVY CHAIN	Hydrolase	3.4.21.10	0.69
pdb_00001a0j	1	A	P35033	TRYPSIN	Hydrolase	3.4.21.4	0.74
pdb_00001trn	1	A	P07477	TRYPSIN	Hydrolase	3.4.21.4	0.83
pdb_00002zps	1	A	B3Y8K6	Anionic trypsin	Hydrolase	3.4.21.4	0.96
pdb_00003w94	1	A	A4UWM5	Enteropeptidase-1			1
pdb_00003k6y	1	A	P9WHR9	POSSIBLE MEMBRANE-ASSOCIATED SERINE PROT	Hydrolase	3.4.21	1.07
pdb_00001kig	1	A	P00743	FACTOR XA	Hydrolase	3.4.21.6	1.08
pdb_00007z9f	2	B,D	P07478	Trypsin-2	Hydrolase	3.4.21.4	1.17
pdb_00001pfx	1	A	P16293	FACTOR IXA	Hydrolase	3.4.21.22	1.18
pdb_00002qy0	2	B	P00736	Complement C1r subcomponent	Hydrolase	3.4.21.41	1.19
pdb_00001ym0	1	A	Q3HR18	fibrinotic enzyme component B	Hydrolase	3.4.21	1.34
