# Supplementary material for: RCSB protein data Bank: Next‐generation advanced search for exploration of experimental structures and computed structure models
Source: Protein Sci. 2026 Jul 28;35(8):e70731. doi: 10.1002/pro.70731 (PMC13410952; doi:10.1002/pro.70731)
Supplement: Supplementary file 2 — TABLE S2. Distribution of functional classes and Enzyme Commission (EC) annotations among PDB sequences corresponding to the best‐matching Zn2+‐binding motifs. The table was generated using a Python script that queries the 3D Motif search API; the script is provided as a publicly available example in the RCSB PDB training resources repository: C2HC‐zinc‐binding‐proteins.py. [file PRO-35-e70731-s002.pdf]

PDB ID	Entity ID	Chain ID(s)	UniProt ID(s)	Protein(s)	EC Class	EC Number(s)	RMSD
pdb_00008gcb	2	B	Q96EQ8	E3 ubiquitin-protein ligase RNF125	Transferase	2.3.2.27	0.16
pdb_00005lpi	1	D	O60308	Centrosomal protein of 104 kDa			0.17
pdb_00005vf0	2	B	Q9NS91	E3 ubiquitin-protein ligase RAD18	Transferase	2.3.2.27	0.19
pdb_00007d0o	1	A	O95251	Histone acetyltransferase KAT7	Transferase	2.3.1.48	0.19
pdb_00008gbq	2	B	Q96EQ8	E3 ubiquitin-protein ligase RNF125	Transferase	2.3.2.27	0.19
pdb_00006mak	1	A	O95251	Histone acetyltransferase KAT7	Transferase	2.3.1.48	0.2
pdb_00002ou2	1	A	Q92993	Histone acetyltransferase HTATIP	Transferase	2.3.1	0.24
pdb_00009dzn	2	B	Q92794	Histone acetyltransferase KAT6A	Transferase	2.3.1.48	0.24
pdb_00004zux	9	W	Q03067	SAGA-associated factor 11			0.25
pdb_00004zux	11	Y	P53165	SAGA-associated factor 73			0.41
pdb_00002lo2	1	A	Q03067	SAGA-associated factor 11			0.26
pdb_00002ozu	1	A	Q92794	Histone acetyltransferase MYST3	Transferase	2.3.1.48	0.26
pdb_00006pdg	1	A	Q9H7Z6	Histone acetyltransferase KAT8	Transferase	2.3.1	0.26
pdb_00002y0m	1	A	Q9H7Z6	PROBABLE HISTONE ACETYLTRANSFERASE MYST	Transferase	2.3.1, 2.3.1.48	0.29
pdb_00003hcs	1	A	Q9Y4K3	TNF receptor-associated factor 6	Transferase	2.3.2.27	0.29
pdb_00006pdb	1	A	Q9H7Z6	Histone acetyltransferase KAT8	Transferase	2.3.1	0.29
pdb_00007d0s	1	A	O95251	Histone acetyltransferase KAT7	Transferase	2.3.1.48	0.29
pdb_00009ooe	1	A	Q9H7Z6	Histone acetyltransferase KAT8	Transferase	2.3.1	0.29
pdb_00006owh	1	A	Q9H7Z6	Histone acetyltransferase KAT8	Transferase	2.3.1	0.33
pdb_00009dou	1	A	A0A803VHT8	R2Tg retrotransposon ORF			0.33
pdb_00007mex	4	D	P19812	E3 ubiquitin-protein ligase UBR1	Transferase	2.3.2.27	0.34
pdb_00002yre	1	A	Q8TB52	F-box only protein 30			0.35
pdb_00006maj	1	A	O95251	Histone acetyltransferase KAT7	Transferase	2.3.1.48	0.36
pdb_00008dd5	1	A	Q92794	Histone acetyltransferase KAT6A	Transferase	2.3.1.48	0.37
pdb_00002gqj	1	A	Q96KM6	Zinc finger protein KIAA1196			0.38
pdb_00003m99	4	D	P53165	SAGA-associated factor 73			0.38
pdb_00002d9k	1	A	O14545	FLN29 gene product			0.41
pdb_00007mey	1	A	P19812	E3 ubiquitin-protein ligase UBR1	Transferase	2.3.2.27	0.42
pdb_00008hz2	1	A	Q9Y4K3	TNF receptor-associated factor 6	Transferase	2.3.2.27	0.44
pdb_00002eod	1	A	Q9BUZ4	TNF receptor-associated factor 4	Transferase	2.3.2.27	0.45
pdb_00007od1	1	B	O95376	E3 ubiquitin-protein ligase ARIH2	Transferase	2.3.2.31	0.45
pdb_00009vkw	1	A	P34218	Histone acetyltransferase SAS3	Transferase	2.3.1.48	0.53
pdb_00002yqq	1	A	Q15649	Zinc finger HIT domain-containing protein 3			0.54
pdb_00004kbl	1	A	Q9Y4X5	E3 ubiquitin-protein ligase ARIH1	Transferase	2.3.2.31	0.54
pdb_00005udh	1	C	Q9Y4X5	E3 ubiquitin-protein ligase ARIH1	Transferase	2.3.2.31	0.58
pdb_00009nl2	1	A	A0A8C3F8X6	R2 retrotransposon protein	Hydrolase	3.1.26.4	0.58
pdb_00005diz	1	B	Q680B9	Proteinaceous RNase P 2	Hydrolase	3.1.26.5	0.59
pdb_00002m9y	1	A	Q9Y4X5	E3 ubiquitin-protein ligase ARIH1	Transferase	2.3.2.31	0.63
pdb_00005d5n	2	B	F5HFZ4	Virion egress protein UL31 homolog			0.68
pdb_000022tg	1	A	Q9H7Z6	Histone acetyltransferase KAT8	Transferase	2.3.1, 2.3.1.48	0.7
pdb_00007egc	24	X	Q00403	Transcription initiation factor IIB	Transferase	2.3.1.48	0.73
pdb_00008edg	4	I	Q25438	Hermes transposase			0.73
pdb_00007ela	1	A	A0A097F4I8	RING finger protein Z			0.76
pdb_00007onu	2	E	O15091	Mitochondrial ribonuclease P catalytic subunit	Hydrolase	3.1.26.5	0.76
pdb_00007t7i	2	D	P0CK47	Nuclear egress protein 1			0.8
pdb_00004ggk	1	A	Q5SWZ9	Mitochondrial cardiolipin hydrolase	Hydrolase	3.1.4	0.81

PDB ID	Entity ID	Chain ID(s)	UniProt ID(s)	Protein(s)	EC Class	EC Number(s)	RMSD
pdb_00005b3h	3	E	Q700D2	Zinc finger protein JACKDAW			0.82
pdb_00007xfg	1	A	Q09472	Histone acetyltransferase p300	Transferase	2.3.1, 2.3.1.48	0.83
pdb_00004ggj	1	A	Q5SWZ9	Mitochondrial cardiolipin hydrolase	Hydrolase	3.1.4	0.85
pdb_00008b6h	4	D	Q23FF5	Cytochrome C oxidase subunit Vb protein			0.91
pdb_00008bqs	100	HF	Q23FF5	Cytochrome C oxidase subunit Vb protein			0.92
pdb_00005vo0	1	D	Q6IWL4	TNF receptor-associated factor 6	Transferase	2.3.2.27	0.93
pdb_00008esz	2	B	Q9VMU0	NADH dehydrogenase [ubiquinone] iron-sulfur prot			0.93
pdb_00007ckl	2	B	A0A097F4I8	RING finger protein Z			0.97
pdb_00007el9	2	E	Q6UY77	RING finger protein Z			1
pdb_00009bpb	14	N	P04037	Cytochrome c oxidase subunit 4, mitochondrial			1.02
pdb_00007elc	2	B	Q6UY77	RING finger protein Z			1.12
pdb_00007ckm	2	B	Q6UY77	RING finger protein Z			1.13
pdb_00007eg8	14	N	Q00403	Transcription initiation factor IIB	Transferase	2.3.1.48	1.14
pdb_00005iy6	13	M	Q00403	Transcription initiation factor IIB	Transferase	2.3.1.48	1.17
pdb_00005iya	13	M	Q00403	Transcription initiation factor IIB	Transferase	2.3.1.48	1.17
pdb_00007ega	14	N	Q00403	Transcription initiation factor IIB	Transferase	2.3.1.48	1.17
pdb_00002jvm	1	A	Q3IZ23	Uncharacterized protein			1.18
pdb_00008grq	7	K	P38398	Breast cancer type 1 susceptibility protein	Transferase	2.3.2.27	1.3
pdb_00005iyd	13	M	Q00403	Transcription initiation factor IIB	Transferase	2.3.1.48	1.34
pdb_00006t7f	1	A	O75592	E3 ubiquitin-protein ligase MYCBP2	Transferase	2.3.2.33	1.34
pdb_00008glw	2	D	P13991	Transposon Tn7 transposition protein TnsD			1.36
pdb_00007aoh	7	G	Q1PIU7	Protein H4			1.39
pdb_00008ymj	1	AC	Q913A6	Isoform S of Large envelope protein			1.45
pdb_00002d8s	1	A	Q5T0T0	cellular modulator of immune recognition	Transferase	2.3.2.27	1.53
pdb_00008e6v	1	A	O94759	Transient receptor potential cation channel subfam			1.54
pdb_00009dx1	5	F		GATOR2 complex protein WDR24			1.56
pdb_00006ric	1	A	B9U1I2	DNA-dependent RNA polymerase subunit rpo147	Transferase	2.7.7.6	1.57
pdb_00008fac	1	A	Q38SD2	Leucine-rich repeat serine/threonine-protein kinase	Transferase	2.7.11.1	1.57
pdb_00007nvw	4	D	P51948	CDK-activating kinase assembly factor MAT1			1.58
pdb_00008ewi	1	C	O95071	E3 ubiquitin-protein ligase UBR5	Transferase	2.3.2.26	1.58
pdb_00007nvx	4	D	P51948	CDK-activating kinase assembly factor MAT1			1.59
pdb_00007xyz	1	B	Q1XH17	Tripartite motif-containing protein 72	Transferase	2.3.2.27	1.59
pdb_00007nvr	4	D	P51948	CDK-activating kinase assembly factor MAT1			1.61
pdb_00007nvy	4	D	P51948	CDK-activating kinase assembly factor MAT1			1.62
pdb_00005jut	46	TA	P0CX27	eL42 (yeast L42)			1.65
pdb_00006w9d	2	B	Q9NVW2	E3 ubiquitin-protein ligase RLIM	Transferase	2.3.2.27	1.65
pdb_00008a5y	6	I	Q12157	Anaphase-promoting complex subunit 11	Ligase	6.3.2	1.66
pdb_00009dx0	3	D		Protein SEC13 homolog			1.67
pdb_00008upf	6	I	Q8IYW5	E3 ubiquitin-protein ligase RNF168	Transferase	2.3.2.27	1.68
pdb_00009dde	8	L	Q99496	E3 ubiquitin-protein ligase RING2	Transferase	2.3.2.27	1.68
pdb_00009dde	7	K	P35226	Polycomb complex protein BMI-1			1.76
pdb_00009i1i	1	A	E9Q555	E3 ubiquitin-protein ligase RNF213	Hydrolase, Trans	2.3.2, 2.3.2.27, 3.6.4	1.68
pdb_00005ulk	3	C	Q6ZSG1	E3 ubiquitin-protein ligase RNF165	Transferase	2.3.2.27	1.69
pdb_00009eg1	8	H	Q9UBF6	RING-box protein 2	Transferase	2.3.2.27, 2.3.2.32	1.7
pdb_00006zu5	30	DA		eL42			1.72
pdb_00008i3x	1	A	B8AY66	RING-type domain-containing protein			1.72

PDB ID	Entity ID	Chain ID(s)	UniProt ID(s)	Protein(s)	EC Class	EC Number(s)	RMSD
pdb_00005ulh	3	C	Q6ZSG1	E3 ubiquitin-protein ligase RNF165	Transferase	2.3.2.27	1.73
pdb_00009ipu	7	K	Q8IYW5	E3 ubiquitin-protein ligase RNF168	Transferase	2.3.2.27	1.73
pdb_00002d54	1	A	P23395	Methionyl-tRNA synthetase	Ligase	6.1.1.10	1.74
pdb_00009dgg	7	K	P35226	Polycomb complex protein BMI-1			1.74
pdb_00009dgg	8	L	Q99496	E3 ubiquitin-protein ligase RING2	Transferase	2.3.2.27	1.8
pdb_00001rmd	1	A	P15919	RAG1	Hydrolase, Trans	2.3.2.27, 3.1	1.76
pdb_00004r8p	7	M	P35226	Polycomb complex protein BMI-1			1.76
pdb_00004r8p	8	L	P61077, Q99496	E3 ubiquitin-protein ligase RING2, Ubiquitin-conjuga	Transferase	2.3.2.23, 2.3.2.24, 2.:	1.76
pdb_00001x4j	1	A	Q9H0F5	RING finger protein 38	Transferase	2.3.2.27	1.77
pdb_00009dby	8	K	P35226	Polycomb complex protein BMI-1			1.77
pdb_00009dby	9	L	Q99496	E3 ubiquitin-protein ligase RING2	Transferase	2.3.2.27	1.78
pdb_00007t92	3	C	G2Q0E2	Peroxin-10	Transferase	2.3.2.27	1.78
pdb_00002y1m	1	A	P22681	E3 UBIQUITIN-PROTEIN LIGASE	Transferase	2.3.2.27	1.79
pdb_00005d1m	2	B	Q96BH1	E3 ubiquitin-protein ligase RNF25	Transferase	2.3.2.27	1.79
pdb_00006t83	76	XB	P0CX27	60S ribosomal protein L42-A			1.79
pdb_00008sn5	7	K	Q8IYW5	E3 ubiquitin-protein ligase RNF168	Transferase	2.3.2.27	1.79
pdb_00001fbv	1	A	P22681	SIGNAL TRANSDUCTION PROTEIN CBL	Transferase	2.3.2.27	1.8
pdb_00005d1k	2	B	Q96BH1	E3 ubiquitin-protein ligase RNF25	Transferase	2.3.2.27	1.8
pdb_00006qaj	1	A	P00720, Q13263	Endolysin,Transcription intermediary factor 1-beta	Hydrolase, Trans	2.3.2.27, 3.2.1.17	1.8
pdb_00002ckl	2	B	Q9CQJ4	UBIQUITIN LIGASE PROTEIN RING2	Transferase	2.3.2.27	1.81
pdb_00002ckl	1	A	P25916	POLYCOMB GROUP RING FINGER PROTEIN 4			1.88
pdb_00005ait	1	A	O88846	E3 UBIQUITIN-PROTEIN LIGASE RNF4	Transferase	2.3.2.27	1.81
pdb_00006m2c	2	E	Q969V5	Mitochondrial ubiquitin ligase activator of NFKB 1	Transferase	2.3.2.27	1.81
pdb_00008pp7	1	A	P35226	Polycomb complex protein BMI-1			1.81
pdb_00001z6u	1	B	Q96PU4	Np95-like ring finger protein isoform b	Transferase	2.3.2.27	1.82
pdb_00004ppe	1	A	P78317	E3 ubiquitin-protein ligase RNF4	Transferase	2.3.2.27	1.82
pdb_00005d1l	2	B	Q96BH1	E3 ubiquitin-protein ligase RNF25	Transferase	2.3.2.27	1.82
pdb_00005eya	2	B	Q14258	Tripartite motif-containing 25 variant	Transferase	2.3.2.27	1.82
pdb_00005fer	1	D	Q14258	E3 ubiquitin/ISG15 ligase TRIM25	Ligase, Transfer	2.3.2.27, 6.3.2	1.82
pdb_00005j3x	1	B	P22681	E3 ubiquitin-protein ligase CBL	Transferase	2.3.2.27	1.82
pdb_00006wi8	1	B	P35226, Q99496	E3 ubiquitin-protein ligase RING2,Polycomb comple	Transferase	2.3.2.27	1.82
pdb_00007nd1	1	A	Q99496	E3 ubiquitin-protein ligase RING2	Transferase	2.3.2.27	1.82
pdb_00007nd1	2	B	P35226	Polycomb complex protein BMI-1			1.9
pdb_00008sn4	7	K	Q8IYW5	E3 ubiquitin-protein ligase RNF168	Transferase	2.3.2.27	1.82
pdb_00009qru	1	A	O60291	Isoform 4 of E3 ubiquitin-protein ligase MGRN1	Transferase	2.3.2.27	1.82
pdb_00009xrl	15	RA	Q8VDF2	E3 ubiquitin-protein ligase UHRF1	Transferase	2.3.2.27	1.82
pdb_00003rpg	3	C	Q99496	E3 ubiquitin-protein ligase RING2	Transferase	2.3.2.27	1.83
pdb_00003rpg	2	B	P35226	Polycomb complex protein BMI-1			1.9
pdb_00004a49	1	A	P22681	E3 ubiquitin-protein ligase CBL	Transferase	2.3.2.27	1.83
pdb_00005hkx	1	A	P22681	E3 ubiquitin-protein ligase CBL	Transferase	2.3.2.27	1.83
pdb_00002lgv	1	A	P62877	E3 ubiquitin-protein ligase RBX1	Transferase	2.3.2.27, 2.3.2.32	1.84
pdb_00003eb6	1	A	Q13489	Baculoviral IAP repeat-containing protein 3	Transferase	2.3.2.27	1.84
pdb_00004s3o	2	D	Q99496	E3 ubiquitin-protein ligase RING2	Transferase	2.3.2.27	1.84
pdb_00004s3o	3	E	Q86SE9	Polycomb group RING finger protein 5			1.85
pdb_00005yuf	1	C	P29590	Protein PML	Transferase	2.3.2	1.84
pdb_00005zi6	1	H	Q5U5Q3	RNA-binding E3 ubiquitin-protein ligase MEX3C	Transferase	2.3.2.27	1.84

PDB ID	Entity ID	Chain ID(s)	UniProt ID(s)	Protein(s)	EC Class	EC Number(s)	RMSD
pdb_00007bvw	1	A	Q9M622	AT5G66160 protein			1.84
pdb_00007ojx	1	A	Q9H0F5	E3 ubiquitin-protein ligase RNF38	Transferase	2.3.2.27	1.84
pdb_00008a61	6	I	Q12157	Anaphase-promoting complex subunit 11	Ligase	6.3.2	1.84
pdb_00008sn2	7	K	Q8IYW5	E3 ubiquitin-protein ligase RNF168	Transferase	2.3.2.27	1.84
pdb_00009qf5	61	KB	Q8U475	Large ribosomal subunit protein eL42			1.84
pdb_00002kiz	1	A	Q6ZNA4	E3 ubiquitin-protein ligase Arkadia	Transferase	2.3.2.27	1.85
pdb_00003vk6	1	A	Q9JIY2	E3 ubiquitin-protein ligase Hakai	Transferase	2.3.2.27	1.85
pdb_00006s53	3	K	P19474	E3 ubiquitin-protein ligase TRIM21	Transferase	2.3.2.27	1.85
pdb_00006y22	1	A	Q86UW9	Probable E3 ubiquitin-protein ligase DTX2	Transferase	2.3.2.27	1.85
pdb_00007oni	3	C	Q9UBF6	RING-box protein 2	Transferase	2.3.2.27, 2.3.2.32	1.85
pdb_00007pai	49	WA	P78015	50S ribosomal protein L33 1			1.85
pdb_00001ncp	2	A,B	P24736	HIV-1 P7 NUCLEOCAPSID PROTEIN			1.86
pdb_00002h0d	1	A	P35226	B lymphoma Mo-MLV insertion region			1.86
pdb_00002yhn	1	A	Q8WY64	E3 UBIQUITIN-PROTEIN LIGASE MYLIP	Transferase	2.3.2.27	1.86
pdb_00003t6p	1	A	Q13490	Baculoviral IAP repeat-containing protein 2	Transferase	2.3.2.27	1.86
pdb_00003zni	1	I	Q13191	E3 UBIQUITIN-PROTEIN LIGASE CBL-B	Transferase	2.3.2.27	1.86
pdb_00004r7e	1	A	Q07457	E3 ubiquitin-protein ligase BRE1	Transferase	2.3.2.27	1.86
pdb_00005trb	1	A	Q5VTR2	E3 ubiquitin-protein ligase BRE1A	Transferase	2.3.2.27	1.86
pdb_00008a3t	15	S	Q12157	Anaphase-promoting complex subunit 11	Ligase	6.3.2	1.86
pdb_00008smw	7	K	Q8IYW5	E3 ubiquitin-protein ligase RNF168	Transferase	2.3.2.27	1.86
pdb_00008sn0	7	K	Q8IYW5	E3 ubiquitin-protein ligase RNF168	Transferase	2.3.2.27	1.86
pdb_00008sn7	7	K	Q8IYW5	E3 ubiquitin-protein ligase RNF168	Transferase	2.3.2.27	1.86
pdb_00009gaw	16	T	Q9NYG5	Anaphase-promoting complex subunit 11			1.86
pdb_00002mt5	1	A	Q9NYG5	Anaphase-promoting complex subunit 11			1.87
pdb_00002y1n	1	C	P22681	E3 UBIQUITIN-PROTEIN LIGASE	Transferase	2.3.2.27	1.87
pdb_00003vu8	1	A	P23395	Methionine--tRNA ligase	Ligase	6.1.1.10	1.87
pdb_00004ayc	1	A	O76064	E3 UBIQUITIN-PROTEIN LIGASE RNF8	Transferase	2.3.2.27	1.87
pdb_00004ayc	2	B	O76064	E3 UBIQUITIN-PROTEIN LIGASE RNF8	Transferase	2.3.2.27	1.9
pdb_00004r2y	1	A,B	Q9NYG5	Anaphase-promoting complex subunit 11			1.87
pdb_00004whv	2	B	O76064	E3 ubiquitin-protein ligase RNF8	Transferase	2.3.2.27	1.87
pdb_00005d0k	2	B	Q6ZSG1	RING finger protein 165	Transferase	2.3.2.27	1.87
pdb_00005tqr	2	B	G0RYC6, G0SDW4	Histone-lysine N-methyltransferase EZH2, Polycom	Transferase	2.1.1.356	1.87
pdb_00006w9a	2	D	Q9NVW2	E3 ubiquitin-protein ligase RLIM	Transferase	2.3.2.27	1.87
pdb_00007eiz	1	A	P0DTD1	RNA-directed RNA polymerase	Transferase	2.7.7.48	1.87
pdb_00007zu0	3	C	P27801	Vacuolar membrane protein PEP3			1.87
pdb_00008a38	1	B	Q9C040	Tripartite motif-containing protein 2	Transferase	2.3.2.27	1.87
pdb_00008qtj	1	A	Q13191	E3 ubiquitin-protein ligase CBL-B	Transferase	2.3.2.27	1.87
pdb_00008smx	7	K	Q8IYW5	E3 ubiquitin-protein ligase RNF168	Transferase	2.3.2.27	1.87
pdb_00008uq9	1	B	P04908, P61077, Q	E3 ubiquitin-protein ligase RNF168,Ubiquitin-conjuç	Transferase	2.3.2.23, 2.3.2.24, 2.:	1.87
pdb_00009fr0	1	A,C	P83789	CO-methylating acetyl-CoA synthase	Transferase	2.3.1.169	1.87
pdb_00009t7v	2	B	Q9UBF6	RING-box protein 2	Transferase	2.3.2.27, 2.3.2.32	1.87
pdb_00002ecg	1	A	P98170	Baculoviral IAP repeat-containing protein 4	Transferase	2.3.2.27	1.88
pdb_00002ecl	1	A	Q9UBF6	RING-box protein 2	Transferase	2.3.2.27, 2.3.2.32	1.88
pdb_00002ecw	1	A	P15533	Tripartite motif-containing protein 30			1.88
pdb_00002y43	1	B	Q9NS91	E3 UBIQUITIN-PROTEIN LIGASE RAD18	Transferase	2.3.2.27	1.88
pdb_00002yho	1	G	Q8WY64	E3 UBIQUITIN-PROTEIN LIGASE MYLIP	Transferase	2.3.2.27	1.88

PDB ID	Entity ID	Chain ID(s)	UniProt ID(s)	Protein(s)	EC Class	EC Number(s)	RMSD
pdb_00004gb0	1	A	Q8IYW5	E3 ubiquitin-protein ligase RNF168	Transferase	2.3.2.27	1.88
pdb_00004orh	3	J	O76064	E3 ubiquitin-protein ligase RNF8	Transferase	2.3.2.27	1.88
pdb_00005d0i	1	B	Q6ZSG1	RING finger protein 165	Transferase	2.3.2.27	1.88
pdb_00005o76	1	A	P22681	E3 ubiquitin-protein ligase CBL	Transferase	2.3.2.27	1.88
pdb_00006v9q	3	D	A0A6I8WFX7	TniQ family protein			1.88
pdb_00006w7z	2	B	Q9NVW2	E3 ubiquitin-protein ligase RLIM	Transferase	2.3.2.27	1.88
pdb_00006wi7	1	A	P35226, Q99496	E3 ubiquitin-protein ligase RING2, Polycomb compl	Transferase	2.3.2.27	1.88
pdb_00007r71	1	A	P0CG48, Q6ZSG1	Ubiquitin,E3 ubiquitin-protein ligase RNF165	Transferase	2.3.2.27	1.88
pdb_00007zj3	1	G	Q9C040	Tripartite motif-containing protein 2	Transferase	2.3.2.27	1.88
pdb_00008ams	2	C	Q9C040	Tripartite motif-containing protein 2	Transferase	2.3.2.27	1.88
pdb_00008grm	6	I	P35226	COMMD3 protein			1.88
pdb_00008qtg	1	A	Q13191	E3 ubiquitin-protein ligase CBL-B	Transferase	2.3.2.27	1.88
pdb_00008qtk	1	A	Q13191	E3 ubiquitin-protein ligase CBL-B	Transferase	2.3.2.27	1.88
pdb_00009jta	2	B	Q63HN8	E3 ubiquitin-protein ligase RNF213	Hydrolase, Trans	2.3.2, 2.3.2.27, 3.6.4	1.88
pdb_00009q8y	2	C	Q9H0F5	Isoform 2 of E3 ubiquitin-protein ligase RNF38	Transferase	2.3.2.27	1.88
pdb_000013rz	1	B	Q8WY64	E3 ubiquitin-protein ligase MYLIP	Transferase	2.3.2.27	1.89
pdb_00001v87	1	A	Q8R3P2	Deltex protein 2	Transferase	2.3.2.27	1.89
pdb_00002ect	1	A	Q91YL2	RING finger protein 126	Transferase	2.3.2.27	1.89
pdb_00004a4c	1	A	P22681	E3 UBIQUITIN-PROTEIN LIGASE CBL	Transferase	2.3.2.27	1.89
pdb_00004auq	2	E	Q96CA5	BACULOVIRAL IAP REPEAT-CONTAINING PROTEIN	Transferase	2.3.2.27	1.89
pdb_00005d0m	2	B	Q6ZSG1	RING finger protein 165	Transferase	2.3.2.27	1.89
pdb_00005jg6	1	A	Q9NYG5	Anaphase-promoting complex subunit 11			1.89
pdb_00005kkl	2	B	G0RYC6, G0SDW4,	Putative uncharacterized protein,Histone H3.1 pept	Transferase	2.1.1.356	1.89
pdb_00005olm	1	A	P19474	E3 ubiquitin-protein ligase TRIM21	Transferase	2.3.2.27	1.89
pdb_00005vk3	2	B	G0RYC6, G0SDW4	Histone-lysine N-methyltransferase EZH2,Polycoml	Transferase	2.1.1.356	1.89
pdb_00006fga	1	A	P19474	E3 ubiquitin-protein ligase TRIM21	Transferase	2.3.2.27	1.89
pdb_00006hpr	1	A	Q13490	Baculoviral IAP repeat-containing protein 2	Transferase	2.3.2.27	1.89
pdb_00007r70	1	A	P0CG48, Q6ZSG1	Ubiquitin,E3 ubiquitin-protein ligase RNF165	Transferase	2.3.2.27	1.89
pdb_00007ynx	1	B	Q96PM5	RING finger and CHY zinc finger domain-containing	Transferase	2.3.2.27	1.89
pdb_00008qth	1	A	Q13191	E3 ubiquitin-protein ligase CBL-B	Transferase	2.3.2.27	1.89
pdb_00008w59	1	A	P98170	E3 ubiquitin-protein ligase XIAP	Transferase	2.3.2.27	1.89
pdb_00008w5a	1	B	P98170	E3 ubiquitin-protein ligase XIAP	Transferase	2.3.2.27	1.89
pdb_00009fqi	1	A	Q13191	E3 ubiquitin-protein ligase CBL-B	Transferase	2.3.2.27	1.89
pdb_00009fqj	1	A	Q13191	E3 ubiquitin-protein ligase CBL-B	Transferase	2.3.2.27	1.89
pdb_00009lwf	2	I	Q96S15	GATOR2 complex protein WDR24	Transferase	2.3.2.27	1.89
pdb_000013sd	1	B	Q8WY64	E3 ubiquitin-protein ligase MYLIP	Transferase	2.3.2.27	1.9
pdb_000013se	1	B	Q8WY64	E3 ubiquitin-protein ligase MYLIP	Transferase	2.3.2.27	1.9
pdb_000013sg	1	B	Q8WY64	E3 ubiquitin-protein ligase MYLIP	Transferase	2.3.2.27	1.9
pdb_000013si	1	B	Q8WY64	E3 ubiquitin-protein ligase MYLIP	Transferase	2.3.2.27	1.9
pdb_000013sk	1	D	Q8WY64	E3 ubiquitin-protein ligase MYLIP	Transferase	2.3.2.27	1.9
pdb_000013sl	1	B	Q8WY64	E3 ubiquitin-protein ligase MYLIP	Transferase	2.3.2.27	1.9
pdb_000013sn	1	A	Q8WY64	E3 ubiquitin-protein ligase MYLIP	Transferase	2.3.2.27	1.9
pdb_000013ss	1	D	Q8WY64	E3 ubiquitin-protein ligase MYLIP	Transferase	2.3.2.27	1.9
pdb_000013sz	1	A	Q8WY64	E3 ubiquitin-protein ligase MYLIP	Transferase	2.3.2.27	1.9
pdb_000013tb	1	A	Q8WY64	E3 ubiquitin-protein ligase MYLIP	Transferase	2.3.2.27	1.9
pdb_00004a4b	1	A	P22681	E3 UBIQUITIN-PROTEIN LIGASE CBL	Transferase	2.3.2.27	1.9

PDB ID	Entity ID	Chain ID(s)	UniProt ID(s)	Protein(s)	EC Class	EC Number(s)	RMSD
pdb_00004ic3	1	B	P98170	E3 ubiquitin-protein ligase XIAP	Transferase	2.3.2.27	1.9
pdb_00004qpl	1	A	Q9CZW6	E3 ubiquitin-protein ligase RNF146	Transferase	2.3.2.27	1.9
pdb_00004v3k	3	F	Q9H0F5	E3 UBIQUITIN-PROTEIN LIGASE RNF38	Transferase	2.3.2.27	1.9
pdb_00005iea	1	C	P34945, Q0PF16	Tripartite motif-containing protein 5, Serine--tRNA li	Transferase	2.3.2.27	1.9
pdb_00005ndg	78	ZB	P0CX27	60S ribosomal protein L42-A			1.9
pdb_00005wf7	2	B	G0RYC6, G0SDW4	Histone-lysine-N-methyltransferase EZH2, Polycom	Transferase	2.1.1.356	1.9
pdb_00005wfd	2	B	G0RYC6, G0SDW4	Histone-lysine-N-methyltransferase EZH2, Polycom	Transferase	2.1.1.356	1.9
pdb_00006m2d	1	C	Q969V5	Mitochondrial ubiquitin ligase activator of NFKB 1	Transferase	2.3.2.27	1.9
pdb_00006y2x	1	A	Q86UW9	Probable E3 ubiquitin-protein ligase DTX2	Transferase	2.3.2.27	1.9
pdb_00006y5p	1	A	Q86Y01	E3 ubiquitin-protein ligase DTX1	Transferase	2.3.2.27	1.9
pdb_00008pjn	1	A	Q9H871	E3 ubiquitin-protein transferase RMND5A	Transferase	2.3.2.27	1.9
pdb_00008uqc	1	A	P04908, P61077, Q	E3 ubiquitin-protein ligase RNF168,Ubiquitin-conjuç	Transferase	2.3.2.23, 2.3.2.24, 2.:	1.9
pdb_00009q88	1	A	Q9H0F5	E3 ubiquitin-protein ligase RNF38	Transferase	2.3.2.27	1.9
pdb_00009sa1	1	B	Q8WY64	E3 ubiquitin-protein ligase MYLIP	Transferase	2.3.2.27	1.9
pdb_000013sa	1	B	Q8WY64	E3 ubiquitin-protein ligase MYLIP	Transferase	2.3.2.27	1.91
pdb_000013sc	1	B	Q8WY64	E3 ubiquitin-protein ligase MYLIP	Transferase	2.3.2.27	1.91
pdb_000013sf	1	D	Q8WY64	E3 ubiquitin-protein ligase MYLIP	Transferase	2.3.2.27	1.91
pdb_000013sh	1	A	Q8WY64	E3 ubiquitin-protein ligase MYLIP	Transferase	2.3.2.27	1.91
pdb_000013sj	1	B	Q8WY64	E3 ubiquitin-protein ligase MYLIP	Transferase	2.3.2.27	1.91
pdb_000013sm	1	B	Q8WY64	E3 ubiquitin-protein ligase MYLIP	Transferase	2.3.2.27	1.91
pdb_000013so	1	D	Q8WY64	E3 ubiquitin-protein ligase MYLIP	Transferase	2.3.2.27	1.91
pdb_000013sr	1	B	Q8WY64	E3 ubiquitin-protein ligase MYLIP	Transferase	2.3.2.27	1.91
pdb_000013st	1	C	Q8WY64	E3 ubiquitin-protein ligase MYLIP	Transferase	2.3.2.27	1.91
pdb_00003eb5	1	A	Q13489	Baculoviral IAP repeat-containing protein 3	Transferase	2.3.2.27	1.91
pdb_00003l11	1	A	Q8IYW5	E3 ubiquitin-protein ligase RNF168	Transferase	2.3.2.27	1.91
pdb_00004tkp	2	B	Q0PF16	Tripartite motif-containing protein 5	Transferase	2.3.2.27	1.91
pdb_00005o6t	1	A	P98170	E3 ubiquitin-protein ligase XIAP	Transferase	2.3.2.27	1.91
pdb_00005zc4	1	B	O43567	E3 ubiquitin-protein ligase RNF13	Transferase	2.3.2.27	1.91
pdb_00006y5n	1	A	Q86Y01	E3 ubiquitin-protein ligase DTX1	Transferase	2.3.2.27	1.91
pdb_00006yxe	1	B	Q86WT6	E3 ubiquitin-protein ligase TRIM69	Transferase	2.3.2.27	1.91
pdb_00007z36	1	B	P00720, Q13263	Endolysin,Transcription intermediary factor 1-beta,I	Hydrolase, Trans	2.3.2.27, 3.2.1.17	1.91
pdb_00008a58	2	C	P19474	E3 ubiquitin-protein ligase TRIM21	Transferase	2.3.2.27	1.91
pdb_00008uq8	1	B	P04908, P61077, Q	E3 ubiquitin-protein ligase RNF168,Ubiquitin-conjuç	Transferase	2.3.2.27	1.91
pdb_00008uqb	1	A	P04908, P61077, Q	E3 ubiquitin-protein ligase RNF168,Ubiquitin-conjuç	Transferase	2.3.2.23, 2.3.2.24, 2.:	1.91
pdb_00009fqh	1	A	Q13191	E3 ubiquitin-protein ligase CBL-B	Transferase	2.3.2.27	1.91
pdb_00009n1f	1	A	Q6ZSG1	E3 ubiquitin-protein ligase ARK2C	Transferase	2.3.2.27	1.91
pdb_00009qo0	13	M	P62877	E3 ubiquitin-protein ligase RBX1	Transferase	2.3.2.27, 2.3.2.32	1.91
pdb_00009sa2	1	B	Q8WY64	E3 ubiquitin-protein ligase MYLIP	Transferase	2.3.2.27	1.91
pdb_000013sb	1	C	Q8WY64	E3 ubiquitin-protein ligase MYLIP	Transferase	2.3.2.27	1.92
pdb_000013sp	1	D	Q8WY64	E3 ubiquitin-protein ligase MYLIP	Transferase	2.3.2.27	1.92
pdb_000013sq	1	A	Q8WY64	E3 ubiquitin-protein ligase MYLIP	Transferase	2.3.2.27	1.92
pdb_000013su	1	B	Q8WY64	E3 ubiquitin-protein ligase MYLIP	Transferase	2.3.2.27	1.92
pdb_000013sv	1	A	Q8WY64	E3 ubiquitin-protein ligase MYLIP	Transferase	2.3.2.27	1.92
pdb_000013sw	1	A	Q8WY64	E3 ubiquitin-protein ligase MYLIP	Transferase	2.3.2.27	1.92
pdb_000013sx	1	A	Q8WY64	E3 ubiquitin-protein ligase MYLIP	Transferase	2.3.2.27	1.92
pdb_000013sy	1	B	Q8WY64	E3 ubiquitin-protein ligase MYLIP	Transferase	2.3.2.27	1.92

PDB ID	Entity ID	Chain ID(s)	UniProt ID(s)	Protein(s)	EC Class	EC Number(s)	RMSD
pdb_000013ta	1	D	Q8WY64	E3 ubiquitin-protein ligase MYLIP	Transferase	2.3.2.27	1.92
pdb_00001iym	1	A	Q9LRB7	EL5	Transferase	2.3.2.27	1.92
pdb_00003p57	4	M	Q09472	Histone acetyltransferase p300	Transferase	2.3.1	1.92
pdb_00004ic2	1	A	P98170	E3 ubiquitin-protein ligase XIAP	Transferase	2.3.2.27	1.92
pdb_00005kji	2	B	G0RYC6, G0SDW4	Putative uncharacterized protein,Zinc finger domai	Transferase	2.1.1.356	1.92
pdb_00005o6c	1	A	O75592	E3 ubiquitin-protein ligase MYCBP2	Transferase	2.3.2.33	1.92
pdb_00005xyn	4	D	P38957	Suppressor of hydroxyurea sensitivity protein 2			1.92
pdb_00007w3r	1	A	Q70CQ2	Ubiquitin carboxyl-terminal hydrolase 34	Hydrolase	3.4.19.12	1.92
pdb_00008gcy	1	A	Q13191	E3 ubiquitin-protein ligase CBL-B	Transferase	2.3.2.27	1.92
pdb_00008qnh	1	A	Q13191	E3 ubiquitin-protein ligase CBL-B	Transferase	2.3.2.27	1.92
pdb_00008sn8	7	K	Q8IYW5	E3 ubiquitin-protein ligase RNF168	Transferase	2.3.2.27	1.92
pdb_00008ylr	43	QA	P0CX27	Large ribosomal subunit protein eL42A			1.92
pdb_00002ecv	1	A	Q9C035	Tripartite motif-containing protein 5	Transferase	2.3.2.27	1.93
pdb_00005bjs	2	B	G0RYC6, G0SDW4	Histone-lysine N-methyltransferase EZH2, Polycom	Transferase	2.1.1.356	1.93
pdb_00005m5g	2	B	G0SDW4	Putative uncharacterized protein	Transferase	2.1.1.356	1.93
pdb_00008uqa	1	A	P04908, P61077, Q	E3 ubiquitin-protein ligase RNF168,Ubiquitin-conjuç	Transferase	2.3.2.23, 2.3.2.24, 2.3	1.93
pdb_00009e4n	4	D	P0A9Q5	Acetyl-coenzyme A carboxylase carboxyl transfera:	Transferase	2.1.3.15	1.93
pdb_00009oti	2	C	Q96S15	GATOR complex protein WDR24	Transferase	2.3.2.27	1.93
pdb_00002l0b	1	A	Q8NG27	E3 ubiquitin-protein ligase Praja-1	Transferase	2.3.2.27	1.94
pdb_00005ndw	66	BE	P0CX27	60S ribosomal protein L42-A			1.94
pdb_00005zbu	2	C	O43567	E3 ubiquitin-protein ligase RNF13	Transferase	2.3.2.27	1.94
pdb_00008qni	1	A	Q13191	E3 ubiquitin-protein ligase CBL-B	Transferase	2.3.2.27	1.94
pdb_00008sn3	7	K	Q8IYW5	E3 ubiquitin-protein ligase RNF168	Transferase	2.3.2.27	1.94
pdb_00008v0d	1	B	P62837, Q86YT6	Ubch5B-RING3 of MIB1 fusion protein	Transferase	2.3.2.23, 2.3.2.24, 2.3	1.94
pdb_00008wqe	5	E	P62877	E3 ubiquitin-protein ligase RBX1, N-terminally proce	Transferase	2.3.2.27, 2.3.2.32	1.94
pdb_00004lad	2	B	Q9UKV5	E3 ubiquitin-protein ligase AMFR	Transferase	2.3.2.36	1.95
pdb_00006skg	62	LB	Q5JE51	50S ribosomal protein L44e			1.95
pdb_00007top	44	RA	P0CX27	60S ribosomal protein L42-A			1.95
pdb_00008iuh	12	L	Q9Y2Y1	DNA-directed RNA polymerase III subunit RPC10			1.95
pdb_00005a31	2	B	Q9NYG5	ANAPHASE-PROMOTING COMPLEX SUBUNIT 11			1.96
pdb_00005g04	2	B	Q9NYG5	ANAPHASE-PROMOTING COMPLEX SUBUNIT 11			1.96
pdb_00005wch	1	D	Q93008	Probable ubiquitin carboxyl-terminal hydrolase FAF	Hydrolase	3.4.19.12	1.96
pdb_00007b7d	78	ZB	P0CX27	60S ribosomal protein L42-A			1.96
pdb_00008sna	7	K	Q8IYW5	E3 ubiquitin-protein ligase RNF168	Transferase	2.3.2.27	1.96
pdb_00008z70	79	AC	P0CX27	Large ribosomal subunit protein eL42A			1.96
pdb_00008z71	79	AC	P0CX27	Large ribosomal subunit protein eL42A			1.96
pdb_00002gfo	1	A	P40818	Ubiquitin carboxyl-terminal hydrolase 8	Hydrolase	3.4.19.12	1.97
pdb_00004ui9	2	B	Q9NYG5	ANAPHASE-PROMOTING COMPLEX SUBUNIT 11			1.97
pdb_00005g05	2	B	Q9NYG5	ANAPHASE-PROMOTING COMPLEX SUBUNIT 11			1.97
pdb_00002ea5	1	A	Q99675	Cell growth regulator with RING finger domain prot			1.98
pdb_00003j77	42	PA	P0CX27	60S ribosomal protein L42			1.98
pdb_00006lnb	4	M	A0A6I8WFX7	Transposition protein TniQ			1.98
pdb_00007rr5	43	QA	A0A6L1B5X0	60S ribosomal protein L42-A			1.98
pdb_00007zs5	6	F	P0CX27	60S ribosomal protein L42-A			1.98
pdb_00008bjq	44	RA	P0CX27	60S ribosomal protein L42-A			1.98
pdb_00002v0g	1	C	Q7SIE4	AMINOACYL-TRNA SYNTHETASE	Ligase	6.1.1.4	1.99

PDB ID	Entity ID	Chain ID(s)	UniProt ID(s)	Protein(s)	EC Class	EC Number(s)	RMSD
pdb_00005lcw	2	B	Q9NYG5	Anaphase-promoting complex subunit 11			1.99
pdb_00006jdu	1	A	J9XNG9	PP1b			1.99
pdb_00009dgu	11	V	A0A4X1TB62	Dynactin subunit 4			1.99
pdb_00008azw	25	Y	A0A1S4AVP7	eL42 (60S ribosomal protein L42)			2

PDB ID - Unique UniProt hits	Entity ID	Chain ID(s)	UniProt ID(s)	Protein(s)	EC Class	EC Number(s)	RMSD
pdb_00008gcb	2	B	Q96EQ8	E3 ubiquitin-protein ligase RNF125	Transferase	2.3.2.27	0.16
pdb_00005lpi	1	D	O60308	Centrosomal protein of 104 kDa			0.17
pdb_00005vf0	2	B	Q9NS91	E3 ubiquitin-protein ligase RAD18	Transferase	2.3.2.27	0.19
pdb_00007d0o	1	A	O95251	Histone acetyltransferase KAT7	Transferase	2.3.1.48	0.19
pdb_00002ou2	1	A	Q92993	Histone acetyltransferase HTATIP	Transferase	2.3.1	0.24
pdb_00009dzn	2	B	Q92794	Histone acetyltransferase KAT6A	Transferase	2.3.1.48	0.24
pdb_00004zux	9	W	Q03067	SAGA-associated factor 11			0.25
pdb_00003m99	4	D	P53165	SAGA-associated factor 73			0.38
pdb_00006pdg	1	A	Q9H7Z6	Histone acetyltransferase KAT8	Transferase	2.3.1	0.26
pdb_00003hcs	1	A	Q9Y4K3	TNF receptor-associated factor 6	Transferase	2.3.2.27	0.29
pdb_00009dou	1	A	A0A803VHT8	R2Tg retrotransposon ORF			0.33
pdb_00007mex	4	D	P19812	E3 ubiquitin-protein ligase UBR1	Transferase	2.3.2.27	0.34
pdb_00002yre	1	A	Q8TB52	F-box only protein 30			0.35
pdb_00002gqj	1	A	Q96KM6	Zinc finger protein KIAA1196			0.38
pdb_00002d9k	1	A	O14545	FLN29 gene product			0.41
pdb_00002eod	1	A	Q9BUZ4	TNF receptor-associated factor 4	Transferase	2.3.2.27	0.45
pdb_00007od1	1	B	O95376	E3 ubiquitin-protein ligase ARIH2	Transferase	2.3.2.31	0.45
pdb_00009vkw	1	A	P34218	Histone acetyltransferase SAS3	Transferase	2.3.1.48	0.53
pdb_00002yqq	1	A	Q15649	Zinc finger HIT domain-containing protein 3			0.54
pdb_00004kbl	1	A	Q9Y4X5	E3 ubiquitin-protein ligase ARIH1	Transferase	2.3.2.31	0.54
pdb_00009nl2	1	A	A0A8C3F8X6	R2 retrotransposon protein	Hydrolase	3.1.26.4	0.58
pdb_00005diz	1	B	Q680B9	Proteinaceous RNase P 2	Hydrolase	3.1.26.5	0.59
pdb_00005d5n	2	B	F5HFZ4	Virion egress protein UL31 homolog			0.68
pdb_00007egc	24	X	Q00403	Transcription initiation factor IIB	Transferase	2.3.1.48	0.73
pdb_00008edg	4	I	Q25438	Hermes transposase			0.73
pdb_00007ela	1	A	A0A097F4I8	RING finger protein Z			0.76
pdb_00007onu	2	E	O15091	Mitochondrial ribonuclease P catalytic subunit	Hydrolase	3.1.26.5	0.76
pdb_00007t7i	2	D	P0CK47	Nuclear egress protein 1			0.8
pdb_00004ggk	1	A	Q5SWZ9	Mitochondrial cardiolipin hydrolase	Hydrolase	3.1.4	0.81
pdb_00005b3h	3	E	Q700D2	Zinc finger protein JACKDAW			0.82
pdb_00007xfg	1	A	Q09472	Histone acetyltransferase p300	Transferase	2.3.1, 2.3.1.48	0.83
pdb_00008b6h	4	D	Q23FF5	Cytochrome C oxidase subunit Vb protein			0.91
pdb_00005vo0	1	D	Q6IWL4	TNF receptor-associated factor 6	Transferase	2.3.2.27	0.93
pdb_00008esz	2	B	Q9VMU0	NADH dehydrogenase [ubiquinone] iron-sulfur			0.93
pdb_00007el9	2	E	Q6UY77	RING finger protein Z			1
pdb_00009bpb	14	N	P04037	Cytochrome c oxidase subunit 4, mitochondria			1.02
pdb_00002jvm	1	A	Q3IZ23	Uncharacterized protein			1.18
pdb_00008grq	7	K	P38398	Breast cancer type 1 susceptibility protein	Transferase	2.3.2.27	1.3
pdb_00006t7f	1	A	O75592	E3 ubiquitin-protein ligase MYCBP2	Transferase	2.3.2.33	1.34
pdb_00008glw	2	D	P13991	Transposon Tn7 transposition protein TnsD			1.36
pdb_00007aoh	7	G	Q1PIU7	Protein H4			1.39
pdb_00008ymj	1	AC	Q913A6	Isoform S of Large envelope protein			1.45
pdb_00002d8s	1	A	Q5T0T0	cellular modulator of immune recognition	Transferase	2.3.2.27	1.53
pdb_00008e6v	1	A	O94759	Transient receptor potential cation channel sul			1.54
pdb_00009dx1	5	F		GATOR2 complex protein WDR24			1.56
pdb_00006ric	1	A	B9U1I2	DNA-dependent RNA polymerase subunit rpo1	Transferase	2.7.7.6	1.57
pdb_00008fac	1	A	Q38SD2	Leucine-rich repeat serine/threonine-protein ki	Transferase	2.7.11.1	1.57
pdb_00007nvw	4	D	P51948	CDK-activating kinase assembly factor MAT1			1.58
pdb_00008ewi	1	C	O95071	E3 ubiquitin-protein ligase UBR5	Transferase	2.3.2.26	1.58

PDB ID - Unique UniProt hits	Entity ID	Chain ID(s)	UniProt ID(s)	Protein(s)	EC Class	EC Number(s)	RMSD
pdb_00007xyz	1	B	Q1XH17	Tripartite motif-containing protein 72	Transferase	2.3.2.27	1.59
pdb_00005jut	46	TA	P0CX27	eL42 (yeast L42)			1.65
pdb_00006w9d	2	B	Q9NVW2	E3 ubiquitin-protein ligase RLIM	Transferase	2.3.2.27	1.65
pdb_00008a5y	6	I	Q12157	Anaphase-promoting complex subunit 11	Ligase	6.3.2	1.66
pdb_00008upf	6	I	Q8IYW5	E3 ubiquitin-protein ligase RNF168	Transferase	2.3.2.27	1.68
pdb_00009dde	8	L	Q99496	E3 ubiquitin-protein ligase RING2	Transferase	2.3.2.27	1.68
pdb_00009dgg	7	K	P35226	Polycomb complex protein BMI-1			1.74
pdb_00009i1i	1	A	E9Q555	E3 ubiquitin-protein ligase RNF213	Hydrolase, Trans	2.3.2, 2.3.2.27, 3.6.4	1.68
pdb_00005ulk	3	C	Q6ZSG1	E3 ubiquitin-protein ligase RNF165	Transferase	2.3.2.27	1.69
pdb_00009eg1	8	H	Q9UBF6	RING-box protein 2	Transferase	2.3.2.27, 2.3.2.32	1.7
pdb_00008i3x	1	A	B8AY66	RING-type domain-containing protein			1.72
pdb_00002d54	1	A	P23395	Methionyl-tRNA synthetase	Ligase	6.1.1.10	1.74
pdb_00001rmd	1	A	P15919	RAG1	Hydrolase, Trans	2.3.2.27, 3.1	1.76
pdb_00004r8p	8	L	P61077, Q99496	E3 ubiquitin-protein ligase RING2, Ubiquitin-co	Transferase	2.3.2.23, 2.3.2.24, 2.3	1.76
pdb_00001x4j	1	A	Q9H0F5	RING finger protein 38	Transferase	2.3.2.27	1.77
pdb_00007t92	3	C	G2Q0E2	Peroxin-10	Transferase	2.3.2.27	1.78
pdb_00002y1m	1	A	P22681	E3 UBIQUITIN-PROTEIN LIGASE	Transferase	2.3.2.27	1.79
pdb_00005d1m	2	B	Q96BH1	E3 ubiquitin-protein ligase RNF25	Transferase	2.3.2.27	1.79
pdb_00006qaj	1	A	P00720, Q13263	Endolysin,Transcription intermediary factor 1-b	Hydrolase, Trans	2.3.2.27, 3.2.1.17	1.8
pdb_00002ckl	2	B	Q9CQJ4	UBIQUITIN LIGASE PROTEIN RING2	Transferase	2.3.2.27	1.81
pdb_00002ckl	1	A	P25916	POLYCOMB GROUP RING FINGER PROTEIN 4			1.88
pdb_00005ait	1	A	O88846	E3 UBIQUITIN-PROTEIN LIGASE RNF4	Transferase	2.3.2.27	1.81
pdb_00006m2c	2	E	Q969V5	Mitochondrial ubiquitin ligase activator of NFK	Transferase	2.3.2.27	1.81
pdb_00001z6u	1	B	Q96PU4	Np95-like ring finger protein isoform b	Transferase	2.3.2.27	1.82
pdb_00004ppe	1	A	P78317	E3 ubiquitin-protein ligase RNF4	Transferase	2.3.2.27	1.82
pdb_00005eya	2	B	Q14258	Tripartite motif-containing 25 variant	Transferase	2.3.2.27	1.82
pdb_00006wi8	1	B	P35226, Q99496	E3 ubiquitin-protein ligase RING2,Polycomb cc	Transferase	2.3.2.27	1.82
pdb_00009qru	1	A	O60291	Isoform 4 of E3 ubiquitin-protein ligase MGRN	Transferase	2.3.2.27	1.82
pdb_00009xrl	15	RA	Q8VDF2	E3 ubiquitin-protein ligase UHRF1	Transferase	2.3.2.27	1.82
pdb_00002lgv	1	A	P62877	E3 ubiquitin-protein ligase RBX1	Transferase	2.3.2.27, 2.3.2.32	1.84
pdb_00003eb6	1	A	Q13489	Baculoviral IAP repeat-containing protein 3	Transferase	2.3.2.27	1.84
pdb_00004s3o	3	E	Q86SE9	Polycomb group RING finger protein 5			1.85
pdb_00005yuf	1	C	P29590	Protein PML	Transferase	2.3.2	1.84
pdb_00005zi6	1	H	Q5U5Q3	RNA-binding E3 ubiquitin-protein ligase MEX3(	Transferase	2.3.2.27	1.84
pdb_00007bvw	1	A	Q9M622	AT5G66160 protein			1.84
pdb_00009qf5	61	KB	Q8U475	Large ribosomal subunit protein eL42			1.84
pdb_00002kiz	1	A	Q6ZNA4	E3 ubiquitin-protein ligase Arkadia	Transferase	2.3.2.27	1.85
pdb_00003vk6	1	A	Q9JIY2	E3 ubiquitin-protein ligase Hakai	Transferase	2.3.2.27	1.85
pdb_00006s53	3	K	P19474	E3 ubiquitin-protein ligase TRIM21	Transferase	2.3.2.27	1.85
pdb_00006y22	1	A	Q86UW9	Probable E3 ubiquitin-protein ligase DTX2	Transferase	2.3.2.27	1.85
pdb_00007pai	49	WA	P78015	50S ribosomal protein L33 1			1.85
pdb_00001ncp	2	A,B	P24736	HIV-1 P7 NUCLEOCAPSID PROTEIN			1.86
pdb_00002yhn	1	A	Q8WY64	E3 UBIQUITIN-PROTEIN LIGASE MYLIP	Transferase	2.3.2.27	1.86
pdb_00003t6p	1	A	Q13490	Baculoviral IAP repeat-containing protein 2	Transferase	2.3.2.27	1.86
pdb_00003zni	1	I	Q13191	E3 UBIQUITIN-PROTEIN LIGASE CBL-B	Transferase	2.3.2.27	1.86
pdb_00004r7e	1	A	Q07457	E3 ubiquitin-protein ligase BRE1	Transferase	2.3.2.27	1.86
pdb_00005trb	1	A	Q5VTR2	E3 ubiquitin-protein ligase BRE1A	Transferase	2.3.2.27	1.86
pdb_00009gaw	16	T	Q9NYG5	Anaphase-promoting complex subunit 11			1.86
pdb_00004ayc	1	A	O76064	E3 UBIQUITIN-PROTEIN LIGASE RNF8	Transferase	2.3.2.27	1.87

PDB ID - Unique UniProt hits	Entity ID	Chain ID(s)	UniProt ID(s)	Protein(s)	EC Class	EC Number(s)	RMSD
pdb_00005tqr	2	B	G0RYC6, G0SDW4	Histone-lysine N-methyltransferase EZH2, Poly	Transferase	2.1.1.356	1.87
pdb_00007eiz	1	A	P0DTD1	RNA-directed RNA polymerase	Transferase	2.7.7.48	1.87
pdb_00007zu0	3	C	P27801	Vacuolar membrane protein PEP3			1.87
pdb_00008a38	1	B	Q9C040	Tripartite motif-containing protein 2	Transferase	2.3.2.27	1.87
pdb_00008uq9	1	B	P04908, P61077, Q	E3 ubiquitin-protein ligase RNF168,Ubiquitin-c	Transferase	2.3.2.23, 2.3.2.24, 2.3	1.87
pdb_00009fr0	1	A,C	P83789	CO-methylating acetyl-CoA synthase	Transferase	2.3.1.169	1.87
pdb_00002ecg	1	A	P98170	Baculoviral IAP repeat-containing protein 4	Transferase	2.3.2.27	1.88
pdb_00002ecw	1	A	P15533	Tripartite motif-containing protein 30			1.88
pdb_00006v9q	3	D	A0A6I8WFX7	TniQ family protein			1.88
pdb_00007r71	1	A	P0CG48, Q6ZSG1	Ubiquitin,E3 ubiquitin-protein ligase RNF165	Transferase	2.3.2.27	1.88
pdb_00009jta	2	B	Q63HN8	E3 ubiquitin-protein ligase RNF213	Hydrolase, Trans	2.3.2, 2.3.2.27, 3.6.4	1.88
pdb_00001v87	1	A	Q8R3P2	Deltex protein 2	Transferase	2.3.2.27	1.89
pdb_00002ect	1	A	Q91YL2	RING finger protein 126	Transferase	2.3.2.27	1.89
pdb_00004auq	2	E	Q96CA5	BACULOVIRAL IAP REPEAT-CONTAINING PRO	Transferase	2.3.2.27	1.89
pdb_00005kkl	2	B	G0RYC6, G0SDW4,	Putative uncharacterized protein,Histone H3.1	Transferase	2.1.1.356	1.89
pdb_00007ynx	1	B	Q96PM5	RING finger and CHY zinc finger domain-conta	Transferase	2.3.2.27	1.89
pdb_00009lwf	2	I	Q96S15	GATOR2 complex protein WDR24	Transferase	2.3.2.27	1.89
pdb_00004qpl	1	A	Q9CZW6	E3 ubiquitin-protein ligase RNF146	Transferase	2.3.2.27	1.9
pdb_00005iea	1	C	P34945, Q0PF16	Tripartite motif-containing protein 5, Serine--tR	Transferase	2.3.2.27	1.9
pdb_00006y5p	1	A	Q86Y01	E3 ubiquitin-protein ligase DTX1	Transferase	2.3.2.27	1.9
pdb_00008pjn	1	A	Q9H871	E3 ubiquitin-protein transferase RMND5A	Transferase	2.3.2.27	1.9
pdb_00004tkp	2	B	Q0PF16	Tripartite motif-containing protein 5	Transferase	2.3.2.27	1.91
pdb_00005zc4	1	B	O43567	E3 ubiquitin-protein ligase RNF13	Transferase	2.3.2.27	1.91
pdb_00006yxe	1	B	Q86WT6	E3 ubiquitin-protein ligase TRIM69	Transferase	2.3.2.27	1.91
pdb_00001iym	1	A	Q9LRB7	EL5	Transferase	2.3.2.27	1.92
pdb_00005xyn	4	D	P38957	Suppressor of hydroxyurea sensitivity protein 2			1.92
pdb_00007w3r	1	A	Q70CQ2	Ubiquitin carboxyl-terminal hydrolase 34	Hydrolase	3.4.19.12	1.92
pdb_00002ecv	1	A	Q9C035	Tripartite motif-containing protein 5	Transferase	2.3.2.27	1.93
pdb_00005m5g	2	B	G0SDW4	Putative uncharacterized protein	Transferase	2.1.1.356	1.93
pdb_00009e4n	4	D	P0A9Q5	Acetyl-coenzyme A carboxylase carboxyl trans	Transferase	2.1.3.15	1.93
pdb_00002l0b	1	A	Q8NG27	E3 ubiquitin-protein ligase Praja-1	Transferase	2.3.2.27	1.94
pdb_00008v0d	1	B	P62837, Q86YT6	Ubch5B-RING3 of MIB1 fusion protein	Transferase	2.3.2.23, 2.3.2.24, 2.3	1.94
pdb_00004lad	2	B	Q9UKV5	E3 ubiquitin-protein ligase AMFR	Transferase	2.3.2.36	1.95
pdb_00006skg	62	LB	Q5JE51	50S ribosomal protein L44e			1.95
pdb_00008iuh	12	L	Q9Y2Y1	DNA-directed RNA polymerase III subunit RPC			1.95
pdb_00005wch	1	D	Q93008	Probable ubiquitin carboxyl-terminal hydrolase	Hydrolase	3.4.19.12	1.96
pdb_00002gfo	1	A	P40818	Ubiquitin carboxyl-terminal hydrolase 8	Hydrolase	3.4.19.12	1.97
pdb_00002ea5	1	A	Q99675	Cell growth regulator with RING finger domain			1.98
pdb_00007rr5	43	QA	A0A6L1B5X0	60S ribosomal protein L42-A			1.98
pdb_00002v0g	1	C	Q7SIE4	AMINOACYL-TRNA SYNTHETASE	Ligase	6.1.1.4	1.99
pdb_00006jdu	1	A	J9XNG9	PP1b			1.99
pdb_00009dgu	11	V	A0A4X1TB62	Dynactin subunit 4			1.99
pdb_00008azw	25	Y	A0A1S4AVP7	eL42 (60S ribosomal protein L42)			2
